# Overview of retrospective data harmonisation in the MINDMAP project: process and results

**DOI:** 10.1136/jech-2020-214259

**Published:** 2020-11-10

**Authors:** Tina W Wey, Dany Doiron, Rita Wissa, Guillaume Fabre, Irina Motoc, J Mark Noordzij, Milagros Ruiz, Erik Timmermans, Frank J van Lenthe, Martin Bobak, Basile Chaix, Steinar Krokstad, Parminder Raina, Erik Reidar Sund, Marielle A Beenackers, Isabel Fortier

**Affiliations:** 1 Maelstrom Research, Research Institute of the McGill University Health Centre, Montreal, Canada; 2 Department of Epidemiology and Biostatistics, Amsterdam UMC, VU University Medical Center, Amsterdam Public Health Research Institute, Amsterdam, Netherlands; 3 Department of Public Health, Erasmus University Medical Center, Rotterdam, Netherlands; 4 Research Department of Epidemiology and Public Health, University College London, London, UK; 5 Department of Human Geography and Spatial Planning, Utrecht University, Utrecht, Netherlands; 6 Sorbonne Université, INSERM, Institut Pierre Louis d’Épidémiologie et de Santé Publique, Nemesis research team, Paris, France; 7 HUNT Research Centre, Department of Public Health and Nursing, Norwegian University of Science and Technology, Levanger, Norway; 8 Levanger Hospital, Nord-Trøndelag Hospital Trust, Levanger, Norway; 9 Department of Health Research Methods, Evidence, and Impact, McMaster University, Hamilton, Canada; 10 McMaster Institute for Research on Aging, McMaster University, Hamilton, Canada; 11 Labarge Centre for Mobility in Aging, McMaster University, Hamilton, Canada; 12 Faculty of Nursing and Health Sciences, Nord Universitet—Levanger Campus, Levanger, Norway

**Keywords:** Cohort studies, epidemiology, longitudinal studies, methodology

## Abstract

**Background:**

The MINDMAP project implemented a multinational data infrastructure to investigate the direct and interactive effects of urban environments and individual determinants of mental well-being and cognitive function in ageing populations. Using a rigorous process involving multiple teams of experts, longitudinal data from six cohort studies were harmonised to serve MINDMAP objectives. This article documents the retrospective data harmonisation process achieved based on the Maelstrom Research approach and provides a descriptive analysis of the harmonised data generated.

**Methods:**

A list of core variables (the DataSchema) to be generated across cohorts was first defined, and the potential for cohort-specific data sets to generate the DataSchema variables was assessed. Where relevant, algorithms were developed to process cohort-specific data into DataSchema format, and information to be provided to data users was documented. Procedures and harmonisation decisions were thoroughly documented.

**Results:**

The MINDMAP DataSchema (v2.0, April 2020) comprised a total of 2841 variables (993 on individual determinants and outcomes, 1848 on environmental exposures) distributed across up to seven data collection events. The harmonised data set included 220 621 participants from six cohorts (10 subpopulations). Harmonisation potential, participant distributions and missing values varied across data sets and variable domains.

**Conclusion:**

The MINDMAP project implemented a collaborative and transparent process to generate a rich integrated data set for research in ageing, mental well-being and the urban environment. The harmonised data set supports a range of research activities and will continue to be updated to serve ongoing and future MINDMAP research needs.

## INTRODUCTION

The MINDMAP (promoting mental well-being and healthy ageing in cities) project offers a multinational data infrastructure to help investigate the opportunities offered by urban environments for the promotion of mental well-being and cognitive function of older individuals. This infrastructure allows multiple investigators to securely and remotely analyse harmonised cohort study data across European and Canadian populations. It also provides approved MINDMAP researchers access to the longitudinal data items and statistical power required to investigate direct and interactive effects of social, environmental and lifestyle determinants of mental health outcomes across different cities, to facilitate both comparative work and pooled analysis of outcomes. To create the MINDMAP infrastructure, cohort-specific data had to be harmonised (processed under a common format allowing co-analysis of data across studies), which is important for ensuring content equivalence and reducing bias due to methodological differences, but presents multiple challenges.^1^ Population sampling frames, participant follow-ups, types of information collected, and variable formats and content vary extensively across cohort studies. It was thus essential to implement a rigorous process to harmonise, integrate and document the core data to be generated.

10.1136/jech-2020-214259.supp1

MINDMAP implemented such a process,^2^ and its current data platform supports a broad range of research activities. The harmonisation teams comprised multiple research groups with a wide range of expertise, who contributed to harmonising data from six population-based cohort studies^3–8^ across Western and Eastern European countries and Canada. The current article details the harmonisation process and provides a descriptive analysis of an early version of the MINDMAP data set (v2.0, April 2020). It describes the harmonisation procedures, summarises variables that were harmonised across studies and provides an overview of key information useful for understanding results presented in this special issue and by future projects using the MINDMAP data set. More broadly, this article illustrates challenges in retrospective harmonisation and considerations for promoting a transparent process to produce collaborative data resources.

## METHODS

The harmonisation process was informed by the retrospective harmonisation guidelines^1^ and used the open-source software Opal 2.16 and Mica 3.9 developed by Maelstrom Research.^9^ The harmonisation teams were composed of epidemiologists, social scientists and statisticians from specialised scientific domains. University College London (UK) harmonised mental health outcomes; VU University Medical Center Amsterdam (Netherlands) harmonised social factors and perceived environment variables, and linked and derived social-environmental variables; the Research Institute of the McGill University Health Centre (Canada) with Erasmus University Medical Center (Netherlands) harmonised sociodemographic, lifestyle and behavioural, and health variables; and Erasmus University Medical Center linked and derived physical-environmental variables. All harmonisation work was done in collaboration across as well as within subject areas to ensure a consistent and coordinated process, and the harmonisation team had weekly to monthly videoconference calls as needed throughout the process.

### Individual-level determinants and outcomes data

The process to harmonise data on individual risk factors (eg, sociodemographic characteristics, life habits) and physical and mental health status across participating cohorts included the following steps.

1. Assemble cohort-specific information and select studies

For each MINDMAP participating cohort, study designs (eg, number of data collection events, population sampling frame, participant selection criteria) and variable data dictionaries were gathered and catalogued online following Maelstrom Research standards.^10^ English versions of documentation were available for all studies, and any ambiguities in language were clarified with the cohort study teams. Six studies (including 10 subpopulations) were included in this version of the MINDMAP data set (v2.0, April 2020) (table 1). Three studies that are part of the MINDMAP consortium were not included in the harmonisation project due to inaccessibility of relevant study-specific individual participant data.

**Table 1:** Overview of MINDMAP participating cohort designs and subpopulations included in the harmonisation project

Subpopulation	Participants (n)*	Country	Recruitment	Data collection mode	Inclusion/exclusion criteria
CLSA_COP	30 097	Canada	Provincial health registries and telephone sampling using random digit dialing of residents within 25–50 km of 1 of 11 data collection sites across seven Canadian provinces (Alberta, British Columbia, Manitoba, Nova Scotia, Newfoundland and Labrador, Ontario, Quebec)	In-depth interview in participants’ homes; physical and biological measurements at data collection sites	45–85 years old; able to give consent; excluding residents in the three territories, persons living on federal First Nations reserves and other First Nations settlements in the provinces, full-time members of the Canadian Armed Forces, and individuals living in institutions
CLSA_TRA	21 241	Canada	Canadian Community Health Survey (CCHS)—Healthy Aging cycle 4.2, provincial health registries and telephone sampling using random digit dialing across the 10 Canadian provinces	Telephone interview	45–85 years old; able to give consent; excluding residents in the three territories, persons living on federal First Nations reserves and other First Nations settlements in the provinces, full-time members of the Canadian Armed Forces, and individuals living in institutions
GLOBE	22 721	Netherlands	Municipal registries of the city of Eindhoven and 15 surrounding villages in the Southern part of the Netherlands	Postal questionnaire (baseline); in-depth interviews for two subsamples (random and chronically ill)	15–75 years old; non-institutionalised at baseline
HAPIEE_CZ	8857	Czech Republic	Population registers from Havirov/Karvina, Hradec Kralove, Jihlava, Kromeriz, Liberec and Usti nad Labem	Structured questionnaire at home; examination in clinic; face-to face computer-assisted personal interviewing (follow-up); death registers	45–69 years old
HAPIEE_LT	9360	Lithuania	Population registers from Kaunas	Structured questionnaire in clinic; examination in clinic; face-to face computer-assisted personal interviewing (follow-up); death registers	45–69 years old
HAPIEE_RU	7151	Russia	Population registers from Novosibirsk	Structured questionnaire in clinic; examination in clinic; face-to face computer-assisted personal interviewing (follow-up); death registers	45–69 years old
HUNT	106 429	Norway	Postal invitation to all citizens of Nord-Trøndelag County (24 municipalities)	Questionnaires and physical and biological measurements taken at health examination sites in each municipality	20+ years old
LASA1	3107	Netherlands	Municipal registries from three geographic regions: Amsterdam, Wormerland, Waterland (three municipalities in the West), Zwolle, Ommen, Genemuiden, Zwartsluis, Hasselt (North-East), and Oss, Uden, Boekel (South); oversampling of older people and older men in particular	Face-to-face interview; medical in-home interview; telephone interview	55–85 years old
LASA2	1837	Netherlands	Municipal registries from three geographic regions: Amsterdam, Wormerland, Waterland (three municipalities in the West), Zwolle, Ommen, Genemuiden, Zwartsluis, Hasselt (North-East), and Oss, Uden, Boekel (South); oversampling of older people and older men in particular	Face-to-face interview; medical in-home interview; telephone interview	55–65 years old
RECORD	9821	France	Invitation to all clinic patients at general health check-ups from four Centre d’Investigations Préventives et Cliniques (IPC) centers (Paris, Argenteuil, Trappes, Mantes-la-Jolie)	Questionnaires filled at health centres; physical and biological measurements during check-up	30–79 years old; residing in 1 of the112 preselected municipalities; able to answer questions themselves or with minimal help in French

*This represents the total number of unique participants, which includes sample boosting in follow-ups for some cohorts (GLOBE, HUNT, LASA1, RECORD).

CLSA_COP, Canadian Longitudinal Study on Aging (CLSA)^3^ comprehensive (in-depth); CLSA_TRA, CLSA tracking (telephone interview); GLOBE, Health and Living Conditions of the Population of Eindhoven and Surroundings (Gezondheid en Levens Omstandigheden Bevolking Eindhoven en omstreken)^4^; HAPIEE_CZ, The Health, Alcohol and Psychosocial Factors in Eastern Europe Study^5^—Czech Republic; HAPIEE_LT, HAPIEE—Lithuania; HAPIEE_RU, HAPIEE—Russia; HUNT, Nord-Trøndelag Health Study (Helseundersøkelsen i Nord-Trøndelag)^6^ 1–2–3 Cohort; LASA1, Longitudinal Aging Study Amsterdam (LASA)^7^ first cohort; LASA2, LASA second cohort; RECORD, Residential Environment and CORonary heart Disease Study.^8^

2. Define core variables and evaluate harmonisation potential

MINDMAP investigators collaboratively defined an initial targeted set of variables that they considered relevant for addressing selected research questions, through discussion and drawing on study teams’ expertise and the Maelstrom catalogue to identify available data. The harmonisation teams then used study documentation and data dictionaries to examine cohort-specific information collected in greater detail, and, in collaboration with Maelstrom Research, generated the final list of core variables (ie, the harmonised variables to be generated across studies) and their specifications (the DataSchema),^11^ which was adjusted from the initial list of target variables based on the available study-specific data. The DataSchema includes, for each variable, the name, definition, format (eg, integer, decimal) and units (eg, years, drinks/week), and rules for harmonisation were defined and documented. Rules for harmonisation refer to specifications for making decisions about whether and how particular variables are harmonised (eg, occurrence of angina must be diagnosed). Separate DataSchema variables were defined for baseline and each participant follow-up (data collection events). DataSchema variables targeting equivalent content at different time points were distinguished by the variable name suffix (eg, participant age was ‘sdc_age_0’ for baseline, ‘sdc_age_1’ for first follow-up, etc).

After finalising the DataSchema, the harmonisation teams assessed and documented the potential for each cohort-specific subpopulation to generate the DataSchema variables defined (ie, the harmonisation potential).^1 11^ Input from cohort teams (which included principal investigators, researchers and data managers, and could overlap with harmonisation team members) was regularly sought to address questions regarding missing metadata or unclear information. Harmonisation potential was considered ‘complete’ if cohort-specific variables were the same as the DataSchema or could be transformed to generate DataSchema variables. Harmonisation potential was deemed ‘impossible’ if relevant cohort-specific data were not collected or incompatible with DataSchema variable definitions. Variables were only retained in the DataSchema if they could be generated for two subpopulations or across two time points within a subpopulation. An example outlining harmonisation potential of cohort-specific variables and proposed algorithms to generate a DataSchema variable is provided in online supplemental table S1.

3. Process data under common format

Cohort-specific data required to generate the DataSchema variables were transferred to a central data server at Erasmus Medical Center, in accordance with consortium data-sharing policies.^2^ A central RStudio server allowed authenticated harmonisation team members to securely access and process cohort-specific data under the DataSchema format.^12^ The harmonisation teams assessed the quality of data provided for their domain of interest by checking univariate distributions and coherence among related variables (eg, skip patterns and consistency of participant responses among data collection events). Any questions were clarified with cohort study teams and documented. To explore representativeness of the populations, age and sex distributions at baseline were compared with national statistics from the same year for each subpopulation.

Harmonisation teams verified the harmonisation potentials attributed and, where relevant, developed algorithms (eg, online supplemental table S1) using R^13^ scripts to process cohort-specific data into the DataSchema format. Processing methods included direct mapping (target variable same as source variable), algorithmic transformation, calibration (converting units), rescaling and standardisation methods.^1^ In most domains, harmonisation was achieved predominantly through algorithmic transformations. More complex algorithms were required to account for longitudinal data in harmonising lifestyle and behavioural variables and health variables (eg, ‘Ever smoked’ or ‘Ever had a stroke’ in follow-ups used information from earlier data collection events). Mental well-being scores measured with different scales were harmonised by collapsing scores into quantiles or cases/non-cases based on cutoffs. Cognitive measures were harmonised using rescaling methods or by converting to z-scores.

4. Explore quality and content of harmonised data sets generated

Descriptive statistics were used to summarise final harmonisation statuses of DataSchema variables across cohort-specific data sets and scientific domains. First, to explore harmonised data content within each subpopulation, univariate distributions were generated for each DataSchema variable and reviewed for correct participant numbers, distributions and missing values, compared to the initial cohort-specific data provided. Harmonisation algorithms were validated for logic and script syntax, and correspondence of variable metadata with DataSchema specifications was verified. Outliers were retained but noted to inform researchers. Next, multivariate cross-checks were performed to validate coherence among related variables (eg, ‘current average number of cigarettes smoked per day’ only greater than 0 if ‘currently smokes any tobacco product’ is true). Finally, variability in participant distributions and missing values was examined across subpopulations. This helped identify additional corrections (eg, frequencies that were very different for one subpopulation revealed an error in cohort-specific coding) and variability across subpopulations to consider. Potential effects of factors such as cohort sampling and recruitment, data collection methods and harmonisation decisions on variable heterogeneity across subpopulations were explored (see online supplemental tables S4 and S5, figure S1) and will be part of ongoing discussions with researchers to improve utility of the harmonised data.

5. Preserve and disseminate harmonisation products

10.1136/jech-2020-214259.supp4 Supplementary data

A MINDMAP work repository was created on Github to document harmonisation decisions and processing scripts, and an interface was created on the Maelstrom Research catalogue (https://www.maelstrom-research.org/mica/network/mindmap) to collate and disseminate information about the cohort-specific designs and variables collected, DataSchema variables generated and harmonisation potential across studies. Secure access to the harmonised data for approved MINDMAP researchers is managed through the Opal data repository.

### Area-level environmental-exposure data

Information on social- and physical-environmental exposures came from publicly available resources and were linked to cohort participant data using residential locations of cohort participants. To protect participant privacy, a series of steps were taken to blind the geospatial information, and linked data including only participant ID and environmental-exposure data (without any geospatial information) were transferred to the MINDMAP central server. More details on the sources of information and procedures used to link data are available elsewhere.^2 14 15^

**Figure 1:**
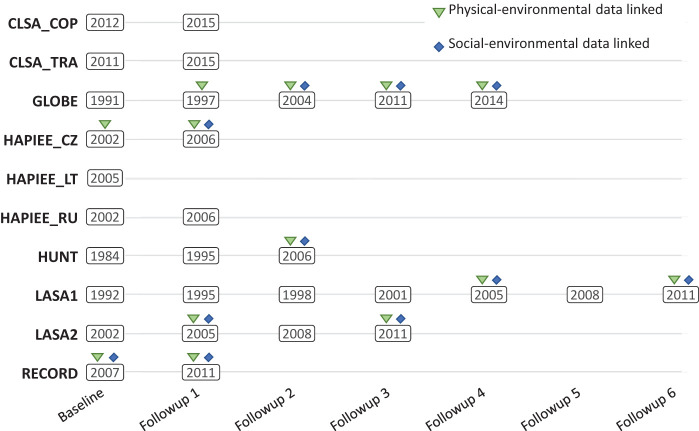
Overview of start years of data collection events in cohort studies and of time points with linked area-level information. Note that these do not reflect the time span of each data collection event.

Availability of area-level data was limited, and years of collection did not necessarily correspond to the timing of the cohorts-specific data collection events (figure 1). Area-level data were thus linked using information collected at the closest year to each data collection event. Harmonisation of the environmental-exposure DataSchema variables followed the approach used for the individual-level cohort data. Processing of physical-environmental data and social-environmental variables to a common format included direct mapping and more complex transformations (eg, dichotomised variable of average income of area residents below/above country-specific household mean).

## RESULTS

### Cohort-specific data

**Figure 2:**
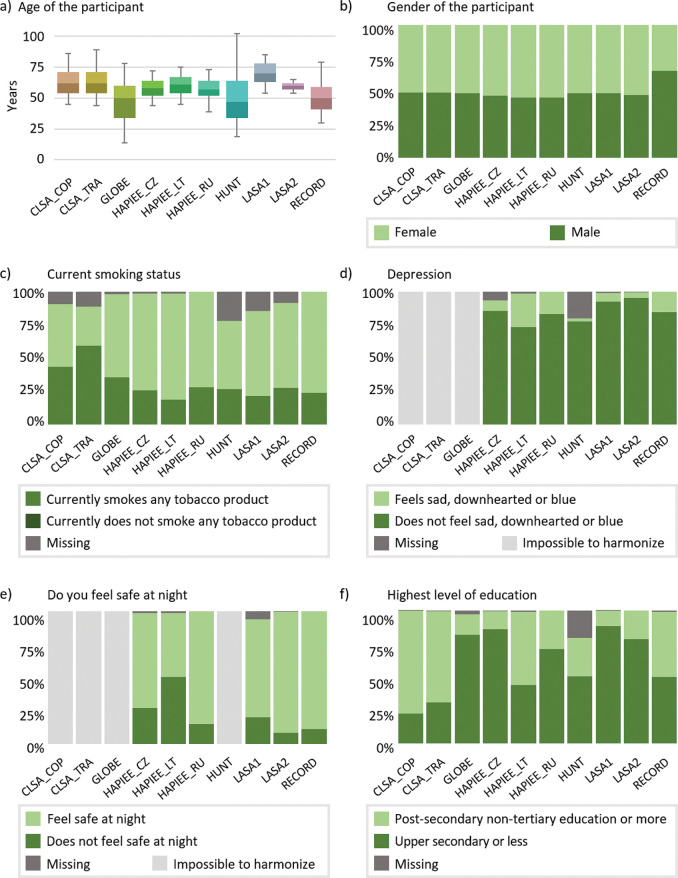
Participant distributions at baseline for selected harmonised variables. ‘Impossible to harmonise’ indicates variables that could not be harmonised for a subpopulation data set. ‘Missing’ indicates missing values within a subpopulation data set for variables with complete harmonisation status.

Cohort baseline data collection years ranged from 1984 to 2012, and the number of data collection events ranged from 1 to 7 (figure 1). The number of participants in each of the 10 cohort subpopulations ranged from 1837 to 106 429, with a total of 220 621 participants (table 1). Participant sex distributions (overall 50.7% female, 49.3% male) were generally similar to contemporary age-matched national populations, with the exception of Residential Environment and CORonary heart Disease Study (RECORD), which recruited a larger proportion of males relative to the general French population (34.5% female, 65.5% male at baseline) (table 2, figure 2). The participants median age at baseline was 56 years (range 14–102 years). Age distributions at baseline varied widely across sampled subpopulations, frequently differing from contemporary national populations (table 2).

**Table 2:** Baseline sex and age distributions in the MINDMAP subpopulations and in contemporaneous national populations

	CLSA_COP		CLSA_TRA		GLOBE		HAPIEE_CZ		HAPIEE_LT		HAPIEE_RU		HUNT		LASA1		LASA2		RECORD	
Recruited ages (years)	45–85		45–85		15–75		45–69		45–69		45–69		20+		55–85		55–65		30–79	
Baseline year	2012		2011		1991		2002		2005		2002		1984		1992		2002		2007	
	Cohort	Canada	Cohort	Canada	Cohort	Netherlands	Cohort	Czech Republic	Cohort	Lithuania	Cohort	Russia	Cohort	Norway	Cohort	Netherlands	Cohort	Netherlands	Cohort	France
Sex																				
ߓFemale	50.9	51.5	51.0	53.4	51.5	49.9	53.4	51.9	54.6	55.9	54.4	53.4	51.0	50.9	51.5	55.5	52.6	53.7	34.5	51.6
ߓMale	49.1	48.5	49.0	46.6	48.5	50.1	46.6	48.1	45.4	44.1	45.6	46.6	49.0	49.1	48.5	44.6	47.4	46.3	65.5	48.4
Ages (years)																				
15–24	–	–	–	–	11.7	19.2	–	–	–	–	–	–	7.2*	10.5	–	–	–	–	–	–
25–34	–	–	–	–	13.2	22.1	–	–	–	–	–	–	19.4	20.8	–	–	–	–	9.9*	11.5
35–44	–	–	–	–	12.5	20.1	0.5	–	–	–	0.3	–	19.6	18.9	–	–	–	–	25.6	24.6
45–54	25.2	37.2	27.4	37.7	24.3	16.0	35.8	49.9	25.2	46.9	36.1	52.4	13.8	13.2	0.1	–	0.5	–	27.2	23.7
55–64	32.8	31.1	30.9	31.1	22.3	12.2	41.7	36.8	39.5	36.2	40.4	32.2	16.3	14.9	31.1	43.8	99.4	92.7	25.5	20.2
65–74	24.5	19.6	21.8	18.9	15.7	9.7	22.0*	13.4	35.3*	17.0	23.3*	15.3	14.4	12.7	31.2	35.1	0.1*	7.3	9.6	13.9
75–84	16.9	11.3	18.8	11.5	0.1*	0.7	–	–	–	–	–	–	7.6	7.2	36.5	20.0	–	–	2.2*	6.2
≥85	0.6*	0.8	1.1*	0.8	–	–	–	–	–	–	–	–	1.7	1.9	1.2*	1.1	–	–	–	–

*Cohort age range limits fall within this age bin.

National statistics were drawn from the United Nations Statistics Division’s Demographic Statistics Database (http://data.un.org/Data.aspx?d=POP&f=tableCode%3A22), which compiles data from questionnaires dispatched annually to national statistical offices. Distributions are calculated for national data restricted to the same age ranges represented in the cohorts.

Percentages may not add up to 100% due to rounding.

CLSA_COP, Canadian Longitudinal Study on Aging (CLSA) comprehensive (in-depth); CLSA_TRA, CLSA tracking (telephone interview); GLOBE, Health and Living Conditions of the Population of Eindhoven and Surroundings (Gezondheid en Levens Omstandigheden Bevolking Eindhoven en omstreken); HAPIEE_CZ, The Health, Alcohol and Psychosocial Factors in Eastern Europe Study—Czech Republic; HAPIEE_LT, HAPIEE —Lithuania; HAPIEE_RU, HAPIEE —Russia; HUNT, Nord-Trøndelag Health Study (Helseundersøkelsen i Nord-Trønelag) 1–2–3 Cohort; LASA1, Longitudinal Aging Study Amsterdam (LASA) first cohort; LASA2, LASA second cohort; RECORD, Residential Environment and CORonary heart Disease Study.

### DataSchema variables and harmonisation potential

The DataSchema (v2.0) included a total of 2841 variables: 993 from individual-level determinants and outcome data; 1848 from environmental-exposure data. As individual-level determinants and outcome variables were only defined for data collection events if they could be generated for more than one subpopulation or more than one data collection event within subpopulations, numbers differed across time points. There were 166 individual-level determinants and outcome variables at baseline and 165, 159, 139, 134, 112 and 105 at follow-ups 1, 2, 3, 4, 5 and 6, respectively. For environmental-exposure variables, 41 social-environmental and 223 physical-environmental (264 total) variables were defined for each time point. Online supplemental table S2 provides DataSchema information for baseline variables and administrative variables (data collection years and time intervals). Table 3 provides the distribution of DataSchema variables by domain and subdomain of information, following Maelstrom Research’s cataloguing classification.^10^

**Table 3:** Distribution of DataSchema variables and average per cent complete harmonisation potential by domains of information

Domain of information (N DataSchema variables per subdomain)	N DataSchema variables (% total)	% Complete statuses
Sociodemographic and economic characteristics	120 (4.2)	64.1
Age/birthdate (7), sex/gender (7), marital status (14), family and household structure (20), education (10), residence (28), labor force and retirement (17), income, possessions and benefits (17)		
Lifestyle and behaviours	190 (6.7)	53.7
Tobacco (45), alcohol (37), nutrition (16), physical activity (73), sleep (12) and leisure activities (7)		
Perception of health, quality of life, development and functional limitations	55 (1.9)	71.4
Perception of health (21), quality of life (24) and functional limitations (10)		
Diseases, ICD-10	31 (1.1)	68.0
Circulatory system disease (19), endocrine, nutritional and metabolic diseases (12)		
Medication and supplements	15 (0.5)	45.7
Medication and supplement intake (15)		
Physical measures and assessments	91 (3.2)	73.3
Anthropometry (91)		
Life events, life plans, beliefs and values	33 (1.2)	26.4
Life events (33)		
Cognition, personality and psychological measures and assessments	171 (6.0)	52.7
Cognitive functioning (84), psychological distress and emotions (81), other psychological measures and assessments (6)		
Social environment and relationships	523 (18.4)	42.1
Social network (42), social participation (110), social support (50) and other social environment characteristics (321)		
Physical environment	1599 (56.3)	78.6
Housing characteristics (7), built environment/neighborhood characteristics (1592)		
Administrative information	13 (0.5)	100.0
Date and time (13)		
Total	2841 (100)	

ICD-10, International Statistical Classification of Diseases and Related Health Problems, 10th Revision.

10.1136/jech-2020-214259.supp2 Supplementary data

The 10 subpopulations differed in the number of data collection events (from 1 to 7) and in the number of time points with linked area-level environmental-exposure data (from 0 to 4). This resulted in a total of 30 cohort-specific data collection events, 13 of these with linked area-level data on social- and/or physical-environmental exposures (figure 1). Harmonisation potentials of DataSchema variables were evaluated only where applicable, that is, where the cohort collected data for that time point (eg, not considering sdc_age_6 for subpopulations without six follow-up events) and, for environmental-exposure variables, if area-level data were linked. This resulted in 8165 harmonisation potentials to evaluate. The overall percentage of complete harmonisation statuses was 63.0% (5144 complete statuses/8165 evaluated). Individual determinants and outcome variables accounted for 4733 harmonisation statuses, of which 2523 (53.3%) were complete and 2210 (46.7%) were impossible. Environmental-exposure variables accounted for 3432 statuses, of which 2621 (76.4%) were complete and 811 (23.6%) were impossible. Harmonisation potential also varied across domains of information, ranging from 26.4% in life events, beliefs and values to 73.3% in physical measures and 78.6% in physical environment (table 3). Finally, harmonisation potential varied across subpopulations, ranging from 34.7% in HUNT to 80.6% in LASA1. All harmonisation statuses are presented in online supplemental table S3 and are also available on the Maelstrom catalogue.^16^ Complete statuses reflect harmonised variables achieved with processing methods ranging from direct mapping to complex algorithms using information and conditions from many cohort-specific variables, and the complexity of harmonisation algorithms required and any important decisions taken in harmonising each variable are available in RMarkdown files.

10.1136/jech-2020-214259.supp3 Supplementary data

### Harmonised data content

Participant distributions and missing values varied across the 10 subpopulations. For example, figure 2 presents participant distributions at baseline for several variables, including feeling depressed, feeling safe at night, current smoking status and highest level of education. These illustrate variability in harmonisation potential, distribution of participants across categories, and percentage of missing data. Current smoking status and highest level of education could be created for all subpopulations, while depression was impossible for three subpopulations and feeling safe at night was impossible to generate for four. The percentage of participants with secondary-level education or more ranged from 11.4% in LASA1 to 77.6% in CLSA_COP, and the percentage of missing data ranged from 0.2% in CLSA_COP to 42.5% in HUNT. Note that missing values in harmonised data can result from missing values in cohort-specific data or harmonisation processing (eg, cohort-specific values of ‘Don’t know’ and ‘Prefer not to answer’ were coded to missing in harmonised variables).

Various factors can explain the observed subpopulation variability including, but not limited to the population sampling frame, recruitment procedures, data collection methods and question format. An example of exploring the potential influence of such factors using a cognitive functioning variable is provided in online supplemental tables S4 and S5, figure S1.

### Documentation of harmonised data sets

Information about the MINDMAP project, participating cohort designs and harmonisation potential is available on the Maelstrom Research catalogue.^16^ The web interface includes the capacity to search cohort-specific and DataSchema variables and documents data harmonisation potential across cohort subpopulations. In addition, harmonisation outputs and annotated R processing scripts are available on the MINDMAP GitHub repository.^17^ As documentation is updated regularly to reference the most recent version of harmonised data sets, current online information will vary from the information reported in this article.

## DISCUSSION

MINDMAP implemented a rigorous multinational collaborative process to generate a large harmonised data set, which serves as a valuable resource for research on urban environments and mental health in ageing adults. The current harmonised data set (v2.0) includes 2841 harmonised variables from 30 data collection events across six cohort studies (including 10 subpopulations). The breadth of information, diversity of cohort studies and availability of longitudinal and environmental data are important strengths of the project. The success of the harmonisation process depended on the collaborative work of several international research teams contributing methodological and content expertise (cognition, environmental exposure, etc). This collaboration was enacted through a rigorous methodological approach and close communication among the domain experts, Maelstrom Research team and cohort study teams. In parallel, the technological infrastructure implemented allowed the international teams to work remotely on a central server to harmonise data, while protecting participant privacy, and now provides investigators with an efficient means to readily access and analyse the harmonised data set.^2^

The MINDMAP-harmonised data supports exploration of the impact of social and physical environments from 10 subpopulations in seven countries, which is quite novel and critical for studying the influence of urban environment on healthy ageing. Including diverse urban populations provides a wide range of exposures to inform how structural differences between countries or cities influence mental well-being and health. While not all core variables could be created across all cohort-specific data sets (the global harmonisation potential was 62.8%), the data set generated supports valuable subanalysis across selected variables and/or studies (eg, analyses to date by JM Noordzij *et al* 2020, M Ruiz *et al* 2020, EJ Timmermans *et al* 2020). However, the utility for each research question needs to be carefully examined, and it is essential to recognise the potential and limitations of the data used. Researchers using the harmonised data set should consider multiple potential sources of subpopulation variability, where relevant, for their specific research needs. For example, where data collection methods and harmonisation processing vary among cohorts, heterogeneity in variable distributions could reflect a combination of underlying subpopulation differences and methodology, which could affect decisions such as selecting data to analyse, choosing an analytical approach and interpreting subpopulation heterogeneity.

The harmonised data generated presents important limitations. Definition of the target variables required a balance between ensuring integrity of scientific content (being as homogenous as possible across studies) and the need to allow a certain level of heterogeneity (combining information collected in different formats).^18^ These trade-offs were apparent, for example, in mental well-being and cognitive performance variables. Choosing to harmonise only information from identical scales provides more homogeneity but, as cohorts rarely use common scales, results in including fewer studies. On the other hand, using methods that increase the potential to integrate information across cohorts (eg, applying cut-offs, standardisation models) generally results in loss of information, increased heterogeneity and reduced ability to examine certain population differences.^19 20^ Creation of the initial DataSchema (v2.0) attempted to find this balance, but further exploration of the data content remains essential to better understand the quality of the variables generated.^21–24^ This will entail updates of the harmonised data set, optimising data content to better support current and upcoming research needs.

Several additional factors should be considered by investigators aiming to understand and use MINDMAP data. These factors include, but are not limited to, the following. First, subpopulation backgrounds, sampling frames, recruitment procedures and data collection profiles are, as expected, different. Second, subpopulations differ in their representativeness of national populations. Summaries in the current article come from raw sample data, but researchers should consider adjustments for analysis and inference about underlying populations. For example, CLSA data were designed to be analysed as one cohort (rather than the two samples presented here for harmonisation purposes) and with sampling weights (inflation weights for descriptive analysis and analytic weights for statistical testing). Third, the quality and variable resolution of the cohort-specific data provided varied. For example, outliers were noted but left in the data set, and the impact of missing values was not examined. Fourth, the number and timing of data collection events varied across cohorts. Longitudinal data offers important advantages over cross-sectional analyses but introduces other complexities to be considered during analyses.^25^ Interpretation of results must be made in consideration of such factors.

The MINDMAP process followed Maelstrom Research guidelines for rigorous retrospective data harmonisation,^1 9–11^ which have also been used by other retrospective harmonisation endeavours across population-based studies.^26–29^ The approach ensured generation of comprehensive and searchable documentation, including (1) cohort-specific designs and variables collected; (2) definition and characteristics of the DataSchema variables; (3) harmonisation potential across studies; and (4) algorithms used to process cohort-specific data into DataSchema variables. We hope that the information provided will help to properly understand, optimally use and further develop the MINDMAP data set.

## CONCLUSION

The MINDMAP team implemented a collaborative and transparent process to generate a valuable harmonised data set to be used for research on ageing and mental well-being across different country and urban contexts. The current article describes the harmonisation process and harmonised data generated. More broadly, it provides an example of how large multinational collaborations can successfully implement and document retrospective harmonisation to generate valuable epidemiological data sets.

**What is already known on this subject**

- Retrospective data harmonisation is important for achieving or improving comparability of similar data items collected by different studies. It enables leveraging existing cohort data resources to address research questions that are difficult or impossible to address in single studies and has become an important tool in collaborative research initiatives.
- Harmonisation of existing data raises major challenges, and transparent and thorough documentation of the harmonisation process is required for researchers to understand and use harmonised data.

**What this study adds**

- The MINDMAP team harmonised data from six international cohort studies to examine the individual and environmental determinants of mental well-being in older adults in diverse urban populations.
- This article describes the collaborative harmonisation process implemented and serves to inform researchers on how large multinational collaborations can successfully implement and document retrospective harmonisation to generate valuable epidemiological data sets.
