# Supplementary material for: Overview of retrospective data harmonisation in the MINDMAP project: process and results
Source: J Epidemiol Community Health. 2020 Nov 10;75(5):433–41. doi: 10.1136/jech-2020-214259 (PMC8053335; doi:10.1136/jech-2020-214259)
Supplement: Supplementary file 1 [file jech-2020-214259supp001.pdf]

Online supplementary material: Example of considerations in using MINDMAP harmonized data

An illustrative example is presented for examining a MINDMAP DataSchema variable generated and potential sources of heterogeneity to consider. If a researcher were interested in examining cognitive functioning, a candidate measure of interest might be the variable cog_imm_rscale_0, immediate word recall measured at baseline (Table S4).

Table S4. DataSchema specifications for the DataSchema variable cog_imm_rscale_0.

Variable name	Harmonization domain	Variable label	Variable description	Value type	Variable units
cog_imm_rscale_0	Mental health outcomes	Immediate word recall	Rescaled value (ranging from 0 to 10) of the total number of words immediately recalled during a test of verbal memory. If the test is performed more than once, data are based on the first trial.	Integer	Words

Of the 6 studies and 10 subpopulations represented in the MINDMAP harmonized dataset v2.0, harmonization of the variable cog_imm_rscale_0 was complete for 3 studies (CLSA, HAPIEE, LASA) and 7 subpopulations. The raw distribution of the harmonized variable across the subpopulations is presented in Figure S1, and a few notable aspects that differed among these 3 studies are summarized in Table S5. More details on differences in cohort design among subpopulations can be found in Table 1 in the main text.

Figure S1. Distributions across subpopulations for that harmonized variables cog_proc_std_0 and cog_imm_rscale_0.

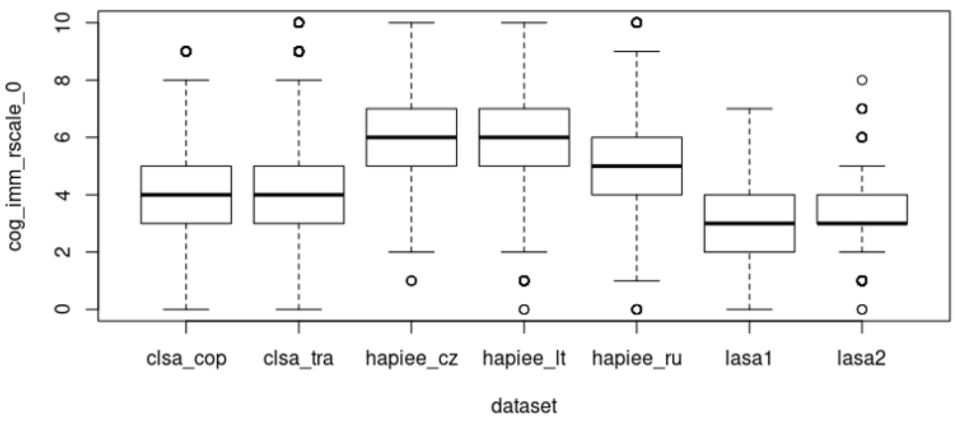

Table S5. Some study-specific differences in studies that could generate the harmonized variable cog_imm_rscale_0.

Study	Subpopulations	Country	Baseline years	Age ranges	Comment on harmonization processing to generate cog_imm_rscale_0

CLSA	2	Canada	2011, 2012	45-85 years old	Immediate word recall of 15 words from Rey's Auditory Verbal Learning Test was performed in CLSA. Only one trial of immediate word recall was assessed. The number of words immediately recalled were rescaled from 0-15 to 0-10 in CLSA.
HAPIEE	3	Czech Republic, Lithuania, Russia	2002, 2005	45-69 years old	Immediate word recall of 10 common two syllable words was measured in HAPIEE. As three trials of immediate word recall were assessed, data from the first trial were used. The number of words (0-10) immediately recalled were used as provided by the HAPIEE study.
LASA	2	The Netherlands	1992, 2002	55-85 years old, 55-65 years old	Immediate word recall of 15 words from Rey's Auditory Verbal Learning Test was performed in LASA. As three trials of immediate word recall were assessed, data from the first trial were used. The number of words immediately recalled were rescaled from 0-15 to 0-10 in LASA.

Visual inspection of the distributions in Figure S1 suggests there are population differences in mean values and variance in the variable, and the information in Table S5 indicates that studies differed in key dimensions including source countries, baseline data collection years, age ranges included, and measurement of study-specific variables used to generate the harmonized variable. The differences in study-specific variable resulted in differences in harmonization processing algorithms, with direct mapping for HAPIEE and rescaling from a 15-word scale to a 10-word range for CLSA and LASA. Hence the variability in distributions in Figure S1 could potentially be affected by differences cohort-specific design or methodology and harmonization processing, as well as underlying population differences of possible research interest.

How a researcher might design their analysis and account for study-specific heterogeneity will depend on the specific research aims but could include the following. The researcher might consider if the harmonization decisions are acceptable for the research needs or if it would be preferable to restrict analyses to studies that used the same original test. This could require exploratory statistics to examine if and how the measurement test affects the observed values of the harmonized variable. If the researcher proceeds to select and analyze the variable, they might consider if and how to render the populations more comparable, possibly by restricting comparisons to similar age ranges or data collection years. In pooled analyses of general trends across populations, these factors could be included as confounders to aid in interpretation. Whether analyses are pooled or not, interpretations of differences among populations should be made in consideration of study-specific differences and potential limitations introduced by them. In some cases, differences among populations may not be attributable to one specific source. For example, if countries differed in values of cog_imm_rscale_0, it might be difficult to definitively disentangle differences due to study design from differences due to other country-level factors without more information.

In addition to the study-specific factors listed above, MINDMAP researchers will need to consider cohort design aspects such as (but not limited to) sampling frame, recruitment, data collection mode, and exclusion criteria that could affect the representativeness and comparability of the sampled subpopulations. In pooled analyses, researchers may need to consider aspects such as sampling weights and the hierarchical structure of the data, with subpopulation observations nested within studies, and account for this in statistical models where relevant. For example, CLSA was designed to be a representative national sample, with the two subpopulations meant to

be treated as one cohort, and with specified sampling weights to be applied for both descriptive and inferential statistics, while other studies do not have these features.

The factors discussed here are just a subset of possible considerations and meant to suggest some key aspects of the MINDMAP harmonized variables to consider. Specific considerations will vary on a case-by-case basis depending on variables of interest, but researchers will need to carefully examine study and variable documentation to understand the dataset and make decisions for their specific analyses in all cases.
