# Supplementary material for: Overview of retrospective data harmonisation in the MINDMAP project: process and results
Source: J Epidemiol Community Health. 2020 Nov 10;75(5):433–41. doi: 10.1136/jech-2020-214259 (PMC8053335; doi:10.1136/jech-2020-214259)
Supplement: Supplementary data [file jech-2020-214259supp004.pdf]

Table S3. Harmonization statuses (v2.0, April 2020) presented by data collection event within subpopulations. Note that, except for Administrative Information, the Variable Name indicates the root name of equivalent variables that could be created at different time points, rather than DataSchema variable names. Blank cells indicate instances that were not applicable, i.e., where an equivalent variable was not created for a data collection evet or where no area-level information was linked. Summary statistics are presented for harmonization potential across variables and data collection events. These statistics exclude instances where DataSchema variables were not applicable to a given subpopulation.

● individual-level determinants and outcomes ✓ complete
○ area-level environmental exposures ✗ incomplete

SUBPOPULATION / DATA COLLECTION EVENT

		CLSA_COP		CLSA_TRA		GLOBE					HAPIEE_CZ		HAPIEE_LT	HAPIEE_RU		HUNT			LASA1							LASA2				RECORD		Percent
**DataSchema variable root**		BL	F1	BL	F1	BL	F1	F2	F3	F4	BL	F1	BL	BL	F1	BL	F1	F2	BL	F1	F2	F3	F4	F5	F6	BL	F1	F2	F3	BL	F1	Complete
**Sociodemographic and economic characteristics**																																
sdc_age	●	✓	✓	✓	✓	✓	✓	✓	✓	✓	✓	✓	✓	✓	✓	✓	✓	✓	✓	✓	✓	✓	✓	✓	✓	✓	✓	✓	✓	✓	✓	100.0
sdc_gender	●	✓	✓	✓	✓	✓	✓	✓	✓	✓	✓	✓	✓	✓	✓	✓	✓	✓	✓	✓	✓	✓	✓	✓	✓	✓	✓	✓	✓	✓	✓	100.0
sdc_employ	●	✓	✓	✓	✓	✓	✓	✓	✓	✓	✓	✓	✓	✓	✓	✓	✗	✓	✓	✓	✓	✓	✓	✓	✓	✓	✓	✓	✓	✓	✗	93.3
sdc_retire	●	✓	✓	✓	✓	✓	✓	✓	✓	✓	✓	✓	✓	✓	✓	✗	✗	✗	✓	✓	✓	✓	✓	✓	✓	✓	✓	✓	✓	✓	✓	90.0
sdc_retire_age	●	✓	✓	✓	✓	✗	✗	✗			✓	✗	✗	✓	✗	✗	✗	✗	✗	✗	✗					✗	✗	✗		✗	✗	26.1
sdc_highest_edu	●	✓	✗	✓	✗	✓	✗	✓	✓	✓	✗	✗	✗	✗	✗	✓	✓	✗	✓	✓	✓	✓	✓			✓	✓	✓	✓	✓	✓	67.9
sdc_postsec_edu	●	✓	✗	✓	✗	✓	✗	✓	✓	✓	✓	✗	✓	✓	✗	✓	✓	✗	✓	✓	✓	✓	✓			✓	✓	✓	✓	✓	✓	78.6
sdc_income	●	✗	✗	✗	✗	✓	✓	✓	✓	✓	✗	✗	✗	✗	✗	✗	✗	✗	✓	✓	✓	✓	✓	✓	✓	✓	✓	✓	✓	✓	✓	60.0
sdc_civstat	●	✓	✓	✓	✓	✓	✓	✓	✓	✓	✓	✗	✓	✓	✗	✓	✓	✓	✓	✓	✓	✓	✓	✓	✓	✓	✓	✓	✓	✗	✗	86.7
sdc_livstat	●	✓	✓	✓	✓	✓	✓	✓	✓	✓	✓	✗	✓	✓	✗	✓	✓	✓	✓	✓	✓	✓	✓	✓	✓	✓	✓	✓	✓	✓	✓	93.3
sdc_num_pplhh	●	✓	✓	✓	✓	✗	✗	✓	✓	✓	✓	✓	✓	✓	✓	✗	✓	✗	✗	✗	✗	✗	✗			✗	✗	✗	✗	✓	✓	53.6
sdc_num_childhh	●	✓	✓	✓	✓	✗	✗	✗	✗	✗	✓	✓	✓	✓	✓	✗	✗	✗	✗	✗	✗	✗	✗			✗	✗	✗	✗	✓	✓	39.3
sdc_num_child	●	✗	✗	✗	✗	✗	✗	✗	✗	✗	✗	✗	✓	✗	✗	✗	✓	✗	✗	✗	✗	✗	✗			✗	✗	✗	✗	✓	✓	21.4
sdc_child	●	✗	✗	✗	✗	✓	✗	✗	✗	✓	✗	✗	✓	✗	✗	✗	✓	✗	✗	✗	✗	✗	✗			✗	✗	✗	✗	✓	✓	63.3
soc_res_num_yrsnbh	●	✓	✓	✓	✓	✗	✗	✓	✓	✓	✗	✗	✗	✗	✗	✗	✗	✗	✓	✓	✓	✓	✓	✓	✓	✓	✓	✓	✓	✓	✗	63.3
soc_res_num_yrshome	●	✓	✓	✓	✓	✗	✗	✗	✗	✗	✗	✗	✗	✗	✗	✗	✗	✗	✓	✓	✓	✓	✓	✓	✓	✓	✓	✓	✓	✓	✗	53.3
soc_res_move	●	✗	✗	✗	✗	✓	✓	✓	✗	✗	✗	✗	✗	✗	✗	✗	✗	✗	✓	✓	✓	✓	✓	✓	✓	✗	✓	✓	✓	✗	✗	43.3
soc_res_indep	●	✓	✓	✓	✓	✓	✗	✓	✗	✗	✗	✗	✗	✗	✗	✗	✗	✗	✓	✓	✓	✓	✓	✓	✓	✓	✓	✓	✓	✗	✗	56.7
soc_res_home_own_dich	●	✓	✓	✓	✓	✓	✓	✓	✓	✓	✗	✗	✗	✗	✗	✗	✗	✗	✓	✓	✓	✓	✓	✓	✓	✓	✓	✓	✓	✓	✓	73.3
soc_res_home_own	●	✓	✓	✓	✓	✗	✗	✗			✗	✗	✗	✗	✗	✗	✗	✗	✓	✓	✓					✓	✓	✓		✗	✗	43.5
**Lifestyle and behaviors**																																
lsb_alc_cur	●	✓	✓	✓	✓	✓	✓	✗	✓	✓	✓	✓	✓	✓	✓	✓	✓	✓	✓	✓	✓	✓	✓	✓	✓	✓	✓	✓	✓	✓	✓	96.7
lsb_alc_ever	●	✓	✓	✓	✓	✗	✓	✗	✗	✗	✗	✗	✗	✗	✗	✓	✗	✓	✓	✓	✓	✓	✓	✓	✓	✓	✓	✓	✓	✓	✓	66.7
lsb_alc_binge	●	✓	✓	✓	✓	✓	✓	✗	✓	✗	✓	✓	✓	✓	✓	✗	✗	✗	✓	✓	✓	✓	✓	✓	✓	✓	✓	✓	✓	✗	✗	76.7
lsb_alc_total	●	✓	✓	✓	✓	✗	✗	✗	✗		✓	✓	✓	✓	✓	✗	✓	✓	✗	✗	✗	✗				✗	✗	✗	✗	✓	✗	46.2
lsb_alc_wine	●	✓	✓	✓	✓	✗	✗	✗	✗		✓	✓	✓	✓	✓	✗	✓	✓	✗	✗	✗	✗				✗	✗	✗	✗	✓	✗	50.0
lsb_alc_beer	●	✓	✓	✓	✓	✗	✗	✗	✗		✓	✓	✓	✓	✓	✗	✓	✓	✗	✗	✗	✗				✗	✗	✗	✗	✓	✗	50.0
lsb_alc_liq	●	✓	✓	✓	✓	✗	✗	✗	✗		✓	✓	✓	✓	✓	✗	✓	✓	✗	✗	✗	✗				✗	✗	✗	✗	✗	✗	42.3
lsb_smk_cur	●	✓	✓	✓	✓	✓	✓	✓	✓	✓	✓	✓	✓	✓	✓	✓	✓	✓	✓	✓	✓	✓	✓	✓	✓	✓	✓	✓	✓	✓	✓	100.0
lsb_smk_status	●	✓	✓	✓	✗	✓	✓	✓	✓	✓	✓	✓	✓	✓	✓	✓	✓	✓	✓	✓	✓	✓	✓	✓	✓	✓	✓	✓	✓	✓	✓	96.7
lsb_smk_ever	●	✓	✓	✓	✗	✓	✓	✓	✓	✓	✓	✓	✓	✓	✓	✓	✓	✓	✓	✓	✓	✓	✓	✓	✓	✓	✓	✓	✓	✓	✓	96.7
lsb_cig_qty	●	✗	✗	✗	✗	✓	✓	✓	✓	✓	✓	✓	✓	✓	✓	✓	✓	✓	✓	✓	✓	✓	✓	✓	✓	✓	✓	✓	✓	✓	✓	86.7
lsb_smk_start	●	✓	✗	✓	✗	✓	✗	✓	✗	✗	✓	✗	✓	✓	✗	✓	✓	✓	✓	✓	✓	✓	✓	✓	✓	✓	✓	✓	✓	✗	✗	70.0
lsb_smk_stop	●	✗	✗	✗	✗	✗	✓	✗	✗	✗	✓	✓	✓	✓	✓	✗	✓	✓	✓	✓	✓	✓	✓	✓	✓	✓	✓	✓	✓	✗	✗	63.3
lsb_smk_py	●	✗	✗	✗	✗	✓	✓	✓			✓	✗	✓	✓	✗	✓	✓	✓	✗	✗	✗					✓	✓	✓		✓	✗	56.5
lsb_pa_walk_freq	●	✓	✓	✓	✓	✗	✗	✗	✗	✗	✗	✗	✗	✗	✗	✗	✗	✗	✗	✗	✗	✗	✗	✗	✗	✗	✗	✗	✗	✗	✗	13.3
lsb_pa_walk_time	●	✓	✓	✓	✓	✗	✗	✗	✗	✗	✗	✗	✗	✗	✗	✗	✗	✗	✓	✓	✓	✓	✓	✓	✓	✓	✓	✓	✓	✓	✗	56.7
lsb_pa_walk_trans	●	✗	✗	✗	✗	✗	✗	✓	✗	✓	✗	✗	✗	✗	✗	✗	✗	✗	✗	✗	✗	✗	✗			✗	✗	✗	✗	✓	✗	10.7
lsb_pa_walk_leis	●	✗	✗	✗	✗	✗	✗	✓	✓	✓	✗	✗	✗	✗	✗	✗	✗	✗	✗	✗	✗	✗	✗			✗	✗	✗	✗	✓	✗	14.3
lsb_pa_cycle	●	✗	✗	✗	✗	✗	✗	✓	✓	✓	✗	✗	✗	✗	✗	✗	✗	✗	✓	✓	✓	✓	✓	✓	✓	✓	✓	✓	✓	✓	✗	50.0
lsb_pa_garden	●	✗	✗	✗	✗	✗	✗	✓	✓	✓	✗	✗	✗	✗	✗	✗	✗	✗	✓	✓	✓	✓	✓	✓	✓	✓	✓	✓	✓	✓	✗	50.0
lsb_pa_garden_time	●	✗	✗	✗	✗	✗	✗	✓	✓	✓	✗	✗	✗	✗	✗	✗	✗	✗	✓	✓	✓	✓	✓	✓	✓	✓	✓	✓	✓	✓	✗	50.0
lsb_pa_house	●	✓	✓	✓	✓	✗	✗	✓	✓	✓	✗	✗	✗	✗	✗	✗	✗	✗	✓	✓	✓	✓	✓	✓	✓	✓	✓	✓	✓	✓	✗	63.3
lsb_pa_house_time	●	✗	✗	✗	✗	✗	✗	✓	✓	✓	✗	✗	✗	✗	✗	✗	✗	✗	✓	✓	✓	✓	✓	✓	✓	✓	✓	✓	✓	✓	✗	50.0
lsb_pa_sport	●	✓	✓	✓	✓	✓	✓	✓	✗	✓	✓	✗	✗	✓	✗	✓	✗	✓	✓	✓	✓	✓	✓	✓	✓	✓	✓	✓	✓	✓	✗	83.3
lsb_pa_sport_time	●	✓	✓	✓	✓	✓	✓	✓	✓	✓	✓	✗	✗	✓	✗	✗	✗	✗	✗	✗	✗	✗	✗	✗	✗	✗	✗	✗	✗	✗	✗	36.7
lsb_veg	●	✓	✓	✗	✗	✗	✗	✗			✗	✗	✗	✗	✗	✗	✗	✓	✗	✗	✗					✗	✗	✗		✓	✓	21.7
lsb_fruit	●	✓	✓	✗	✗	✗	✗	✗			✗	✗	✗	✗	✗	✗	✗	✓	✗	✗	✗					✗	✗	✗		✓	✓	21.7
lsb_water	●	✗	✗	✗	✗	✗	✗	✗	✗	✓	✗	✗	✗	✗	✗	✗	✗	✓	✗	✗	✗	✗	✗			✗	✗	✗	✗	✓	✓	14.3

DataSchema variable root	CLSA_COP		CLSA_TRA		GLOBE					HAPIEE_CZ		HAPIEE_LT	HAPIEE_RU		HUNT			LASA1							LASA2				RECORD		Percent
	BL	F1	BL	F1	BL	F1	F2	F3	F4	BL	F1	BL	BL	F1	BL	F1	F2	BL	F1	F2	F3	F4	F5	F6	BL	F1	F2	F3	BL	F1	Complete
lsb_coffee_qty	✗	✓	✓	✓	✓	✗	✗	✗	✗	✗	✗	✗	✗	✗	✗	✓	✓	✗	✗	✗	✗	✗			✗	✗	✗	✗	✗	✗	21.4
lsb_sleep_hours	✗	✗	✗	✗	✗	✗	✗	✗	✗	✗	✗	✗	✗	✗	✗	✗	✗	✓	✓	✓	✓	✓	✓		✓	✓	✓	✓	✗	✓	37.9
lsb_sleep_diff	✗	✗	✗	✗	✗	✗	✗	✗	✗	✗	✗	✗	✗	✗	✗	✗	✗	✓	✓	✓	✓	✓	✓		✓	✓	✓	✓	✗	✓	37.9
soc_sp_hobby	✓	✓	✓	✓	✗	✗	✗	✓	✓	✗	✗	✗	✗	✗	✗	✗	✗	✓	✓	✓	✓	✓	✓	✓	✓	✓	✓	✓	✗	✗	56.7
**Perception of health, quality of life, development, and functional limitations**																															
poh_sah	✓	✓	✓	✓	✓	✓	✓	✓	✓	✓	✓	✓	✓	✓	✓	✓	✓	✓	✓	✓	✓	✓	✓	✓	✓	✓	✓	✓	✓	✓	100.0
poh_sah_poor	✓	✓	✓	✓	✓	✓	✓	✓	✓	✓	✓	✓	✓	✓	✓	✓	✓	✓	✓	✓	✓	✓	✓	✓	✓	✓	✓	✓	✓	✓	100.0
poh_sah_good	✓	✓	✓	✓	✓	✓	✓	✓	✓	✓	✓	✓	✓	✓	✓	✓	✓	✓	✓	✓	✓	✓	✓	✓	✓	✓	✓	✓	✓	✓	100.0
fct_stairs_diff	✓	✓	✓	✓	✗	✗	✗			✓	✓	✓	✓	✓	✗	✗	✗	✓	✓	✓					✓	✓	✓		✗	✗	65.2
fct_walk_diff	✓	✓	✓	✓	✓	✓	✓	✗	✗	✓	✓	✓	✓	✓	✗	✗	✗	✗	✓	✓	✓	✓	✓	✓	✓	✓	✓	✓	✗	✗	73.3
psy_life_sat_tertile	✓	✓	✓	✓	✗	✗	✗	✓	✗	✗	✗	✗	✗	✗	✓	✓	✓	✓	✓	✓	✓	✓	✓	✓	✓	✓	✓	✓	✗	✗	63.3
psy_life_sat_low	✓	✓	✓	✓	✗	✗	✗	✓	✗	✓	✗	✗	✓	✗	✓	✓	✓	✓	✓	✓	✓	✓	✓	✓	✓	✓	✓	✓	✗	✗	70.0
psy_qol_tertile	✗	✗	✗	✗	✗	✗	✗	✓	✗	✓	✓	✓	✓	✓	✗	✗	✗	✗	✗	✗	✓	✓			✗	✓	✗	✗	✗	✗	32.1
psy_qol_low	✗	✗	✗	✗	✗	✗	✗	✓	✗	✓	✓	✓	✓	✓	✗	✗	✗	✗	✗	✗	✓	✓			✗	✓	✗	✗	✗	✗	32.1
**Diseases**																															
dis_hbp_ever	✓	✓	✓	✓	✓	✓				✓	✓	✓	✓	✓	✗	✗		✗	✓						✓	✓			✓	✓	84.2
dis_angina_ever	✓	✓	✓	✓	✗	✗	✗	✗	✗	✓	✗	✓	✓	✓	✓	✓	✓	✗	✗	✗	✗	✗			✗	✗	✗	✗	✓	✓	46.4
dis_mi_ever	✓	✓	✓	✓	✓	✗	✗			✓	✗	✓	✓	✓	✓	✓	✓	✓	✓	✓					✓	✓	✓		✓	✓	87.0
dis_mi_age	✓	✓	✗	✓	✓	✗	✗			✗	✗	✗	✗	✗	✗	✓	✓	✓	✓	✓					✓	✓	✓		✗	✗	52.2
dis_stroke_ever	✓	✓	✓	✓	✗	✗	✗			✓	✗	✓	✓	✓	✓	✓	✓	✓	✓	✓					✓	✓	✓		✓	✓	82.6
dis_stroke_age	✓	✓	✗	✓	✗	✗	✗			✗	✗	✗	✗	✗	✗	✓	✓	✓	✓	✓					✓	✓	✓		✗	✗	47.8
dis_diab_ever	✓	✓	✓	✓	✓	✓	✗	✗	✗	✓	✓	✓	✓	✓	✓	✓	✓	✓	✓	✓	✓	✓	✓		✓	✓	✓	✓	✗	✓	86.2
dis_diab_age	✓	✗	✗	✓	✓	✗	✗	✗	✗	✓	✗	✓	✓	✗	✗	✓	✓	✓	✓	✓	✓	✓	✓		✓	✓	✓	✓	✗	✗	62.1
**Medication and supplements**																															
dis_hbp_med	✓	✓	✗	✗	✗	✗	✗	✗	✗	✗	✗	✗	✗	✗	✓	✓	✓	✗	✗	✗	✗	✗	✗		✗	✗	✗	✗	✗	✗	17.2
dis_diab_tx	✓	✗	✗	✗	✓	✓	✗	✗	✗	✓	✓	✓	✓	✓	✗	✗	✗	✓	✓	✓	✓	✓	✓		✓	✓	✓	✓	✗	✗	62.1
dis_diab_ins	✓	✗	✗	✗	✓	✓	✗			✓	✓	✓	✓	✓	✗	✗	✗	✓	✓	✓					✓	✓	✓		✗	✗	60.9
**Physical measures and assessments**																															
phy_weight_ms	✓	✓	✗	✗	✗	✗	✗	✗	✗	✓	✗	✓	✓	✗	✓	✓	✓	✓	✓	✓	✓	✓	✓	✓	✓	✓	✓	✓	✗	✗	63.3
phy_weight_sr	✗	✓	✓	✓	✓	✓	✓	✓	✓	✓	✗	✓	✓	✗	✗	✗	✗	✓	✓	✓	✓	✓	✓	✓	✓	✓	✓	✓	✗	✗	73.3
phy_weight_all	✓	✓	✓	✓	✓	✓	✓	✓	✓	✓	✗	✓	✓	✗	✓	✓	✓	✓	✓	✓	✓	✓	✓	✓	✓	✓	✓	✓	✓	✓	93.3
phy_height_ms	✓	✓	✗	✗	✗	✗	✗	✗	✗	✓	✗	✓	✓	✗	✓	✓	✓	✓	✓	✓	✓	✓	✓	✓	✓	✓	✓	✓	✗	✗	63.3
phy_height_sr	✗	✓	✓	✗	✓	✓	✓	✓	✓	✓	✗	✓	✓	✗	✗	✗	✗	✓	✓	✓	✓	✓	✓	✓	✓	✓	✓	✓	✗	✗	70.0
phy_height_all	✓	✓	✓	✗	✓	✓	✓	✓	✓	✓	✗	✓	✓	✗	✓	✓	✓	✓	✓	✓	✓	✓	✓	✓	✓	✓	✓	✓	✓	✓	90.0
phy_bmi_ms	✓	✓	✗	✗	✗	✗	✗	✗	✗	✓	✗	✓	✓	✗	✓	✓	✓	✓	✓	✓	✓	✓	✓	✓	✓	✓	✓	✓	✗	✗	63.3
phy_bmi_sr	✗	✗	✓	✗	✓	✓	✓	✓	✓	✓	✗	✓	✓	✗	✗	✗	✗	✓	✓	✓	✓	✓	✓	✓	✓	✓	✓	✓	✗	✗	66.7
phy_bmi_all	✓	✓	✓	✗	✓	✓	✓	✓	✓	✓	✗	✓	✓	✗	✓	✓	✓	✓	✓	✓	✓	✓	✓	✓	✓	✓	✓	✓	✓	✓	90.0
phy_bmi_cat_ms	✓	✓	✗	✗	✗	✗	✗	✗	✗	✓	✗	✓	✓	✗	✓	✓	✓	✓	✓	✓	✓	✓	✓	✓	✓	✓	✓	✓	✗	✗	63.3
phy_bmi_cat_sr	✗	✗	✗	✗	✓	✓	✓	✓	✓	✓	✗	✓	✓	✗	✗	✗	✗	✓	✓	✓	✓	✓	✓	✓	✓	✓	✓	✓	✗	✗	63.3
phy_bmi_cat_all	✓	✓	✓	✗	✓	✓	✓	✓	✓	✓	✗	✓	✓	✗	✓	✓	✓	✓	✓	✓	✓	✓	✓	✓	✓	✓	✓	✓	✓	✓	90.0
phy_waist_all	✓	✗	✗	✗	✗	✗	✗	✗	✗	✓	✗	✓	✓	✗	✗	✓	✓	✓	✓	✓	✓	✓	✓	✓	✓	✓	✓	✓	✓	✓	63.3
**Cognition, personality, and psychological measures and assessments**																															
psy_anxiety_score	✗	✗	✗	✗	✗	✗	✗	✗	✗	✗	✗	✗	✗	✗	✗	✓	✓	✓	✓	✓	✓	✓	✓	✓	✗	✓	✓	✓	✗	✗	40.0
psy_anxiety_case	✗	✗	✗	✗	✗	✗	✗	✗	✗	✗	✗	✗	✗	✗	✗	✓	✓	✓	✓	✓	✓	✓	✓	✓	✗	✓	✓	✓	✗	✗	40.0
psy_anxious_yn	✓	✓	✗	✗	✗	✓	✓	✓	✓	✗	✗	✗	✗	✗	✓	✓	✓	✗	✗	✗	✗	✗	✗	✗	✗	✗	✗	✗	✗	✗	30.0
cog_imm_rscale	✓	✓	✓	✓	✗	✗	✗	✗	✗	✓	✓	✓	✓	✓	✗	✗	✗	✓	✓	✓	✓	✓	✓	✓	✓	✓	✓	✓	✗	✗	66.7
cog_imm_low	✓	✓	✓	✓	✗	✗	✗	✗	✗	✓	✓	✓	✓	✓	✗	✗	✗	✓	✓	✓	✓	✓	✓	✓	✓	✓	✓	✓	✗	✗	66.7
cog_del_rscale	✓	✗	✓	✓	✗	✗	✗	✗	✗	✓	✓	✓	✓	✓	✗	✗	✗	✓	✓	✓	✓	✓	✓	✓	✓	✓	✓	✓	✗	✗	63.3
cog_del_low	✓	✓	✓	✓	✗	✗	✗	✗	✗	✓	✓	✓	✓	✓	✗	✗	✗	✓	✓	✓	✓	✓	✓	✓	✓	✓	✓	✓	✗	✗	66.7
cog_verb_std	✓	✓	✓	✓	✗	✗	✗	✗	✗	✓	✓	✓	✓	✓	✗	✗	✗	✗	✗	✗	✗	✗	✗	✗	✗	✗	✗	✗	✗	✗	30.0
cog_verb_low	✓	✓	✓	✓	✗	✗	✗	✗	✗	✓	✓	✓	✓	✓	✗	✗	✗	✗	✗	✗	✗	✗	✗	✗	✗	✗	✗	✗	✗	✗	30.0
cog_proc_std	✓	✓	✓	✓	✗	✗	✗	✗	✗	✓	✓	✓	✓	✓	✗	✗	✗	✓	✓	✓	✓	✓	✓	✓	✓	✓	✓	✓	✗	✗	66.7
cog_proc_slow	✓	✓	✓	✓	✗	✗	✗	✗	✗	✓	✓	✓	✓	✓	✗	✗	✗	✓	✓	✓	✓	✓	✓	✓	✓	✓	✓	✓	✗	✗	66.7
cog_comp_std	✓	✓	✓	✓	✗	✗	✗	✗	✗	✓	✓	✓	✓	✓	✗	✗	✗	✗	✗	✗	✗	✗	✗	✗	✗	✗	✗	✗	✗	✗	30.0
cog_comp_case	✓	✓	✓	✓	✗	✗	✗	✗	✗	✓	✓	✓	✓	✓	✗	✗	✗	✗	✗	✗	✗	✗	✗	✗	✗	✗	✗	✗	✗	✗	30.0
cog_mmse_score	✗	✗	✗	✗	✗	✗	✗	✗	✗	✗	✗	✗	✗	✗	✗	✗	✗	✓	✓	✓	✓	✓	✓	✓	✓	✓	✓	✓	✗	✗	36.7
cog_mmse_case	✗	✗	✗	✗	✗	✗	✗	✗	✗	✗	✗	✗	✗	✗	✗	✗	✗	✓	✓	✓	✓	✓	✓	✓	✓	✓	✓	✓	✗	✗	36.7
psy_dep_tertile	✓	✓	✓	✓	✗	✗	✗	✗	✗	✓	✓	✓	✓	✓	✗	✓	✓	✓	✓	✓	✓	✓	✓	✓	✓	✓	✓	✓	✓	✓	80.0

		CLSA_COP		CLSA_TRA		GLOBE					HAPIEE_CZ		HAPIEE_LT	HAPIEE_RU		HUNT			LASA1							LASA2				RECORD		Percent
**DataSchema variable root**		BL	F1	BL	F1	BL	F1	F2	F3	F4	BL	F1	BL	BL	F1	BL	F1	F2	BL	F1	F2	F3	F4	F5	F6	BL	F1	F2	F3	BL	F1	Complete
psy_dep_case	●	✓	✓	✓	✓	✗	✗	✗	✗	✗	✓	✓	✓	✓	✓	✗	✓	✓	✓	✓	✓	✓	✓	✓	✓	✓	✓	✓	✓	✓	✓	80.0
psy_dep_yn	●	✓	✓	✓	✓	✗	✓	✓	✓	✓	✓	✓	✓	✓	✓	✓	✓	✓	✓	✓	✓	✓	✓	✓	✓	✓	✓	✓	✓	✓	✓	96.7
psy_loneliness_tertile	●	✗	✓	✗	✓	✓	✓	✗	✗	✓	✗	✗	✗	✗	✗	✗	✗	✗	✓	✓	✓	✓	✓	✓	✓	✓	✓	✓	✓	✗	✗	53.3
psy_loneliness_high	●	✗	✓	✗	✓	✓	✓	✗	✗	✓	✗	✗	✗	✗	✗	✗	✗	✗	✓	✓	✓	✓	✓	✓	✓	✓	✓	✓	✓	✗	✗	53.3
psy_lonely_yn	●	✓	✓	✓	✓	✓	✓	✗	✗	✗	✓	✓	✓	✓	✓	✗	✓	✓	✓	✓	✓	✓	✓	✓	✓	✓	✓	✓	✓	✗	✗	80.0
psy_ptsd_case	●	✓	✗	✓	✗	✗	✗	✗	✗		✗	✗	✗	✗	✗	✗	✗	✗	✗	✗	✓	✓				✗	✗	✗	✗	✗	✗	15.4
psy_distress_tertile	●	✓	✓	✗	✗	✓	✓	✓	✓	✓	✗	✗	✗	✗	✗	✓	✓	✓	✗	✗	✓	✓	✓	✓	✓	✗	✓	✓	✓	✗	✗	60.0
psy_distress_case	●	✓	✓	✗	✗	✓	✓	✓	✓	✓	✗	✗	✗	✗	✗	✓	✓	✓	✗	✗	✓	✓	✓	✓	✓	✗	✓	✓	✓	✗	✗	60.0
soc_res_satis	●	✗	✗	✗	✗	✗	✗	✓	✓	✓	✗	✗	✗	✗	✗	✗	✗	✗	✓	✓	✓	✓	✗	✗		✓	✗	✗	✗	✓	✓	34.5
**Life events, life plans, beliefs, and values**																																
soc_le_vic	●	✗	✗	✗	✗	✓	✓	✓	✗	✗	✗	✗	✗	✗	✗	✗	✗	✗	✗	✓	✓	✓	✓	✓	✓	✗	✓	✓	✓	✗	✗	40.0
soc_le_fprob	●	✗	✗	✗	✗	✓	✓	✓	✗	✗	✗	✗	✗	✗	✗	✗	✗	✗	✗	✓	✓	✓	✓	✓	✓	✗	✓	✓	✓	✗	✗	40.0
soc_le_rprob	●	✗	✗	✗	✗	✓	✓	✓			✗	✗	✗	✗	✗	✗	✗	✗	✗	✗	✗					✗	✗	✗		✗	✗	13.0
soc_le_divorce	●	✗	✗	✗	✗	✓	✓	✓			✗	✗	✗	✗	✗	✗	✗	✗	✗	✗	✗					✗	✗	✗		✗	✗	13.0
soc_le_illfam	●	✗	✗	✗	✗	✓	✓	✓	✗	✗	✗	✗	✗	✗	✗	✗	✗	✗	✗	✓	✓	✓	✓	✓	✓	✗	✓	✓	✓	✗	✗	40.0
soc_le_dpart	●	✗	✗	✗	✗	✓	✓	✓			✗	✗	✗	✗	✗	✗	✗	✗	✗	✗	✗					✗	✗	✗		✗	✗	13.0
soc_le_dfam	●	✗	✗	✗	✗	✓	✓	✓			✗	✗	✗	✗	✗	✗	✗	✗	✗	✗	✗					✗	✗	✗		✗	✗	13.0
**Social environment and relationships**																																
soc_sn_num_children	●	✓	✗	✓	✓	✗	✗	✗	✗	✗	✗	✗	✓	✗	✗	✗	✗	✗	✓	✓	✓	✓	✓	✓	✓	✓	✓	✓	✓	✓	✓	56.7
soc_sn_children_rank	●	✓	✓	✓	✓	✓	✗	✗	✗	✓	✗	✗	✓	✗	✗	✗	✗	✗	✓	✓	✓	✓	✓	✓	✓	✓	✓	✓	✓	✓	✓	66.7
soc_sn_num_friends	●	✓	✓	✓	✓	✗	✗	✗	✗	✓	✗	✗	✗	✗	✗	✗	✗	✗	✓	✓	✓	✓	✓	✓	✓	✓	✓	✓	✓	✗	✗	53.3
soc_sn_freq_friends_inperson	●	✓	✓	✓	✓	✗	✗	✗	✗	✓	✓	✗	✓	✓	✗	✗	✗	✗	✗	✗	✗	✗	✗			✗	✗	✗	✗	✗	✗	28.6
soc_sn_freq_friends	●	✓	✓	✓	✓	✗	✗	✗	✗	✓	✗	✗	✗	✗	✗	✗	✗	✗	✓	✓	✓	✓	✓	✓	✓	✓	✓	✓	✓	✗	✗	53.3
soc_sn_num_nbhs	●	✗	✗	✓	✓	✗	✗	✗	✗	✓	✗	✗	✗	✗	✗	✗	✗	✗	✓	✓	✓	✓	✓	✓	✓	✓	✗	✗	✓	✗	✗	40.0
soc_sn_freq_nbhs	●	✓	✓	✓	✓	✗	✗	✗	✗	✓	✗	✗	✗	✗	✗	✗	✗	✗	✓	✓	✓	✓	✓	✓	✓	✓	✓	✓	✓	✗	✗	53.3
soc_sn_num_relatives	●	✓	✓	✓	✓	✗	✗	✗	✗	✓	✗	✗	✗	✗	✗	✗	✗	✗	✓	✓	✓	✓	✓	✓	✓	✓	✓	✓	✓	✗	✗	53.3
soc_sn_freq_close_relatives	●	✗	✗	✓	✓	✗	✗	✗	✗	✓	✗	✗	✗	✗	✗	✗	✗	✗	✓	✓	✓	✓	✓	✓	✓	✓	✓	✓	✓	✗	✗	46.7
soc_sn_freq_relatives	●	✓	✓	✓	✓	✗	✗	✗	✗	✓	✓	✗	✓	✓	✗	✗	✗	✗	✓	✓	✓	✓	✓	✓	✓	✓	✓	✓	✓	✗	✗	63.3
soc_sn_num_nonkin	●	✓	✓	✓	✓	✗	✗	✗	✗	✗	✗	✗	✗	✗	✗	✗	✗	✗	✓	✓	✓	✓	✓	✓	✓	✓	✓	✓	✓	✗	✗	50.0
soc_sn_freq_nonkin	●	✓	✓	✓	✓	✗	✗	✗	✗	✗	✗	✗	✗	✗	✗	✗	✗	✗	✗	✓	✓	✓	✓	✓	✓	✓	✓	✓	✓	✗	✗	46.7
soc_ss_received_emo	●	✗	✗	✗	✗	✓	✓	✗	✓	✓	✗	✗	✗	✗	✗	✗	✗	✗	✓	✓	✓	✓	✓	✓	✓	✓	✓	✓	✓	✗	✗	50.0
soc_ss_received_instru	●	✗	✗	✗	✗	✓	✓	✗	✗	✗	✗	✗	✗	✗	✗	✗	✗	✗	✓	✓	✓	✓	✓	✓	✓	✓	✓	✓	✓	✗	✗	43.3
soc_ss_Zscore_perceived_emo	●	✓	✓	✓	✓	✓	✓	✗	✗	✗	✗	✗	✗	✗	✗	✗	✗	✗	✓	✓	✓	✓	✓	✓	✓	✓	✓	✓	✓	✗	✗	56.7
soc_ss_perceived_instru	●	✓	✓	✓	✓	✗	✗	✗	✓	✓	✗	✗	✗	✗	✗	✗	✗	✗	✓	✓	✓	✗	✓	✓	✓	✓	✓	✓	✓	✗	✗	53.3
soc_sp_religious	●	✓	✓	✓	✓	✗	✗	✗	✓	✓	✗	✗	✗	✗	✗	✗	✗	✗	✓	✓	✓	✓	✓	✓	✓	✓	✓	✓	✓	✗	✗	60.0
soc_sp_freq_religious	●	✓	✓	✓	✓	✗	✗	✗	✗	✗	✗	✗	✗	✗	✗	✗	✗	✗	✗	✗	✗	✗	✗	✗	✗	✗	✗	✗	✗	✗	✗	13.3
soc_sp_cultural_edu	●	✓	✓	✓	✓	✗	✗	✗	✗	✓	✗	✗	✗	✗	✗	✗	✗	✗	✓	✓	✓	✓	✓	✓	✓	✓	✓	✓	✓	✗	✗	50.0
soc_sp_cul_edu_soc	●	✗	✗	✗	✗	✗	✗	✓	✗	✗	✗	✗	✗	✗	✗	✗	✗	✗	✓	✓	✓	✓	✓	✓	✓	✓	✓	✓	✓	✗	✗	40.0
soc_sp_volunteer	●	✓	✓	✓	✓	✗	✗	✗	✗	✓	✗	✗	✗	✗	✗	✗	✗	✗	✓	✓	✓	✓	✓	✓	✓	✓	✓	✓	✓	✗	✗	56.7
soc_sp_pol	●	✗	✗	✗	✗	✗	✗	✗	✓	✓	✗	✗	✗	✗	✗	✗	✗	✗	✓	✓	✓	✓	✓	✓	✓	✓	✓	✓	✓	✗	✗	46.7
soc_sp_prof	●	✓	✓	✓	✓	✗	✗	✗	✓	✓	✗	✗	✗	✗	✗	✗	✗	✗	✓	✓	✓	✓	✓	✓	✓	✓	✓	✓	✓	✗	✗	56.7
soc_sp_general	●	✓	✓	✓	✓	✗	✗	✗	✓	✓	✓	✗	✓	✓	✗	✗	✗	✗	✓	✓	✓	✓	✓	✓	✓	✓	✓	✓	✓	✗	✗	70.0
soc_sp_nosafety	●	✓	✓	✓	✓	✗	✗	✗	✗	✗	✗	✗	✗	✗	✗	✗	✗	✗	✓	✗	✗	✗	✓	✓	✗	✓	✓	✓	✗	✗	✗	33.3
soc_sp_notrans	●	✓	✓	✓	✓	✗	✗	✗	✗	✗	✗	✗	✗	✗	✗	✗	✗	✗	✓	✗	✗	✗	✓	✓	✗	✓	✓	✓	✗	✗	✗	33.3
soc_sp_isolation	●	✓	✓	✓	✓	✗	✗	✗	✗	✓	✗	✗	✓	✗	✗	✗	✗	✗	✓	✓	✓	✓	✓	✓	✓	✓	✓	✓	✓	✗	✗	60.0
env_nbh_trust	●	✓	✓	✓	✓	✗	✗	✓	✗	✓	✓	✗	✓	✓	✗	✗	✗	✗	✗	✗	✗	✗	✗			✗	✗	✗	✗	✗	✗	35.7
env_nbh_trust_among	●	✗	✗	✗	✗	✗	✗				✓	✗	✓	✓	✗	✗	✗		✗	✗						✗	✗			✗	✓	21.1
env_nbs_help	●	✗	✗	✗	✗	✗	✗	✓	✗	✓	✓	✗	✓	✓	✗	✗	✗	✗	✗	✗	✗	✗	✗			✗	✗	✗	✗	✗	✗	21.4
env_nbs_coop	●	✓	✓	✓	✓	✗	✗	✓	✓	✓	✗	✗	✗	✗	✗	✗	✗	✗	✗	✗	✗	✗	✗			✗	✗	✗	✗	✓	✗	32.1
env_nbh_values	●	✗	✗	✗	✗	✗	✗	✓	✗	✓	✗	✗	✗	✗	✗	✗	✗	✗	✗	✗	✗	✗	✗			✗	✗	✗	✗	✗	✗	7.1
env_nbs_along	●	✗	✗	✗	✗	✗	✗	✓	✓	✓	✗	✗	✗	✗	✗	✗	✗	✗	✗	✗	✗	✗	✗			✗	✗	✗	✗	✗	✗	10.7
env_nbs_friendly	●	✓		✓		✗					✗		✗	✗		✗			✗							✗				✓		30.0
env_nbh_belong	●	✗	✓	✗	✓	✗	✗	✓	✗	✓	✗	✗	✗	✗	✗	✗	✗	✗	✗	✗	✗	✗	✗			✗	✗	✗	✗	✓	✗	17.9
env_nbh_lone	●	✓	✓	✓	✓	✗	✗	✓	✓	✓	✗	✗	✗	✗	✗	✗	✗	✗	✗	✗	✗	✗	✗			✗	✗	✗	✗	✓	✗	25.0
env_nbh_comm	●	✗	✗	✗	✗	✗	✗	✗	✗	✗	✗	✗	✗	✗	✗	✗	✗	✗	✗	✗	✗	✗	✗			✗	✗	✗	✗	✓	✓	7.1
env_nbh_violence	●	✗	✗	✗	✗	✗	✗	✓			✗	✗	✗	✗	✗	✗	✗	✗	✗	✗	✗					✗	✗	✗		✗	✓	8.7
env_nbs_anti	●	✗	✗	✗	✗	✗	✗	✓			✗	✗	✗	✗	✗	✗	✗	✗	✗	✗	✗					✗	✗	✗		✓	✓	13.0
socenv_urbanization_grade	○						✗	✓	✓	✓	✗	✓						✓					✓		✓		✓		✓	✓	✓	84.6

DataSchema variable root		CLSA_COP BL	CLSA_COP F1	CLSA_TRA BL	CLSA_TRA F1	GLOBE BL	GLOBE F1	GLOBE F2	GLOBE F3	GLOBE F4	HAPIEE_CZ BL	HAPIEE_CZ F1	HAPIEE_LT BL	HAPIEE_RU BL	HAPIEE_RU F1	HUNT BL	HUNT F1	HUNT F2	LASA1 BL	LASA1 F1	LASA1 F2	LASA1 F3	LASA1 F4	LASA1 F5	LASA1 F6	LASA2 BL	LASA2 F1	LASA2 F2	LASA2 F3	RECORD BL	RECORD F1	Percent Complete
socenv_percentage_men	○						✗	✓	✓	✓	✗	✓						✓					✓		✓		✓		✓	✗	✗	69.2
socenv_percentage_women	○						✗	✓	✓	✓	✗	✓						✓					✓		✓		✓		✓	✗	✗	69.2
socenv_percentage_65_older	○						✗	✓	✓	✓	✗	✓						✓					✓		✓		✓		✓	✗	✗	69.2
socenv_percentage_unmarried	○						✗	✓	✓	✓	✗	✓						✗					✓		✓		✓		✓	✗	✗	61.5
socenv_percentage_married	○						✗	✓	✓	✓	✗	✓						✓					✓		✓		✓		✓	✗	✗	69.2
socenv_percentage_divorced	○						✗	✓	✓	✓	✗	✓						✓					✓		✓		✓		✓	✗	✗	69.2
socenv_percentage_widowed	○						✗	✓	✓	✓	✗	✓						✓					✓		✓		✓		✓	✗	✗	69.2
socenv_average_hh_size	○						✗	✓	✓	✓	✗	✓						✓					✓		✓		✓		✓	✗	✓	76.9
socenv_avv_dwellings	○						✗	✓	✓	✓	✗	✗						✗					✓		✓		✓		✓	✗	✗	53.8
socenv_avv_dwelling_m2	○						✗	✗	✗	✗	✗	✓						✗					✗		✗		✗		✗	✗	✗	7.7
socenv_perc_unocc_dwellings	○						✗	✗	✓	✓	✗	✗						✓					✗		✓		✗		✓	✗	✓	46.2
socenv_av_inc1000	○						✗	✓	✓	✓	✗	✗						✓					✓		✓		✓		✓	✗	✗	61.5
socenv_av_inc	○						✗	✗	✗	✗	✗	✓						✓					✗		✗		✗		✗	✗	✗	15.4
socenv_av_incdich1000	○						✗	✓	✓	✓	✗	✗						✓					✓		✓		✓		✓	✗	✗	61.5
socenv_av_incdich	○						✗	✗	✗	✗	✗	✓						✓					✗		✗		✗		✗	✗	✗	15.4
socenv_av_hhinc_500m	○						✗	✗	✗	✗	✗	✗						✗					✗		✗		✗		✗	✓	✗	7.7
socenv_av_hhinc_1000m	○						✗	✗	✗	✗	✗	✗						✗					✗		✗		✗		✗	✓	✗	7.7
socenv_av_hhinc_2000m	○						✗	✗	✗	✗	✗	✗						✗					✗		✗		✗		✗	✓	✗	7.7
socenv_av_hhincdich_500m	○						✗	✗	✗	✗	✗	✗						✗					✗		✗		✗		✗	✓	✗	7.7
socenv_av_hhincdich_1000m	○						✗	✗	✗	✗	✗	✗						✗					✗		✗		✗		✗	✓	✗	7.7
socenv_av_hhincdich_2000m	○						✗	✗	✗	✗	✗	✗						✗					✗		✗		✗		✗	✓	✗	7.7
socenv_av_inc1000Z	○						✗	✓	✓	✓	✗	✗						✓					✓		✓		✓		✓	✗	✗	61.5
socenv_av_incZ	○						✗	✗	✗	✗	✗	✓						✓					✗		✗		✗		✗	✗	✗	15.4
socenv_perc_inc_recipients	○						✗	✓	✓	✓	✗	✗						✗					✓		✓		✓		✓	✗	✗	53.8
socenv_perc_unemployment	○						✗	✗	✗	✗	✗	✓						✓					✗		✗		✗		✗	✗	✓	23.1
socenv_percentage_socsecben	○						✗	✓	✓	✓	✗	✗						✓					✓		✓		✓		✓	✗	✗	61.5
socenv_percentage_disben	○						✗	✓	✓	✓	✗	✗						✗					✓		✓		✓		✓	✗	✗	53.8
socenv_percentage_unemplben	○						✗	✓	✓	✓	✗	✗						✗					✓		✓		✓		✓	✗	✗	53.8
socenv_perc_low_education	○						✗	✗	✓	✓	✗	✓						✓					✗		✓		✗		✓	✗	✗	46.2
socenv_perc_itm_education	○						✗	✗	✓	✓	✗	✓						✓					✗		✓		✗		✓	✗	✗	46.2
socenv_perc_high_education	○						✗	✗	✓	✓	✗	✓						✓					✗		✓		✗		✓	✗	✗	46.2
socenv_crimoff	○						✗	✗	✓	✓	✗	✓						✗					✗		✓		✗		✓	✗	✗	38.5
socenv_vandalism_po	○						✗	✗	✓	✓	✗	✗						✗					✗		✓		✗		✓	✗	✗	30.8
socenv_violentcrim_sexoff	○						✗	✗	✓	✓	✗	✗						✗					✗		✓		✗		✓	✗	✗	30.8
socenv_city	○						✗	✗	✗	✗	✗	✓						✗					✓		✓		✓		✓	✓	✗	46.2
socenv_municipality	○						✗	✓	✓	✓	✗	✓						✓					✓		✓		✓		✓	✓	✓	84.6
socenv_neighbourhood	○						✗	✓	✓	✓	✗	✓						✗					✓		✓		✓		✓	✗	✗	61.5
socenv_pc4	○						✗	✓	✓	✓	✗	✗						✗					✓		✓		✓		✓	✗	✗	53.8
socenv_pc6	○						✗	✓	✓	✓	✗	✗						✗					✓		✓		✓		✓	✗	✗	53.8
socenv_pc5	○						✗	✗	✗	✗	✗	✗						✗					✗		✗		✗		✗	✗	✗	0.0
**Physcial environment**																																
soc_res_type	●	✓	✓	✓	✓	✓	✗	✓	✗	✗	✗	✗	✗	✗	✗	✗	✓	✗	✗	✗	✗	✗	✗	✗	✗	✗	✗	✗	✗	✗	✗	23.3
env_nbh_nsf	●	✗	✗	✗	✗	✗	✗	✓	✗	✗	✓	✗	✓	✓	✗	✗	✗	✗	✓	✓	✓	✓	✓	✓	✓	✓	✓	✓	✓	✓	✓	56.7
env_nbh_sf	●	✗	✗	✗	✗	✗	✗	✓	✗	✗	✓	✗	✓	✓	✗	✗	✗	✗	✓	✓	✓	✓	✓	✓	✓	✓	✓	✓	✓	✓	✓	56.7
env_nbh_clean	●	✓	✓	✓	✓	✗	✗				✗	✗	✗	✗	✗	✗	✗		✗	✗						✗	✗			✓	✓	31.6
env_nbh_clean_dich	●	✓	✓	✓	✓	✗	✗	✓			✗	✗	✗	✗	✗	✗	✗	✗	✗	✗	✗					✗	✗	✗		✓	✓	30.4
env_nbh_noise	●	✗	✗	✗	✗	✗	✗				✗	✗	✗	✗	✗	✗	✗		✗	✗						✗	✗			✓	✓	10.5
env_nbh_noise_dich	●	✓	✓	✓	✓	✓	✓				✗	✗	✗	✗	✗	✗	✗		✗	✗						✗	✗			✓	✓	42.1
env_nbh_traf_noise	●	✗	✗	✗	✗	✓	✓				✗	✗	✗	✗	✗	✗	✗		✗	✗						✗	✗			✗	✓	15.8
env_nbh_gv	●	✓	✓	✓	✓	✗	✗	✓			✗	✗	✗	✗	✗	✗	✗	✗	✗	✗	✗					✗	✗	✗		✓	✓	30.4
env_nbh_theft	●	✗	✗	✗	✗	✗	✗	✓			✗	✗	✗	✗	✗	✗	✗	✗	✗	✗	✗					✗	✗	✗		✗	✓	8.7
physenv_ua_ne_grurb	○						✗	✓	✓	✓	✗	✓						✗					✓		✓		✓		✓	✓	✓	76.9
physenv_ua_ne_forest	○						✗	✓	✓	✓	✗	✓						✗					✓		✓		✓		✓	✓	✓	76.9
physenv_ua_ne_water	○						✗	✓	✓	✓	✗	✓						✗					✓		✓		✓		✓	✓	✓	76.9
physenv_ua_ne_agri	○						✗	✓	✓	✓	✗	✓						✗					✓		✓		✓		✓	✓	✓	76.9
physenv_ua_ne_ttgr	○						✗	✓	✓	✓	✗	✓						✗					✓		✓		✓		✓	✓	✓	76.9
physenv_ua_ne_ttbs	○						✗	✓	✓	✓	✗	✓						✗					✓		✓		✓		✓	✓	✓	76.9
physenv_ua_ne_ttbsgr	○						✗	✓	✓	✓	✗	✓						✗					✓		✓		✓		✓	✓	✓	76.9

		CLSA_COP		CLSA_TRA		GLOBE					HAPIEE_CZ		HAPIEE_LT	HAPIEE_RU		HUNT			LASA1							LASA2				RECORD		Percent
**DataSchema variable root**		BL	F1	BL	F1	BL	F1	F2	F3	F4	BL	F1	BL	BL	F1	BL	F1	F2	BL	F1	F2	F3	F4	F5	F6	BL	F1	F2	F3	BL	F1	Complete
physenv_ua_ne_grurb_as	○						✘	✔	✔	✔	✘	✔						✘					✔		✔		✔		✔	✔	✔	76.9
physenv_ua_ne_forest_as	○						✘	✔	✔	✔	✘	✔						✘					✔		✔		✔		✔	✔	✔	76.9
physenv_ua_ne_water_as	○						✘	✔	✔	✔	✘	✔						✘					✔		✔		✔		✔	✘	✔	69.2
physenv_ua_ne_agri_as	○						✘	✔	✔	✔	✘	✔						✘					✔		✔		✔		✔	✘	✔	69.2
physenv_ua_ne_ttgr_as	○						✘	✔	✔	✔	✘	✔						✘					✔		✔		✔		✔	✔	✔	76.9
physenv_ua_ne_ttbs_as	○						✘	✔	✔	✔	✘	✔						✘					✔		✔		✔		✔	✘	✔	69.2
physenv_ua_ne_ttbsgr_as	○						✘	✔	✔	✔	✘	✔						✘					✔		✔		✔		✔	✔	✔	76.9
physenv_cn_ne_grurb	○						✔	✔	✔	✔	✔	✔						✔					✔		✔		✔		✔	✔	✔	100.0
physenv_cn_ne_forest	○						✔	✔	✔	✔	✔	✔						✔					✔		✔		✔		✔	✔	✔	100.0
physenv_cn_ne_water	○						✔	✔	✔	✔	✔	✔						✔					✔		✔		✔		✔	✔	✔	100.0
physenv_cn_ne_agri	○						✔	✔	✔	✔	✔	✔						✔					✔		✔		✔		✔	✔	✔	100.0
physenv_cn_ne_ttgr	○						✔	✔	✔	✔	✔	✔						✔					✔		✔		✔		✔	✔	✔	100.0
physenv_cn_ne_ttbs	○						✔	✔	✔	✔	✔	✔						✔					✔		✔		✔		✔	✔	✔	100.0
physenv_cn_ne_ttbsgr	○						✔	✔	✔	✔	✔	✔						✔					✔		✔		✔		✔	✔	✔	100.0
physenv_cn_ne_grurb_as	○						✔	✔	✔	✔	✔	✔						✔					✔		✔		✔		✔	✔	✔	100.0
physenv_cn_ne_forest_as	○						✔	✔	✔	✔	✔	✔						✔					✔		✔		✔		✔	✔	✔	100.0
physenv_cn_ne_water_as	○						✔	✔	✔	✔	✔	✔						✔					✔		✔		✔		✔	✔	✔	100.0
physenv_cn_ne_agri_as	○						✔	✔	✔	✔	✔	✔						✔					✔		✔		✔		✔	✔	✔	100.0
physenv_cn_ne_ttgr_as	○						✔	✔	✔	✔	✔	✔						✔					✔		✔		✔		✔	✔	✔	100.0
physenv_cn_ne_ttbs_as	○						✔	✔	✔	✔	✔	✔						✔					✔		✔		✔		✔	✔	✔	100.0
physenv_cn_ne_ttbsgr_as	○						✔	✔	✔	✔	✔	✔						✔					✔		✔		✔		✔	✔	✔	100.0
physenv_ua_be_facil	○						✘	✘	✘	✘	✘	✘						✘					✔		✔		✔		✔	✔	✔	46.2
physenv_ua_be_facil_as	○						✘	✘	✘	✘	✘	✘						✘					✔		✔		✔		✔	✔	✔	46.2
physenv_cn_be_facil	○						✘	✘	✘	✘	✘	✘						✔					✔		✔		✔		✔	✔	✔	53.8
physenv_cn_be_facil_as	○						✘	✘	✘	✘	✘	✘						✔					✔		✔		✔		✔	✔	✔	53.8
physenv_ua_bf_grurb100	○						✘	✔	✔	✔	✘	✔						✘					✔		✔		✔		✔	✔	✔	76.9
physenv_ua_bf_forest100	○						✘	✔	✔	✔	✘	✔						✘					✔		✔		✔		✔	✔	✔	76.9
physenv_ua_bf_water100	○						✘	✔	✔	✔	✘	✔						✘					✔		✔		✔		✔	✔	✔	76.9
physenv_ua_bf_agri100	○						✘	✔	✔	✔	✘	✔						✘					✔		✔		✔		✔	✘	✘	61.5
physenv_ua_bf_ttgr100	○						✘	✔	✔	✔	✘	✔						✘					✔		✔		✔		✔	✔	✔	76.9
physenv_ua_bf_ttbs100	○						✘	✔	✔	✔	✘	✔						✘					✔		✔		✔		✔	✔	✔	76.9
physenv_ua_bf_ttbsgr100	○						✘	✔	✔	✔	✘	✔						✘					✔		✔		✔		✔	✔	✔	76.9
physenv_cn_bf_grurb100	○						✔	✔	✔	✔	✔	✔						✔					✔		✔		✔		✔	✔	✔	100.0
physenv_cn_bf_forest100	○						✔	✔	✔	✔	✔	✔						✔					✔		✔		✔		✔	✔	✔	100.0
physenv_cn_bf_water100	○						✘	✘	✘	✘	✔	✔						✔					✔		✔		✔		✔	✔	✔	69.2
physenv_cn_bf_agri100	○						✔	✔	✔	✔	✔	✔						✘					✔		✔		✔		✔	✘	✘	76.9
physenv_cn_bf_ttgr100	○						✔	✔	✔	✔	✔	✔						✔					✔		✔		✔		✔	✔	✔	100.0
physenv_cn_bf_ttbs100	○						✘	✘	✘	✘	✔	✔						✔					✔		✔		✔		✔	✔	✔	69.2
physenv_cn_bf_ttbsgr100	○						✘	✔	✔	✔	✔	✔						✔					✔		✔		✔		✔	✔	✔	92.3
physenv_ua_bf_grurb400	○						✘	✔	✔	✔	✘	✔						✘					✔		✔		✔		✔	✔	✔	76.9
physenv_ua_bf_forest400	○						✘	✔	✔	✔	✘	✔						✘					✔		✔		✔		✔	✔	✔	76.9
physenv_ua_bf_water400	○						✘	✔	✔	✔	✘	✔						✘					✔		✔		✔		✔	✔	✔	76.9
physenv_ua_bf_agri400	○						✘	✔	✔	✔	✘	✔						✘					✔		✔		✔		✔	✘	✘	61.5
physenv_ua_bf_ttgr400	○						✘	✔	✔	✔	✘	✔						✘					✔		✔		✔		✔	✔	✔	76.9
physenv_ua_bf_ttbs400	○						✘	✔	✔	✔	✘	✔						✘					✔		✔		✔		✔	✔	✔	76.9
physenv_ua_bf_ttbsgr400	○						✘	✔	✔	✔	✘	✔						✘					✔		✔		✔		✔	✔	✔	76.9
physenv_cn_bf_grurb400	○						✔	✔	✔	✔	✔	✔						✔					✔		✔		✔		✔	✔	✔	100.0
physenv_cn_bf_forest400	○						✔	✔	✔	✔	✔	✔						✔					✔		✔		✔		✔	✔	✔	100.0
physenv_cn_bf_water400	○						✔	✔	✔	✔	✔	✔						✔					✔		✔		✔		✔	✔	✔	100.0
physenv_cn_bf_agri400	○						✔	✔	✔	✔	✔	✔						✘					✔		✔		✔		✔	✘	✘	76.9
physenv_cn_bf_ttgr400	○						✔	✔	✔	✔	✔	✔						✔					✔		✔		✔		✔	✔	✔	100.0
physenv_cn_bf_ttbs400	○						✔	✔	✔	✔	✔	✔						✔					✔		✔		✔		✔	✔	✔	100.0
physenv_cn_bf_ttbsgr400	○						✘	✔	✔	✔	✔	✔						✔					✔		✔		✔		✔	✔	✔	92.3
physenv_ua_bf_facil400	○						✘	✔	✔	✔	✘	✘						✘					✔		✔		✔		✔	✘	✘	53.8
physenv_cn_bf_facil400	○						✔	✔	✔	✔	✘	✘						✘					✔		✔		✔		✔	✘	✘	61.5
physenv_ua_bf_grurb800	○						✘	✔	✔	✔	✘	✔						✘					✔		✔		✔		✔	✔	✔	76.9
physenv_ua_bf_forest800	○						✘	✔	✔	✔	✘	✔						✘					✔		✔		✔		✔	✔	✔	76.9
physenv_ua_bf_water800	○						✘	✔	✔	✔	✘	✔						✘					✔		✔		✔		✔	✔	✔	76.9

	CLSA_COP		CLSA_TRA		GLOBE					HAPIEE_CZ		HAPIEE_LT	HAPIEE_RU		HUNT			LASA1							LASA2				RECORD		Percent
**DataSchema variable root**	BL	F1	BL	F1	BL	F1	F2	F3	F4	BL	F1	BL	BL	F1	BL	F1	F2	BL	F1	F2	F3	F4	F5	F6	BL	F1	F2	F3	BL	F1	Complete
physenv_ua_bf_agri800						✗	✓	✓	✓	✗	✓						✗					✓		✓		✓		✓	✗	✗	61.5
physenv_ua_bf_ttgr800						✗	✓	✓	✓	✗	✓						✗					✓		✓		✓		✓	✓	✓	76.9
physenv_ua_bf_ttbs800						✗	✓	✓	✓	✗	✓						✗					✓		✓		✓		✓	✓	✓	76.9
physenv_ua_bf_ttbsgr800						✗	✓	✓	✓	✗	✓						✗					✓		✓		✓		✓	✓	✓	76.9
physenv_cn_bf_grurb800						✓	✓	✓	✓	✓	✓						✓					✓		✓		✓		✓	✓	✓	100.0
physenv_cn_bf_forest800						✓	✓	✓	✓	✓	✓						✓					✓		✓		✓		✓	✓	✓	100.0
physenv_cn_bf_water800						✓	✓	✓	✓	✓	✓						✓					✓		✓		✓		✓	✓	✓	100.0
physenv_cn_bf_agri800						✓	✓	✓	✓	✓	✓						✗					✓		✓		✓		✓	✗	✗	76.9
physenv_cn_bf_ttgr800						✓	✓	✓	✓	✓	✓						✓					✓		✓		✓		✓	✓	✓	100.0
physenv_cn_bf_ttbs800						✓	✓	✓	✓	✓	✓						✓					✓		✓		✓		✓	✓	✓	100.0
physenv_cn_bf_ttbsgr800						✗	✓	✓	✓	✓	✓						✓					✓		✓		✓		✓	✓	✓	92.3
physenv_ua_bf_facil800						✗	✓	✓	✓	✗	✗						✗					✓		✓		✓		✓	✗	✗	53.8
physenv_cn_bf_facil800						✓	✓	✓	✓	✗	✗						✗					✓		✓		✓		✓	✗	✗	61.5
physenv_ua_bf_grurb1000						✗	✓	✓	✓	✗	✓						✗					✓		✓		✓		✓	✓	✓	76.9
physenv_ua_bf_forest1000						✗	✓	✓	✓	✗	✓						✗					✓		✓		✓		✓	✓	✓	76.9
physenv_ua_bf_water1000						✗	✓	✓	✓	✗	✓						✗					✓		✓		✓		✓	✓	✓	76.9
physenv_ua_bf_agri1000						✗	✓	✓	✓	✗	✓						✗					✓		✓		✓		✓	✗	✗	61.5
physenv_ua_bf_ttgr1000						✗	✓	✓	✓	✗	✓						✗					✓		✓		✓		✓	✓	✓	76.9
physenv_ua_bf_ttbs1000						✗	✓	✓	✓	✗	✓						✗					✓		✓		✓		✓	✓	✓	76.9
physenv_ua_bf_ttbsgr1000						✗	✓	✓	✓	✗	✓						✗					✓		✓		✓		✓	✓	✓	76.9
physenv_cn_bf_grurb1000						✓	✓	✓	✓	✓	✓						✓					✓		✓		✓		✓	✓	✓	100.0
physenv_cn_bf_forest1000						✓	✓	✓	✓	✓	✓						✓					✓		✓		✓		✓	✓	✓	100.0
physenv_cn_bf_water1000						✓	✓	✓	✓	✓	✓						✓					✓		✓		✓		✓	✓	✓	100.0
physenv_cn_bf_agri1000						✓	✓	✓	✓	✓	✓						✗					✓		✓		✓		✓	✗	✗	76.9
physenv_cn_bf_ttgr1000						✓	✓	✓	✓	✓	✓						✓					✓		✓		✓		✓	✓	✓	100.0
physenv_cn_bf_ttbs1000						✓	✓	✓	✓	✓	✓						✓					✓		✓		✓		✓	✓	✓	100.0
physenv_cn_bf_ttbsgr1000						✗	✓	✓	✓	✓	✓						✓					✓		✓		✓		✓	✓	✓	92.3
physenv_ua_bf_facil1000						✗	✓	✓	✓	✗	✗						✗					✓		✓		✓		✓	✗	✗	53.8
physenv_cn_bf_facil1000						✓	✓	✓	✓	✗	✗						✗					✓		✓		✓		✓	✗	✗	61.5
physenv_ua_bf_grurb1600						✗	✓	✓	✓	✗	✓						✗					✓		✓		✓		✓	✓	✓	76.9
physenv_ua_bf_forest1600						✗	✓	✓	✓	✗	✓						✗					✓		✓		✓		✓	✓	✓	76.9
physenv_ua_bf_water1600						✗	✓	✓	✓	✗	✓						✗					✓		✓		✓		✓	✓	✓	76.9
physenv_ua_bf_agri1600						✗	✓	✓	✓	✗	✓						✗					✓		✓		✓		✓	✗	✗	61.5
physenv_ua_bf_ttgr1600						✗	✓	✓	✓	✗	✓						✗					✓		✓		✓		✓	✓	✓	76.9
physenv_ua_bf_ttbs1600						✗	✓	✓	✓	✗	✓						✗					✓		✓		✓		✓	✓	✓	76.9
physenv_ua_bf_ttbsgr1600						✗	✓	✓	✓	✗	✓						✗					✓		✓		✓		✓	✓	✓	76.9
physenv_cn_bf_grurb1600						✓	✓	✓	✓	✓	✓						✓					✓		✓		✓		✓	✓	✓	100.0
physenv_cn_bf_forest1600						✓	✓	✓	✓	✓	✓						✓					✓		✓		✓		✓	✓	✓	100.0
physenv_cn_bf_water1600						✓	✓	✓	✓	✓	✓						✓					✓		✓		✓		✓	✓	✓	100.0
physenv_cn_bf_agri1600						✓	✓	✓	✓	✓	✓						✗					✓		✓		✓		✓	✗	✗	76.9
physenv_cn_bf_ttgr1600						✓	✓	✓	✓	✓	✓						✓					✓		✓		✓		✓	✓	✓	100.0
physenv_cn_bf_ttbs1600						✓	✓	✓	✓	✓	✓						✓					✓		✓		✓		✓	✓	✓	100.0
physenv_cn_bf_ttbsgr1600						✗	✓	✓	✓	✓	✓						✓					✓		✓		✓		✓	✓	✓	92.3
physenv_ua_bf_facil1600						✗	✓	✓	✓	✗	✗						✗					✓		✓		✓		✓	✗	✗	53.8
physenv_cn_bf_facil1600						✓	✓	✓	✓	✗	✗						✗					✓		✓		✓		✓	✗	✗	61.5
physenv_ua_bf_facil3000						✗	✓	✓	✓	✗	✗						✗					✓		✓		✓		✓	✗	✗	53.8
physenv_ua_bf_lu100						✗	✓	✓	✓	✗	✓						✗					✓		✓		✓		✓	✓	✓	76.9
physenv_ua_bf_lu100_bu						✗	✓	✓	✓	✗	✓						✗					✓		✓		✓		✓	✓	✓	76.9
physenv_ua_bf_lu100_ic						✗	✓	✓	✓	✗	✓						✗					✓		✓		✓		✓	✓	✓	76.9
physenv_ua_bf_lu100_in						✗	✓	✓	✓	✗	✓						✗					✓		✓		✓		✓	✓	✓	76.9
physenv_ua_bf_lu100_po						✗	✗	✗	✗	✗	✓						✗					✓		✓		✓		✓	✓	✓	53.8
physenv_ua_bf_lu100_grurb						✗	✓	✓	✓	✗	✓						✗					✓		✓		✓		✓	✓	✓	76.9
physenv_ua_bf_lu100_facil						✗	✓	✓	✓	✗	✓						✗					✓		✓		✓		✓	✓	✓	76.9
physenv_ua_bf_lu100_agri						✗	✓	✓	✓	✗	✓						✗					✓		✓		✓		✓	✓	✓	76.9
physenv_ua_bf_lu100_on						✗	✓	✓	✓	✗	✓						✗					✓		✓		✓		✓	✓	✓	76.9
physenv_ua_bf_lu100_water						✗	✓	✓	✓	✗	✓						✗					✓		✓		✓		✓	✓	✓	76.9
physenv_ua_bf_lu100_ot						✗	✓	✓	✓	✗	✓						✗					✓		✓		✓		✓	✓	✓	76.9
physenv_cn_bf_lu100						✗	✗	✗	✗	✓	✓						✗					✗		✗		✗		✗	✗	✗	15.4

DataSchema variable root		CLSA_COP		CLSA_TRA		GLOBE					HAPIEE_CZ		HAPIEE_LT	HAPIEE_RU		HUNT			LASA1							LASA2				RECORD		Percent
		BL	F1	BL	F1	BL	F1	F2	F3	F4	BL	F1	BL	BL	F1	BL	F1	F2	BL	F1	F2	F3	F4	F5	F6	BL	F1	F2	F3	BL	F1	Complete
physenv_cn_bf_lu100_bu	○						✔	✔	✔	✔	✔	✔						✔					✔		✔		✔		✔	✔	✔	100.0
physenv_cn_bf_lu100_ic	○						✔	✔	✔	✔	✔	✔						✔					✔		✔		✔		✔	✔	✔	100.0
physenv_cn_bf_lu100_in	○						✘	✔	✔	✔	✔	✔						✘					✔		✔		✔		✔	✔	✔	84.6
physenv_cn_bf_lu100_po	○						✔	✔	✔	✔	✔	✔						✔					✔		✔		✔		✔	✔	✔	100.0
physenv_cn_bf_lu100_grurb	○						✔	✔	✔	✔	✔	✔						✔					✔		✔		✔		✔	✔	✔	100.0
physenv_cn_bf_lu100_facil	○						✔	✔	✔	✔	✔	✔						✔					✔		✔		✔		✔	✔	✔	100.0
physenv_cn_bf_lu100_agri	○						✔	✔	✔	✔	✔	✔						✔					✔		✔		✔		✔	✔	✔	100.0
physenv_cn_bf_lu100_on	○						✘	✔	✔	✔	✔	✔						✔					✔		✔		✔		✔	✔	✔	92.3
physenv_cn_bf_lu100_water	○						✘	✘	✘	✘	✔	✔						✔					✔		✔		✔		✔	✔	✔	69.2
physenv_cn_bf_lu100_ot	○						✔	✔	✔	✔	✔	✔						✘					✔		✔		✔		✔	✔	✔	92.3
physenv_ua_bf_lu400	○						✘	✔	✔	✔	✘	✔						✘					✔		✔		✔		✔	✔	✔	76.9
physenv_ua_bf_lu400_bu	○						✘	✔	✔	✔	✘	✔						✘					✔		✔		✔		✔	✔	✔	76.9
physenv_ua_bf_lu400_ic	○						✘	✔	✔	✔	✘	✔						✘					✔		✔		✔		✔	✔	✔	76.9
physenv_ua_bf_lu400_in	○						✘	✔	✔	✔	✘	✔						✘					✔		✔		✔		✔	✔	✔	76.9
physenv_ua_bf_lu400_po	○						✘	✘	✘	✘	✘	✔						✘					✔		✔		✔		✔	✔	✔	53.8
physenv_ua_bf_lu400_grurb	○						✘	✔	✔	✔	✘	✔						✘					✔		✔		✔		✔	✔	✔	76.9
physenv_ua_bf_lu400_facil	○						✘	✔	✔	✔	✘	✔						✘					✔		✔		✔		✔	✔	✔	76.9
physenv_ua_bf_lu400_agri	○						✘	✔	✔	✔	✘	✔						✘					✔		✔		✔		✔	✔	✔	76.9
physenv_ua_bf_lu400_on	○						✘	✔	✔	✔	✘	✔						✘					✔		✔		✔		✔	✔	✔	76.9
physenv_ua_bf_lu400_water	○						✘	✔	✔	✔	✘	✔						✘					✔		✔		✔		✔	✔	✔	76.9
physenv_ua_bf_lu400_ot	○						✘	✔	✔	✔	✘	✔						✘					✔		✔		✔		✔	✔	✔	76.9
physenv_cn_bf_lu400	○						✘	✘	✘	✘	✔	✔						✘					✘		✘		✘		✘	✘	✘	15.4
physenv_cn_bf_lu400_bu	○						✔	✔	✔	✔	✔	✔						✔					✔		✔		✔		✔	✔	✔	100.0
physenv_cn_bf_lu400_ic	○						✔	✔	✔	✔	✔	✔						✔					✔		✔		✔		✔	✔	✔	100.0
physenv_cn_bf_lu400_in	○						✔	✔	✔	✔	✔	✔						✘					✔		✔		✔		✔	✔	✔	92.3
physenv_cn_bf_lu400_po	○						✔	✔	✔	✔	✔	✔						✔					✔		✔		✔		✔	✔	✔	100.0
physenv_cn_bf_lu400_grurb	○						✔	✔	✔	✔	✔	✔						✔					✔		✔		✔		✔	✔	✔	100.0
physenv_cn_bf_lu400_facil	○						✔	✔	✔	✔	✔	✔						✔					✔		✔		✔		✔	✔	✔	100.0
physenv_cn_bf_lu400_agri	○						✔	✔	✔	✔	✔	✔						✔					✔		✔		✔		✔	✔	✔	100.0
physenv_cn_bf_lu400_on	○						✔	✔	✔	✔	✔	✔						✔					✔		✔		✔		✔	✔	✔	100.0
physenv_cn_bf_lu400_water	○						✔	✔	✔	✔	✔	✔						✔					✔		✔		✔		✔	✔	✔	100.0
physenv_cn_bf_lu400_ot	○						✔	✔	✔	✔	✔	✔						✔					✔		✔		✔		✔	✔	✔	100.0
physenv_ua_bf_lu800	○						✘	✔	✔	✔	✘	✔						✘					✔		✔		✔		✔	✔	✔	76.9
physenv_ua_bf_lu800_bu	○						✘	✔	✔	✔	✘	✔						✘					✔		✔		✔		✔	✔	✔	76.9
physenv_ua_bf_lu800_ic	○						✘	✔	✔	✔	✘	✔						✘					✔		✔		✔		✔	✔	✔	76.9
physenv_ua_bf_lu800_in	○						✘	✔	✔	✔	✘	✔						✘					✔		✔		✔		✔	✔	✔	76.9
physenv_ua_bf_lu800_po	○						✘	✔	✘	✔	✘	✔						✘					✔		✔		✔		✔	✔	✔	69.2
physenv_ua_bf_lu800_grurb	○						✘	✔	✔	✔	✘	✔						✘					✔		✔		✔		✔	✔	✔	76.9
physenv_ua_bf_lu800_facil	○						✘	✔	✔	✔	✘	✔						✘					✔		✔		✔		✔	✔	✔	76.9
physenv_ua_bf_lu800_agri	○						✘	✔	✔	✔	✘	✔						✘					✔		✔		✔		✔	✔	✔	76.9
physenv_ua_bf_lu800_on	○						✘	✔	✔	✔	✘	✔						✘					✔		✔		✔		✔	✔	✔	76.9
physenv_ua_bf_lu800_water	○						✘	✔	✔	✔	✘	✔						✘					✔		✔		✔		✔	✔	✔	76.9
physenv_ua_bf_lu800_ot	○						✘	✔	✔	✔	✘	✔						✘					✔		✔		✔		✔	✔	✔	76.9
physenv_cn_bf_lu800	○						✘	✘	✘	✘	✔	✔						✘					✘		✘		✘		✘	✘	✘	15.4
physenv_cn_bf_lu800_bu	○						✔	✔	✔	✔	✔	✔						✔					✔		✔		✔		✔	✔	✔	100.0
physenv_cn_bf_lu800_ic	○						✔	✔	✔	✔	✔	✔						✔					✔		✔		✔		✔	✔	✔	100.0
physenv_cn_bf_lu800_in	○						✔	✔	✔	✔	✔	✔						✘					✔		✔		✔		✔	✔	✔	92.3
physenv_cn_bf_lu800_po	○						✔	✔	✔	✔	✔	✔						✔					✔		✔		✔		✔	✔	✔	100.0
physenv_cn_bf_lu800_grurb	○						✔	✔	✔	✔	✔	✔						✔					✔		✔		✔		✔	✔	✔	100.0
physenv_cn_bf_lu800_facil	○						✔	✔	✔	✔	✔	✔						✔					✔		✔		✔		✔	✔	✔	100.0
physenv_cn_bf_lu800_agri	○						✔	✔	✔	✔	✔	✔						✔					✔		✔		✔		✔	✔	✔	100.0
physenv_cn_bf_lu800_on	○						✔	✔	✔	✔	✔	✔						✔					✔		✔		✔		✔	✔	✔	100.0
physenv_cn_bf_lu800_water	○						✔	✔	✔	✔	✔	✔						✔					✔		✔		✔		✔	✔	✔	100.0
physenv_cn_bf_lu800_ot	○						✔	✔	✔	✔	✔	✔						✔					✔		✔		✔		✔	✔	✔	100.0
physenv_ua_bf_lu1000	○						✘	✔	✔	✔	✘	✔						✘					✔		✔		✔		✔	✔	✔	76.9
physenv_ua_bf_lu1000_bu	○						✘	✔	✔	✔	✘	✔						✘					✔		✔		✔		✔	✔	✔	76.9
physenv_ua_bf_lu1000_ic	○						✘	✔	✔	✔	✘	✔						✘					✔		✔		✔		✔	✔	✔	76.9
physenv_ua_bf_lu1000_in	○						✘	✔	✔	✔	✘	✔						✘					✔		✔		✔		✔	✔	✔	76.9

		CLSA_COP		CLSA_TRA		GLOBE					HAPIEE_CZ		HAPIEE_LT	HAPIEE_RU		HUNT			LASA1							LASA2				RECORD		Percent
**DataSchema variable root**		BL	F1	BL	F1	BL	F1	F2	F3	F4	BL	F1	BL	BL	F1	BL	F1	F2	BL	F1	F2	F3	F4	F5	F6	BL	F1	F2	F3	BL	F1	Complete
physenv_ua_bf_lu1000_po	○						✘	✔	✔	✔	✘	✔						✘					✔		✔		✔		✔	✔	✔	76.9
physenv_ua_bf_lu1000_grurb	○						✘	✔	✔	✔	✘	✔						✘					✔		✔		✔		✔	✔	✔	76.9
physenv_ua_bf_lu1000_facil	○						✘	✔	✔	✔	✘	✔						✘					✔		✔		✔		✔	✔	✔	76.9
physenv_ua_bf_lu1000_agri	○						✘	✔	✔	✔	✘	✔						✘					✔		✔		✔		✔	✔	✔	76.9
physenv_ua_bf_lu1000_on	○						✘	✔	✔	✔	✘	✔						✘					✔		✔		✔		✔	✔	✔	76.9
physenv_ua_bf_lu1000_water	○						✘	✔	✔	✔	✘	✔						✘					✔		✔		✔		✔	✔	✔	76.9
physenv_ua_bf_lu1000_ot	○						✘	✔	✔	✔	✘	✔						✘					✔		✔		✔		✔	✔	✔	76.9
physenv_cn_bf_lu1000	○						✘	✘	✘	✘	✔	✔						✘					✘		✘		✘		✘	✘	✘	15.4
physenv_cn_bf_lu1000_bu	○						✔	✔	✔	✔	✔	✔						✔					✔		✔		✔		✔	✔	✔	100.0
physenv_cn_bf_lu1000_ic	○						✔	✔	✔	✔	✔	✔						✔					✔		✔		✔		✔	✔	✔	100.0
physenv_cn_bf_lu1000_in	○						✔	✔	✔	✔	✔	✔						✘					✔		✔		✔		✔	✔	✔	92.3
physenv_cn_bf_lu1000_po	○						✔	✔	✔	✔	✔	✔						✔					✔		✔		✔		✔	✔	✔	100.0
physenv_cn_bf_lu1000_grurb	○						✔	✔	✔	✔	✔	✔						✔					✔		✔		✔		✔	✔	✔	100.0
physenv_cn_bf_lu1000_facil	○						✔	✔	✔	✔	✔	✔						✔					✔		✔		✔		✔	✔	✔	100.0
physenv_cn_bf_lu1000_agri	○						✔	✔	✔	✔	✔	✔						✔					✔		✔		✔		✔	✔	✔	100.0
physenv_cn_bf_lu1000_on	○						✔	✔	✔	✔	✔	✔						✔					✔		✔		✔		✔	✔	✔	100.0
physenv_cn_bf_lu1000_water	○						✔	✔	✔	✔	✔	✔						✔					✔		✔		✔		✔	✔	✔	100.0
physenv_cn_bf_lu1000_ot	○						✔	✔	✔	✔	✔	✔						✔					✔		✔		✔		✔	✔	✔	100.0
physenv_ua_bf_lu1600	○						✘	✔	✔	✔	✘	✔						✘					✔		✔		✔		✔	✔	✔	76.9
physenv_ua_bf_lu1600_bu	○						✘	✔	✔	✔	✘	✔						✘					✔		✔		✔		✔	✔	✔	76.9
physenv_ua_bf_lu1600_ic	○						✘	✔	✔	✔	✘	✔						✘					✔		✔		✔		✔	✔	✔	76.9
physenv_ua_bf_lu1600_in	○						✘	✔	✔	✔	✘	✔						✘					✔		✔		✔		✔	✔	✔	76.9
physenv_ua_bf_lu1600_po	○						✘	✔	✔	✔	✘	✔						✘					✔		✔		✔		✔	✔	✔	76.9
physenv_ua_bf_lu1600_grurb	○						✘	✔	✔	✔	✘	✔						✘					✔		✔		✔		✔	✔	✔	76.9
physenv_ua_bf_lu1600_facil	○						✘	✔	✔	✔	✘	✔						✘					✔		✔		✔		✔	✔	✔	76.9
physenv_ua_bf_lu1600_agri	○						✘	✔	✔	✔	✘	✔						✘					✔		✔		✔		✔	✔	✔	76.9
physenv_ua_bf_lu1600_on	○						✘	✔	✔	✔	✘	✔						✘					✔		✔		✔		✔	✔	✔	76.9
physenv_ua_bf_lu1600_water	○						✘	✔	✔	✔	✘	✔						✘					✔		✔		✔		✔	✔	✔	76.9
physenv_ua_bf_lu1600_ot	○						✘	✔	✔	✔	✘	✔						✘					✔		✔		✔		✔	✔	✔	76.9
physenv_cn_bf_lu1600	○						✘	✘	✘	✘	✔	✔						✘					✘		✘		✘		✘	✘	✘	15.4
physenv_cn_bf_lu1600_bu	○						✔	✔	✔	✔	✔	✔						✔					✔		✔		✔		✔	✔	✔	100.0
physenv_cn_bf_lu1600_ic	○						✔	✔	✔	✔	✔	✔						✔					✔		✔		✔		✔	✔	✔	100.0
physenv_cn_bf_lu1600_in	○						✔	✔	✔	✔	✔	✔						✘					✔		✔		✔		✔	✔	✔	92.3
physenv_cn_bf_lu1600_po	○						✔	✔	✔	✔	✔	✔						✔					✔		✔		✔		✔	✔	✔	100.0
physenv_cn_bf_lu1600_grurb	○						✔	✔	✔	✔	✔	✔						✔					✔		✔		✔		✔	✔	✔	100.0
physenv_cn_bf_lu1600_facil	○						✔	✔	✔	✔	✔	✔						✔					✔		✔		✔		✔	✔	✔	100.0
physenv_cn_bf_lu1600_agri	○						✔	✔	✔	✔	✔	✔						✔					✔		✔		✔		✔	✔	✔	100.0
physenv_cn_bf_lu1600_on	○						✔	✔	✔	✔	✔	✔						✔					✔		✔		✔		✔	✔	✔	100.0
physenv_cn_bf_lu1600_water	○						✔	✔	✔	✔	✔	✔						✔					✔		✔		✔		✔	✔	✔	100.0
physenv_cn_bf_lu1600_ot	○						✔	✔	✔	✔	✔	✔						✔					✔		✔		✔		✔	✔	✔	100.0
physenv_ua_urbcy	○						✘	✔	✔	✔	✘	✘						✔					✔		✔		✔		✔	✔	✔	76.9
physenv_cn_urbcy	○						✔	✔	✘	✘	✘	✘						✘					✔		✔		✔		✔	✘	✘	46.2
**Administrative information**																																
baseline_yr	●	✔		✔		✔					✔		✔	✔		✔			✔							✔				✔		100.0
followup1_yr	●		✔		✔		✔					✔			✔		✔			✔							✔				✔	100.0
followup2_yr	●							✔										✔			✔							✔				100.0
followup3_yr	●								✔													✔							✔			100.0
followup4_yr	●									✔													✔									100.0
followup5_yr	●																							✔								100.0
followup6_yr	●																								✔							100.0
t1	●		✔		✔		✔					✔			✔		✔			✔							✔				✔	100.0
t2	●							✔										✔			✔							✔				100.0
t3	●								✔													✔							✔			100.0
t4	●									✔													✔									100.0
t5	●																							✔								100.0
t6	●																								✔							100.0
**Percent Complete**		66.5	64.1	60.5	57.5	37.7	35.0	70.8	72.1	77.5	42.0	63.3	46.7	46.1	28.1	23.4	33.5	39.5	62.9	65.9	69.6	75.2	86.8	90.4	92.5	63.5	82.4	69.6	86.4	64.7	61.5	
