# Supplementary material for: Overview of retrospective data harmonisation in the MINDMAP project: process and results
Source: J Epidemiol Community Health. 2020 Nov 10;75(5):433–41. doi: 10.1136/jech-2020-214259 (PMC8053335; doi:10.1136/jech-2020-214259)
Supplement: Supplementary data [file jech-2020-214259supp002.pdf]

Table S1. Example of cohort-specific information collected, harmonization potential, and processing algorithms for the variable ‘Number of estimated pack years of the participant’. Note that harmonization processing accounted for longitudinal cross-checks for consistency among data collection events. For example, if a participant responded “Yes” to “Ever smoked cigarettes” at baseline or preceding data collection events, then they would be considered as “Yes” in later follow-ups.

	**DataSchema variable: Number of estimated pack-years of the participant.**<br>**Format: Continuous (decimal)**<br>**Unit: pack-years**			
**# datasets**	**Cohort-specific variables**	**Cohort-specific variable format**	**Harmonization potential**	**Processing Algorithm**
3	1. Smoking pack-years	1. Continuous	Complete	Direct mapping from **[Smoking pack years]**
1	1. Ever smoked cigarettes<br>2. Avg #cig/day<br>3. Total years smoked cigarettes	1. Yes | No<br>2. Continuous<br>3. Continuous	Complete	Values are coded to 0 if **[Ever smoked cigarettes]** = *No*<br>ELSE<br>(**[Avg #cig/day]**/20)***[Total years smoked cigarettes]**
1	1. Ever smoked cigarettes<br>2. Pack-years	1. Yes | No<br>2. Continuous	Complete	Values are coded to 0 if **[Ever smoked cigarettes]** = *No*<br>ELSE<br>Direct mapping from **[pack years]**
1	1. Ever smoked cigarettes<br>2. Cigarettes-year, former smoker<br>3. Cigarettes-year, current smoker	1. Yes | No<br>2. Continuous<br>3. Continuous	Complete	Values are coded to 0 if **[Ever smoked cigarettes]** = *No*<br>ELSE<br>If **[Cigarettes-year, former smoker]** = *Missing*<br>• **[Cigarettes-year, current smoker]**/(20*365)<br>ELSE<br>If **[Cigarettes-year, former smoker]** ≠ *Missing*<br>• **[Cigarettes-year, former smoker]**/(20*365)
1	1. Age of participant<br>2. Current cigarette smoker<br>3. Past cigarette smoker<br>4. Cigarette smoking start age<br>5. Cigarette smoking stop age<br>6. Avg # cig/day past smoker<br>7. Avg # cig/day current smoker	1. Continuous<br>2. Yes | No<br>3. Yes | No<br>4. Continuous<br>5. Continuous<br>6. Continuous<br>7. Continuous	Complete	Values are coded to 0 if **[Current cigarette smoker]** = *No* & **[Past cigarette smoker]** = *No*<br>ELSE<br>If **[Current cigarette smoker]** = *No* & **[Past cigarette smoker]** = *Yes*<br>• (**[Cigarette smoking stop age]** – **[Cigarette smoking start age]**)*(**[Avg # cig/day past smoker]**/20)<br>ELSE<br>If **[Current cigarette smoker]** = *Yes*<br>• (**[Age of participant]** – **[Cigarette smoking start age]**) * (**[Avg # cig/day current smoker]**/20)]
2	1. Qty cig smoked, when daily<br>2. Total yrs daily smoker	1. Categorical<br>2. Continuous	Impossible	N/A
1	Information not available	N/A	Impossible	N/A
