# Supplementary material for: Overview of retrospective data harmonisation in the MINDMAP project: process and results
Source: J Epidemiol Community Health. 2020 Nov 10;75(5):433–41. doi: 10.1136/jech-2020-214259 (PMC8053335; doi:10.1136/jech-2020-214259)
Supplement: Supplementary data [file jech-2020-214259supp003.pdf]

Table S2. DataSchema information for MINDMAP baseline and administrative variables (v2.0 - April 2020). The column # Events indicates the number of data collection events (baseline + follow-ups, up to 7) for which the equivalent variable was created. Equivalent variables in different follow-ups were distinguished by a numeric suffix (e.g., “sdc_age_1” for Age of participant in follow-up 1, “sdc_age_2” for Age of participant in follow-up 2, and so on.) Otherwise variable information was the same for equivalent variables.

Variable name	Format	Label	Description	Categories	# Events
**Sociodemographic and economic characteristics**					
sdc_age_0	integer	Age of participant	Age of the participant.	N/A	7
sdc_gender_0	integer	Gender of participant	Gender of the participant.	0 = Male<br>1 = Female	7
sdc_employ_0	integer	Employment status	Indicator of whether the participant is currently in paid employment or is self-employed.	0 = Currently not in paid employment<br>1 = Currently in paid employment	7
sdc_retire_0	integer	Retirement status	Indicator of whether the participant is currently retired.	0 = Currently not in retirement<br>1 = Currently in retirement	7
sdc_retire_age_0	integer	Retirement age	Age of the participant at retirement.	N/A	3
sdc_highest_edu_0	integer	Highest level of education completed (ISCED 2011)	Highest level of education completed by the participant in accordance with the International Standard Classification of Education (ISCED 2011).	1 = Lower secondary or less (ISCED 0-2)<br>2 = Upper secondary (ISCED 3)<br>3 = Post-secondary non-tertiary education or short-cycle tertiary education (ISCED 4,5)<br>4 = Bachelor,master,doctoral,or equivalent (ISCED 6,7,8)	5
sdc_postsec_edu_0	integer	Post-secondary education	Indicator of whether the participant completed high school or post-secondary education.	0 = Upper secondary or less (ISCED 0-3)<br>1 = Post-secondary non-tertiary education or more (ISCED 4-8)	5
sdc_income_0	integer	Net annual household income	Indicator of whether the net annual household income is above or below the country-specific net mean household income.	0 = Less than or equal to country-specific net mean household income<br>1 = Greater than country-specific net mean household income	7
sdc_civstat_0	integer	Civil status	Indicator of the participant's current civil status.	0 = Currently single or unmarried<br>1 = Currently married or living with partner<br>2 = Currently divorced or separated<br>3 = Currently widowed	7
sdc_livstat_0	integer	Married or living with partner	Indicator of whether the participant is currently married or is currently living with a spouse or a partner in a common household.	0 = Currently not married or living with partner<br>1 = Currently married or living with partner	7
sdc_num_pplhh_0	integer	Number of people in household	Number of people currently living in the same household, including the participant.	N/A	5
sdc_num_childhh_0	integer	Number of children in household	Number of children (biological or adopted) currently living in the same household as the participant.	N/A	5
sdc_num_child_0	integer	Number of children	Number of children (biological or adopted) of the participant.	N/A	5
sdc_child_0	integer	Children (biological or adopted)	Indicator of whether the participant has any children (biological or adopted) or not.	0 = Does not have children<br>1 = Does have children	5
soc_res_num_yrsnbh_0	integer	Residential stability in neighbourhood (individual - level)	Number of years that the participant has lived in the neighbourhood.	N/A	7
soc_res_num_yrshome_0	integer	Residential stability in home (individual - level)	Number of years that participant has lived in current home/address.	N/A	7
soc_res_move_0	integer	Residential mobility (individual - level)	Indicator of whether the participant moved from their current neighbourhood since last interview.	0 = Has not moved since last interview<br>1 = Has moved since last interview	7
soc_res_indep_0	integer	Residence independence	Indicator of whether the participant lives independently or in a nursing/ retirement home.	0 = Lives independently<br>1 = Lives in a nursing/ retirement home	7
soc_res_home_own_dich_0	integer	Home ownership (2-level)	Indicator of whether the participant owns home or not (i.e. pays rent or mortgage).	0 = Does not own home<br>1 = Does own home	7

Variable name	Format	Label	Description	Categories	# Events
soc_res_home_own_0	integer	Home ownership (3-level)	Indicator of whether the participant owns home, pays mortgage or rents.	1 = Owns home<br>2 = Pays mortgage<br>3 = Rents home	3
**Lifestyle and behaviors**					
lsb_alc_cur_0	integer	Current consumption of alcohol	Indicator of whether the participant currently consumes alcohol or has consumed alcohol in the past 12 months.	0 = Does not currently consume alcohol<br>1 = Currently consumes alcohol	7
lsb_alc_ever_0	integer	Ever consumption of alcohol	Indicator of whether the participant has ever consumed alcohol.	0 = Never consumed alcohol<br>1 = Ever consumed alcohol	7
lsb_alc_binge_0	integer	Frequency of binge drinking	Frequency of drinking 4 or more drinks in one day.	1 = 0 to 3 times a month<br>2 = 1 to 2 times a week<br>3 = 3 times a week or more	7
lsb_alc_total_0	decimal	Current quantity of alcohol consumed per week (total)	Current quantity of wine, beer and liquor consumed per week.	N/A	4
lsb_alc_wine_0	decimal	Current quantity of wine consumed per week	Current quantity of wine consumed per week.	N/A	4
lsb_alc_beer_0	decimal	Current quantity of beer consumed per week	Current quantity of beer consumed per week.	N/A	4
lsb_alc_liq_0	decimal	Current quantity of liquor consumed per week	Current quantity of liquor consumed per week.	N/A	4
lsb_smk_cur_0	integer	Current tobacco smoking	Indicator of whether the participant currently smokes any tobacco product.	0 = Currently does not smoke any tobacco product<br>1 = Currently smokes any tobacco product	7
lsb_smk_status_0	integer	Smoking status	Smoking status of the participant.	0 = Never smoker<br>1 = Former smoker<br>2 = Current smoker	7
lsb_smk_ever_0	integer	Ever tobacco smoking	Indicator of whether the participant has ever smoked any tobacco product.	0 = Never smoked tobacco<br>1 = Ever smoked tobacco	7
lsb_cig_qty_0	integer	Quantity cigarettes smoked	Current average number of cigarettes smoked per day.	N/A	7
lsb_smk_start_0	integer	Smoking initiation age	Age at which the participant started smoking any tobacco product.	N/A	7
lsb_smk_stop_0	integer	Smoking cessation age	Age at which the participant last smoked any tobacco product.	N/A	7
lsb_smk_py_0	decimal	Pack years	Number of estimated pack years of the participant.	N/A	3
lsb_pa_walk_freq_0	integer	Frequency of walking	Number of times the participant went for a walk in last week.	0 = Never<br>1 = Seldom (1-2 days)<br>2 = Sometimes (3-4 days)<br>3 = Often (5-7 days)	7
lsb_pa_walk_time_0	integer	Average walking time	Average time the participant spends walking per day.	0 = Less than 30 minutes<br>1 = 30 minutes but less than 1 hour<br>2 = 1 hour but less than 2 hours<br>3 = 2 hours but less than 4 hours<br>4 = 4 hours or more	7
lsb_pa_walk_trans_0	integer	Average walking time for transportation	Minutes per week the participant spent on walking for transportation (utilitarian walking) in the last week.	N/A	5
lsb_pa_walk_leis_0	integer	Average walking time for leisure	Minutes per week the participant spent on walking for leisure in the last week.	N/A	5
lsb_pa_cycle_0	integer	Average cycling time	Average minutes per week the participant spent on cycling in the last week.	N/A	7
lsb_pa_garden_0	integer	Gardening	Indicator of whether the participant does any gardening activities.	0 = Does not do any gardening activities<br>1 = Does any gardening activities	7
lsb_pa_garden_time_0	integer	Average gardening time	Average minutes per week the participant spent on gardening activities in the last week.	N/A	7

Variable name	Format	Label	Description	Categories	# Events
lsb_pa_house_0	integer	Household activities	Indicator of whether the participant does any household activities (light, moderate or vigorous).	0 = Does not do any household activities<br>1 = Does any household activities	7
lsb_pa_house_time_0	integer	Average household activities time	Average minutes per week the participant spent doing household activities in last week.	N/A	7
lsb_pa_sport_0	integer	Sports or exercise	Indicator of whether the participant does any sports or exercise.	0 = Does not do any sports or exercise<br>1 = Does any sports or exercise	7
lsb_pa_sport_time_0	integer	Average sports or exercise time	Average time a week the participant spent on sports or exercise.	0 = never<br>1 = less than 1 hour a week<br>2 = between 1 and 2 hours a week<br>3 = more than 2 hours a week	7
lsb_veg_0	integer	Daily vegetable consumption	Indicator of whether the participant currently consumes vegetables on a daily basis.	0 = Does not consume vegetables on a daily basis<br>1 = Consumes vegetables on a daily basis	3
lsb_fruit_0	integer	Daily fruit consumption	Indicator of whether the participant currently consumes fruit on a daily basis.	0 = Does not consume fruit on a daily basis<br>1 = Consumes fruit on a daily basis	3
lsb_water_0	integer	Daily water consumption	Indicator of whether the participant drinks water on a daily basis.	0 = Does not drink water on a daily basis<br>1 = Drinks water on a daily basis	5
lsb_coffee_qty_0	integer	Quantity daily coffee	Current quantity of coffee the participant consumes per day.	N/A	5
lsb_sleep_hours_0	integer	Average sleep hours	Average number of hours the participant sleeps per night.	N/A	6
lsb_sleep_diff_0	integer	Difficulty falling asleep	Indicator of whether the participant ever has any difficulty falling asleep.	0 = Never has difficulty falling asleep<br>1 = Sometimes has difficulty falling asleep	6
soc_sp_hobby_0	integer	Hobby	Indicator of whether the participant has a hobby.	0 = Does not have a hobby<br>1 = Does have a hobby	7
**Perception of health, quality of life, development, and functional limitations**					
poh_sah_0	integer	Self-assessed health	Self-assessed health of the participant, in three main categories.	0 = Good health<br>1 = Fair health<br>2 = Poor health	7
poh_sah_poor_0	integer	Poor self-assessed health	Dichotomous indicator of self-assessed health of the participant, indicating poor vs non-poor health.	0 = Poor health<br>1 = Non-poor health	7
poh_sah_good_0	integer	Less than good self-assessed health	Dichotomous indicator of self-assessed health of the participant, indicating good vs less than good health.	0 = Less than good health<br>1 = Good health	7
fct_stairs_diff_0	integer	Difficulty walking up and down the stairs	Indicator of whether the participant has difficulty walking up and down the stairs.	0 = Has no difficulty walking up and down the stairs<br>1 = Has difficulty walking up and down the stairs	3
fct_walk_diff_0	integer	Difficulty walking	Indicator of whether the participant has difficulty walking 2 to 3 blocks, or for about 400 to 500 meters, or for 5 minutes.	0 = Not able to walk without difficulty<br>1 = Able to walk without difficulty	7
psy_life_sat_tertile_0	integer	Life satisfaction tertiles	Indicator of whether the participant has high, medium or low levels of life satisfaction measured by tertiles of the scale- or item- specific life satisfaction score.	0 = High levels of life satisfaction<br>1 = Medium levels of life satisfaction<br>2 = Low levels of life satisfaction	7
psy_life_sat_low_0	integer	Lowest level of life satisfaction	Indicator of whether the participant is in the lowest quartile of life satisfaction measured by the scale- or item-specific life satisfaction score.	0 = Second to highest quartile of life satisfaction score<br>1 = Lowest quartile of life satisfaction score	7
psy_qol_tertile_0	integer	Quality of life tertiles	Indicator of whether the participant has high, medium or low levels of quality of life measured by tertiles of the scale-specific quality of life score.	0 = High levels of quality of life<br>1 = Medium levels of quality of life<br>2 = Low levels of quality of life	5
psy_qol_low_0	integer	Lowest level of quality of life	Indicator of whether the participant is in the lowest quintile of quality of life measured by the scale-specific quality of life score.	0 = Second to highest quintile of quality of life score<br>1 = Lowest quintile of quality of life score	5

Variable name	Format	Label	Description	Categories	# Events
**Diseases (ICD-10)**					
dis_hbp_ever_0	integer	Lifetime occurrence of high blood pressure or hypertension	Occurrence of high blood pressure or hypertension at any point during the life of the participant.	0 = Never had high blood pressure or hypertension<br>1 = Ever had high blood pressure or hypertension	2
dis_angina_ever_0	integer	Lifetime occurrence of angina	Occurrence of angina (pain or discomfort in chest) at any point during the life of the participant.	0 = Never had angina<br>1 = Ever had angina	5
dis_mi_ever_0	integer	Lifetime occurrence of myocardial infarction	Occurrence of myocardial infarction (heart attack) at any point during the life of the participant.	0 = Never had myocardial infarction<br>1 = Ever had myocardial infarction	3
dis_mi_age_0	integer	Age at first Myocardial infarction	Age of the participant at time of first myocardial infarction.	N/A	3
dis_stroke_ever_0	integer	Lifetime occurrence of stroke or CVA	Occurrence of stroke or cerebrovascular accident (CVA) at any point during the life of the participant.	0 = Never had stroke or CVA<br>1 = Ever had stroke or CVA	3
dis_stroke_age_0	integer	Age at first stroke or CVA	Age of the participant at time of first stroke or cerebrovascular accident (CVA).	N/A	3
dis_diab_ever_0	integer	Lifetime occurrence of diabetes, borderline diabetes, or high blood sugar	Occurrence of diabetes, borderline diabetes, or high blood sugar, at any point during the life of the participant.	0 = Never had diabetes<br>1 = Ever had diabetes	6
dis_diab_age_0	integer	Age at diagnosis of diabetes, borderline diabetes, or high blood sugar	Age of the participant at first diagnosis of diabetes, borderline diabetes, or high blood sugar.	N/A	6
**Medication and supplements**					
dis_hbp_med_0	integer	Medication for high blood pressure or hypertension	Indicator of whether the participant has ever taken medication for high blood pressure or hypertension.	0 = Never taken medication for high blood pressure or hypertension<br>1 = Ever taken medication for high blood pressure or hypertension	6
dis_diab_tx_0	integer	Currently under treatment for diabetes	Indicator of whether the participant is currently under treatment for diabetes.	0 = Not currently under treatment for diabetes<br>1 = Currently under treatment for diabetes	6
dis_diab_ins_0	integer	Currently use insulin for diabetes	Indicator of whether the participant uses insulin for diabetes treatment.	0 = Currently does not use insulin for diabetes treatment<br>1 = Currently does use insulin for diabetes treatment	3
**Physical measures and assessments**					
phy_weight_ms_0	decimal	Weight in kg - measured	Weight of the participant in kilograms - measured.	N/A	7
phy_weight_sr_0	decimal	Weight in kg - self-reported	Weight of the participant in kilograms - self-reported.	N/A	7
phy_weight_all_0	decimal	Weight in kg - measured and self-reported combined	Weight of the participant in kilograms - measured and self-reported combined.	N/A	7
phy_height_ms_0	decimal	Height in cm - measured	Height of the participant in centimeters - measured.	N/A	7
phy_height_sr_0	decimal	Height in cm - self-reported	Height of the participant in centimeters - self-reported.	N/A	7
phy_height_all_0	decimal	Height in cm - measured and self-reported combined	Height of the participant in centimeters - self-reported and measured combined.	N/A	7
phy_bmi_ms_0	decimal	Body mass index (BMI) - measured	Weight (in kg) divided by height (in m) squared - measured.	N/A	7
phy_bmi_sr_0	decimal	Body mass index (BMI) - self-reported	Weight (in kg) divided by height (in m) squared - self-reported.	N/A	7
phy_bmi_all_0	decimal	Body mass index (BMI) - measured and self-reported combined	Weight (in kg) divided by height (in m) squared - measured and self-reported combined.	N/A	7
phy_bmi_cat_ms_0	integer	Body mass index (BMI) - categories - measured	Body mass index in categories - underweight (<18.5 kg/m2), normal (18.5-24.99 kg/m2), overweight (25 to 29.99 kg/m2), obese (€‰‰¥30 kg/m2) - measured.	0 = Underweight - BMI <18.5<br>1 = Normal weight - BMI 18.5-24.99<br>2 = Overweight - BMI 25-29.99<br>3 = Obese - BMI >=30	7

Variable name	Format	Label	Description	Categories	# Events
phy_bmi_cat_sr_0	integer	Body mass index (BMI) - categories - self-reported	Body mass index in categories - underweight (<18.5 kg/m2), normal (18.5-24.99 kg/m2), overweight (25 to 29.99 kg/m2), obese (€‰‰¥30 kg/m2) - self-reported.	0 = Underweight - BMI <18.5<br>1 = Normal weight - BMI 18.5-24.99<br>2 = Overweight - BMI 25-29.99<br>3 = Obese - BMI >=30	7
phy_bmi_cat_all_0	integer	Body mass index (BMI) - categories - measured and self-reported combined	Body mass index in categories - underweight (<18.5 kg/m2), normal (18.5-24.99 kg/m2), overweight (25 to 29.99 kg/m2), obese (€‰‰¥30 kg/m2) - measured and self-reported combined.	0 = Underweight - BMI <18.5<br>1 = Normal weight - BMI 18.5-24.99<br>2 = Overweight - BMI 25-29.99<br>3 = Obese - BMI >=30	7
phy_waist_all_0	decimal	Waist circumference in cm - measured and self-reported combined	Waist circumference of the participant in centimeters - measured and self-reported combined.	N/A	7
**Cognition, personality, and psychological measures and assessments**					
psy_anxiety_score_0	integer	Anxiety symptom score measured by the Hospital Anxiety & Depression Scale - Anxiety subscale (HADS-A)	Anxiety symptom score ranging from 0 to 21.	N/A	7
psy_anxiety_case_0	integer	Probable caseness of anxiety measured by the Hospital Anxiety & Depression Scale - Anxiety subscale (HADS-A)	Indicator of whether the participant has elevated anxiety symptoms in accordance with the HADS-A threshold from Zigmond & Snaith (1983) and Snaith (2003).	0 = Non-case (0-10 anxiety symptom score)<br>1 = Case (11-21 anxiety symptom score)	7
psy_anxious_yn_0	integer	Feels anxious or nervous	Single item question on whether the participant feels anxious or nervous.	0 = Does not feel anxious or nervous<br>1 = Feels anxious or nervous	7
cog_imm_rscale_0	integer	Immediate word recall	Rescaled value (ranging from 0 to 10) of the total number of words immediately recalled during a test of verbal memory. If the test is performed more than once, data are based on the first trial.	N/A	7
cog_imm_low_0	integer	Low immediate word recall	Indicator of whether the participant has low immediate word recall using a median split on the total (rescaled) number of words immediately recalled. If the test is performed more than once, data are based on the first trial.	0 = High immediate word recall<br>1 = Low immediate word recall	7
cog_del_rscale_0	integer	Delayed word recall	Rescaled value (ranging from 0 to 10) of the total number of words recalled following an interval. If the test is performed more than once, data are based on the first trial.	N/A	7
cog_del_low_0	integer	Low delayed word recall	Indicator of whether the participant has low delayed word recall using a median split on the total (rescaled) number of words recalled following an interval. If the test is performed more than once, data are based on the first trial.	0 = High delayed word recall<br>1 = Low delayed word recall	7
cog_verb_std_0	decimal	Verbal fluency	Standardised value (z-score) of the total number of correctly named words from the given category (animals). If the test is performed more than once, data are based on the first trial.	N/A	7
cog_verb_low_0	integer	Low verbal fluency	Indicator of whether the participant has low verbal fluency using a median split on the total number of correctly named words from the given category (animals). If the test is performed more than once, data are based on the first trial.	0 = High verbal fluency<br>1 = Low verbal fluency	7
cog_proc_std_0	decimal	Processing speed	Standardised value (z-score) of the total number of items recalled correctly in a processing speed task. If the test is performed more than once, data are based on the first trial.	N/A	7

Variable name	Format	Label	Description	Categories	# Events
cog_proc_slow_0	integer	Slow processing speed	Indicator of whether the participant has slow processing speed using a median split on the total number of items recalled correctly. If the test is performed more than once, data are based on the first trial.	0 = Fast processing speed<br>1 = Slow processing speed	7
cog_comp_std_0	decimal	Composite cognition score from individual cognitive tests	Composite cognition standardized value (z-score) obtained by averaging the four individual cognitive test scores (cog_imm_rscale_0, cog_del_rscale_0, cog_verb_std_0 and cog_proc_std_0). Immediate and delayed word recall scores were first standardized to z-scores for comparability across scores.	N/A	7
cog_comp_case_0	integer	Composite cognitive impairment from individual cognitive tests	Indicator of whether the participant has cognitive impairment using a median split on the composite cognition score from individual test scores.	0 = No cognitive impairment<br>1 = Cognitive impairment	7
cog_mmse_score_0	integer	Current cognition score measured by the MMSE	Current cognition score ranging from 0 to 30 measured by the Mini Mental State Examination (MMSE).	N/A	7
cog_mmse_case_0	integer	Current cognitive impairment measured by the MMSE	Indicator of whether the participant has cognitive impairment in accordance with the Mini Mental State Examination (MMSE) threshold from Lezak, Howieson and Loring (2004).	0 = No cognitive impairment (24-30 MMSE score)<br>1 = Cognitive impairment (0-23 MMSE score)	7
psy_dep_tertile_0	integer	Depressive symptom severity tertiles	Indicator of whether the participant has low, medium or high severity of depressive symptoms measured by tertiles of the scale-specific depressive symptom score.	0 = Low severity tertile of depressive symptoms<br>1 = Medium severity tertile of depressive symptoms<br>2 = High severity tertile of depressive symptoms	7
psy_dep_case_0	integer	Probable caseness of depression	Indicator of whether the participant has elevated depressive symptoms in accordance with the scale-specific threshold of depressive symptom score.	0 = Non-case (no elevated depressive symptoms)<br>1 = Case (elevated depressive symptoms)	7
psy_dep_yn_0	integer	Feels sad, downhearted or blue	Indicator of whether the participant feels sad, downhearted or blue (single item question).	0 = Does not feel sad, downhearted or blue<br>1 = Feels sad, downhearted or blue	7
psy_loneliness_tertile_0	integer	Loneliness tertiles	Indicator of whether the participant has low, medium, or high levels of loneliness measured by tertiles of the scale-specific loneliness score.	0 = Low levels of loneliness<br>1 = Medium levels of loneliness<br>2 = High levels of loneliness	7
psy_loneliness_high_0	integer	Highest level of loneliness	Indicator of whether the participant is in the top quintile of loneliness measured by the scale-specific loneliness score.	0 = Second to lowest quintile of loneliness score<br>1 = Top quintile of loneliness score	7
psy_lonely_yn_0	integer	Feels lonely	Indicator of whether the participant feels lonely (single item question).	0 = Does not feel lonely<br>1 = Feels lonely	7
psy_ptsd_case_0	integer	Probable caseness of post-traumatic stress disorder	Indicator of whether the participant has elevated post-traumatic stress disorder (PTSD) symptoms in accordance with the scale-specific threshold of PTSD score.	0 = Non-case (no elevated PTSD symptoms)<br>1 = Case (elevated PTSD symptoms)	4
psy_distress_tertile_0	integer	Psychological distress severity tertiles	Indicator of whether the participant has low, medium or high severity of psychological distress symptoms measured by tertiles of the scale-specific psychological distress score.	0 = Low severity tertile of psychological distress symptoms<br>1 = Medium severity tertile of psychological distress symptoms<br>2 = High severity tertile of psychological distress symptoms	7
psy_distress_case_0	integer	Probable caseness of psychological distress	Indicator of whether the participant has elevated psychological distress symptoms in accordance with the scale-specific threshold of psychological distress score.	0 = Non-case (no elevated psychological distress symptoms)<br>1 = Case (elevated psychological distress symptoms)	7
soc_res_satis_0	integer	Neighbourhood satisfaction	Indicator of whether the participant desires to move away from their current neighbourhood.	0 = Does not want to move out of neighbourhood<br>1 = Does want to move out of neighbourhood	6

Variable name	Format	Label	Description	Categories	# Events
**Life events, life plans, beliefs, and values**					
soc_le_vic_0	integer	Life event: victim	Indicator of whether the participant experienced being the victim of a crime (i.e. theft, assault) in the past few years.	0 = Did not experience being the victim of a crime<br>1 = Did experience being the victim of a crime	7
soc_le_fprob_0	integer	Life event: financial problems	Indicator of whether the participant experienced financial problems in the past few years.	0 = Did not experience financial problems<br>1 = Did experience financial problems	7
soc_le_rprob_0	integer	Life event: relationship problems	Indicator of whether the participant experienced relationship problems in the past few years.	0 = Did not experience relationship problems<br>1 = Did experience relationship problems	3
soc_le_divorce_0	integer	Life event: divorce/relationship breakup	Indicator of the occurrence of divorce/relationship breakup in the past few years.	0 = Did not divorce/ have relationship breakup<br>1 = Did divorce/ had relationship breakup	3
soc_le_illfam_0	integer	Life event: illness of family member	Indicator of whether the participant experienced the illness of family member (including partner) in the past few years.	0 = Did not experience illness of family member<br>1 = Did experience illness of family member	7
soc_le_dpart_0	integer	Life event: death of partner	Indicator of whether the participant experienced the death of partner/spouse in the past few years.	0 = Did not experience death of partner/spouse<br>1 = Did experience death of partner/spouse	3
soc_le_dfam_0	integer	Life event: death of family member or friend	Indicator of whether the participant experienced the death of a child/family member/friend in the past few years.	0 = Did not experience death of child/family member/friend<br>1 = Did experience death of child/family member/friend	3
**Social environment and relationships**					
soc_sn_num_children_0	integer	Network size of children	Number of living biological, adopted or step children.	N/A	7
soc_sn_children_rank_0	integer	Ranked network size of children	Indicator of whether the participant has a low, medium or high network size compromised of children. It is measured by ranking the size of the network which is comprised of any type of child (biological, step, adoptive, foster, in-law son/daughter). Low rank indicates no children. Note: cohorts inquire about different types of children due to the particular questionnaire used, therefore a childless respondent in one study would not necessarily be considered childless in another study.	0 = Low rank of network size comprised of children (childless)<br>1 = Medium rank of network size comprised of children (1 or 2 children)<br>2 = Large rank of network size comprised of children (more than 2 children)	7
soc_sn_num_friends_0	integer	Network size of friends	Number of close friends. These are friends which the participant feels they can talk to/reach out to.	N/A	7
soc_sn_freq_friends_inperson_0	integer	In-person contact frequency with friends	Indicator of the frequency at which the participant has in-person contact with close friends.	1 = Daily to several times per month in-person contact with friends<br>2 = Once per month in-person contact with friends<br>3 = A few times per year, once per year or never in-person contact with friends	5
soc_sn_freq_friends_0	integer	Contact frequency with friends	Indicator of the frequency at which the participant has contact with close friends.	1 = Daily to several times per month contact with friends<br>2 = Once per month contact with friends<br>3 = A few times per year, once per year or never contact with friends	7
soc_sn_num_nbhs_0	integer	Network size of neighbours	Indicator of the number of neighbours that the participant knows well enough to be in touch with or visit.	1 = No neighbours<br>2 = 1 or 2 neighbours<br>3 = 3 to 5 neighbours<br>4 = 6 to 10 neighbours<br>5 = 11 to 20 neighbours<br>6 = More than 20 neighbours	7

Variable name	Format	Label	Description	Categories	# Events
soc_sn_freq_nbhs_0	integer	Contact frequency with neighbours	Indicator of the contact frequency with neighbours that the participant knows well enough to be in touch with or visit.	1 = Daily to several times per month contact with neighbours<br>2 = Once per month contact with neighbours<br>3 = A few times per year, once per year or never contact with neighbours	7
soc_sn_num_relatives_0	integer	Network size of relatives	Number of relatives, including siblings and excluding partner/children living at home, which the participant is close to, i.e. is in touch with regularly.	1 = None<br>2 = 1 or 2<br>3 = 3 to 5<br>4 = 6 to 10<br>5 = 11 to 20<br>6 = More than 20	7
soc_sn_freq_close_relatives_0	integer	Contact frequency with family	Indicator of the contact frequency with relatives including siblings and children, which participant is close to.	1 = Daily to less than monthly (once per month) contact with family<br>2 = Once per month contact with family<br>3 = A few times per year, once per year or never contact with family	7
soc_sn_freq_relatives_0	integer	Contact frequency with relatives	Indicator of the contact frequency with relatives, including siblings and children regardless of how close the participant feels to them.	1 = Daily to less than monthly (once per month) contact with relatives<br>2 = Once per month contact with relatives<br>3 = A few times per year, once per year or never contact with relatives	7
soc_sn_num_nonkin_0	integer	Network size of non-kin	Number of non-kin members of the participant€™s social network consisting of neighbours, colleagues (from work or school), members of associations (that participant is a part of, such as community activities and organization) and friends.	N/A	7
soc_sn_freq_nonkin_0	integer	Contact frequency with non-kin	Indicator of the contact frequency with non-kin members of social network, consisting of neighbours, relatives and friends.	1 = Daily to less than monthly (once per month) contact with non-kin members<br>2 = Once per month contact with non-kin members<br>3 = A few times per year, once per year or never contact with non-kin members	7
soc_ss_received_emo_0	integer	Received emotional support	Indicator of the frequency of received emotional support i.e. receiving affection from or sharing personal experiences/feelings with, from the participant€™s three social network members that they indicated they are closest to.	0 = Never received emotional support<br>1 = Sometimes received emotional support<br>2 = Often received emotional support	7
soc_ss_received_instru_0	integer	Received instrumental support	Indicator of the frequency of received instrumental support, i.e. tangible help, from the participant€™s three social network members that they indicated they are closest to.	0 = Never received instrumental support<br>1 = Sometimes received instrumental support<br>2 = Often received instrumental support	7
soc_ss_Zscore_perceived_emo_0	decimal	Perceived emotional support Z-score	Z-score of perceived emotional support.	N/A	7
soc_ss_perceived_instru_0	integer	Perceived instrumental support	Indicator of perceived instrumental support, i.e. participant feels like there is someone to help with daily chores in case of need.	0 = Feels tangible help is available<br>1 = Does not feel tangible help is available	7
soc_sp_religious_0	integer	Religious participation	Indicator of participation in a religious organization.	0 = Does not participate in a religious organization<br>1 = Does participate in a religious organization	7

Variable name	Format	Label	Description	Categories	# Events
soc_sp_freq_religious_0	integer	Frequency of religious participation	Indicator of the frequency of participation in a religious organization.	1 = Weekly participation in religious organization<br>2 = Less than monthly participation in religious organization<br>3 = Monthly participation in religious organization<br>4 = Never participates in religious organization<br>5 = No answer/donâ€™t know regarding participation in religious organization	7
soc_sp_cultural_edu_0	integer	Cultural and educational participation	Indicator of whether the participant is a member of/participates in a group, club or activity related to education or culture.	0 = Does not participate in a cultural or educational organization<br>1 = Does participate in a cultural or educational organization	7
soc_sp_cul_edu_soc_0	integer	Cultural, educational and social participation	Indicator of whether the participant is a member of/ participates in a group, club or activity related to culture, educational or social activities.	0 = Does not participate in a cultural, educational or social organization<br>1 = Does participate in a cultural, educational or social organization	7
soc_sp_volunteer_0	integer	Volunteering	Indicator of whether the participant takes part in volunteer activities including charity work or volunteer work in an association with a social aim.	0 = Does not take part in volunteer activities<br>1 = Does take part in volunteer activities	7
soc_sp_pol_0	integer	Political / union participation	Indicator of participation in a union or political party.	0 = Does not participate in a civic organization<br>1 = Does participate in a civic organization	7
soc_sp_prof_0	integer	Professional participation	Indicator of participation in a professional association, including a union or neighbourhood association.	0 = Does not participate in an union, professional or neighbourhood organization<br>1 = Does participate in an union, professional or neighbourhood organization	7
soc_sp_general_0	integer	General social participation	Indicator of participation in a religious, cultural, educational, civic, political, social/charity work association and/or volunteering.	0 = Does not take part in a social organization/volunteers<br>1 = Does take part in a social organization/volunteers	7
soc_sp_nosafety_0	integer	Non participation due to safety	Indicator of whether the participant did not participate in social activities due to safety concerns.	0 = Safety concerns did not affect participation<br>1 = Did not participate due to safety concerns	7
soc_sp_notrans_0	integer	Non participation due to lack of transportation	Indicator of whether the participant did not participate in social activities due to transport issues.	0 = Lack of transportation did not affect participation<br>1 = Did not participate due to lack of transportation	7
soc_sp_isolation_0	integer	Social isolation	Indicator of whether the participant is socially isolated i.e. no/ low social network and no participation in an association (any type, including religious organization and volunteering).	0 = Not socially isolated, has social network and/ or social participation<br>1 = Socially isolated, no social network and no social participation	7
env_nbh_trust_0	integer	Trust in neighbours	Indicator of whether the participant agrees that most people in the area where they live (i.e. in their neighbourhood) can be trusted.	0 = People cannot be trusted<br>1 = People can be trusted	5
env_nbh_trust_among_0	integer	Neighbourhood trust	Indicator of whether the participant agrees that there is trust among the people in the area where they live (i.e. in their neighbourhood).	0 = People do not trust one another<br>1 = People trust one another	2
env_nbs_help_0	integer	Helpful neighbours	Indicator of whether the participant agrees that neighbors would help them if needed, in case of emergency/trouble.	0 = Neighbours would not help me<br>1 = Neighbours would help me	5
env_nbs_coop_0	integer	Cooperating neighbours	Indicator of whether the participant agrees that members of the area where they live (i.e. their community or their neighbourhood) help each other out.	0 = People not willing to help each other<br>1 = People willing to help each other	5

Variable name	Format	Label	Description	Categories	# Events
env_nbh_values_0	integer	Neighbourhood values and norms	Indicator of whether the participant agrees that people in their neighbourhood share similar values and norms.	1 = Strongly agree that people share similar values and norms<br>2 = Agree that people share similar values and norms<br>3 = Neither agree or disagree that people share similar values and norms<br>4 = Disagree that people share similar values and norms<br>5 = Strongly disagree that people share similar values and norms	5
env_nbs_along_0	integer	Neighbours get along	Indicator of whether the participant agrees that people in their neighbourhood get along with each other.	1 = Strongly agree that people get along<br>2 = Agree that people get along<br>3 = Neither agree or disagree that people get along<br>4 = Disagree that people get along<br>5 = Strongly disagree that people get along	5
env_nbs_friendly_0	integer	Friendly neighbours	Indicator of whether the participant is asked whether members of the area where their live (i.e. their neighbourhood) are friendly/ polite.	1 = People are friendly<br>2 = People are somewhat friendly<br>3 = People are not really friendly<br>4 = People are not friendly	1
env_nbh_belong_0	integer	Belonging to neighbourhood	Indicator of whether the participant asked whether they feel a part of the area where they live (i.e. their community, their neighbourhood).	0 = Does not feel part of area<br>1 = Feels part of area	5
env_nbh_lone_0	integer	Lonely in neighbourhood	Indicator of whether the participant agrees that they feel lonely in the area where they live (i.e. their community, their neighbourhood).	1 = Strongly agree that they feel lonely<br>2 = Agree that they feel lonely<br>3 = Neither agree or disagree that they feel lonely<br>4 = Disagree that they feel lonely<br>5 = Strongly disagree that they feel lonely	5
env_nbh_comm_0	integer	Close-knit community	Indicator of whether the participant agrees that members of the area in which participant lives are close-knit (community solidarity).	1 = Strong neighbourhood solidarity<br>2 = Some neighbourhood solidarity<br>3 = A little neighbourhood solidarity<br>4 = No neighbourhood solidarity	5
env_nbh_violence_0	integer	Neighbourhood violence	Indicator of the participant's perception of violence in their neighbourhood.	0 = Violence in neighbourhood<br>1 = No violence in neighbourhood	3
env_nbs_anti_0	integer	Neighbourhood antisocial and aggressive behaviour	Indicator of the participant's perception of antisocial and aggressive behaviour in their neighbourhood.	0 = Antisocial and aggressive behavior in neighbourhood<br>1 = No antisocial and aggressive behavior in neighbourhood	3
socenv_urbanization_grade_0	integer	Urbanization grade	Population density of the residential area (number of residents per km2).	N/A	7
socenv_percentage_men_0	decimal	Percentage of men	Percentage of men in the residential area.	N/A	7
socenv_percentage_women_0	decimal	Percentage of women	Percentage of women in the residential area.	N/A	7
socenv_percentage_65_older_0	decimal	Percentage of residents aged 65 or older	Percentage of residents aged 65 or older in residential area.	N/A	7
socenv_percentage_unmarried_0	decimal	Percentage of unmarried residents	Percentage of unmarried residents in residential area.	N/A	7
socenv_percentage_married_0	decimal	Percentage of married residents	Percentage of married residents in residential area.	N/A	7
socenv_percentage_divorced_0	decimal	Percentage of divorced residents	Percentage of divorced residents in residential area.	N/A	7
socenv_percentage_widowed_0	decimal	Percentage of widowed residents	Percentage of widowed residents in residential area.	N/A	7
socenv_average_hh_size_0	integer	Average household size	Average household size in residential area.	N/A	7
socenv_avv_dwellings_0	integer	Average value of dwellings in 1000 euros	Average value of dwellings in 1000 euros in residential area.	N/A	7

Variable name	Format	Label	Description	Categories	# Events
socenv_avv_dwelling_m2_0	integer	Average value of a plot (m2) in euros (dwellings)	Average value of a plot in euros (dwellings) in the residential area.	N/A	7
socenv_perc_unocc_dwellings_0	decimal	Percentage of unoccupied dwellings	Percentage of unoccupied dwellings in residential area.	N/A	7
socenv_av_inc1000_0	integer	Average income in 1000 euros	Average income in 1000 euros of residents in residential area.	N/A	7
socenv_av_inc_0	integer	Average income in euros	Average income in euros of residents in residential area.	N/A	7
socenv_av_incdich1000_0	integer	Average income in 1000 euros (dichotomized)	Average income in 1000 euros of residents in residential area (dichotomized: below/above country-specific mean household income).	0 = Less than or equal to country-specific mean household income<br>1 = Greater than country-specific mean household income	7
socenv_av_incdich_0	integer	Average income in euros (dichotomized)	Average income in euros of residents in residential area (dichotomized: below/above country-specific mean household income).	0 = Less than or equal to country-specific mean household income<br>1 = Greater than country-specific mean household income	7
socenv_av_hhinc_500m_0	integer	Average household income in euros	Average household income in euros in 500 meter street network buffer zone around participants address.	N/A	7
socenv_av_hhinc_1000m_0	integer	Average household income in euros	Average household income in euros in 1000 meter street network buffer zone around participants address.	N/A	7
socenv_av_hhinc_2000m_0	integer	Average household income in euros	Average household income in euros in 2000 meter street network buffer zone around participants address.	N/A	7
socenv_av_hhincdich_500m_0	integer	Average household income in euros (dichotomized)	Average household income in euros in 500 meter street network buffer zone around participants address (dichotomized: below/above country-specific mean household income).	0 = Less than or equal to country-specific mean household income<br>1 = Greater than country-specific mean household income	7
socenv_av_hhincdich_1000m_0	integer	Average household income in euros (dichotomized)	Average household income in euros in 1000 meter street network buffer zone around participants address (dichotomized: below/above country-specific mean household income).	0 = Less than or equal to country-specific mean household income<br>1 = Greater than country-specific mean household income	7
socenv_av_hhincdich_2000m_0	integer	Average household income in euros (dichotomized)	Average household income in euros in 2000 meter street network buffer zone around participants address (dichotomized: below/above country-specific mean household income).	0 = Less than or equal to country-specific mean household income<br>1 = Greater than country-specific mean household income	7
socenv_av_inc1000Z_0	integer	Average income in 1000 euros (Z standardised)	Average income in 1000 euros of residents in residential area (standardized: mean=0 and sd=1).	N/A	7
socenv_av_incZ_0	integer	Average income in euros (Z standardized)	integer	N/A	7
socenv_perc_inc_recipients_0	decimal	Percentage of income recipients	Percentage of income recipients in residential area.	N/A	7
socenv_perc_unemployment_0	decimal	Percentage of unemployed residents	Percentage of unemployed residents in residential area.	N/A	7
socenv_percentage_socsecben_0	decimal	Percentage of social security beneficiaries	Percentage of social security beneficiaries in residential area.	N/A	7
socenv_percentage_disben_0	decimal	Percentage of residents with disability benefit	Percentage of residents with a disability benefit in residential area.	N/A	7
socenv_percentage_unemplben_0	decimal	Percentage of residents with unemployment benefit	Percentage of residents with a unemployment benefit in residential area.	N/A	7
socenv_perc_low_education_0	decimal	Percentage of low educated residents	Percentage of low educated residents in residential area.	N/A	7
socenv_perc_itm_education_0	decimal	Percentage of intermediate educated residents	Percentage of intermediate educated residents in residential area.	N/A	7
socenv_perc_high_education_0	decimal	Percentage of high educated residents	Percentage of high educated residents in residential area.	N/A	7

Variable name	Format	Label	Description	Categories	# Events
socenv_crimoff_0	integer	Number of criminal offenses per 1000 residents	Number of criminal offenses per 1000 residents in residential area.	N/A	7
socenv_vandalism_po_0	integer	Number of vandalism and public disorder per 1000 residents	Number of vandalism and public disorder per 1000 residents in residential area.	N/A	7
socenv_violentcrim_sexoff_0	integer	Number of violent crimes and sexual offenses per 1000 residents	Number of violent crimes and sexual offenses per 1000 residents in residential area.	N/A	7
socenv_city_0	integer	Area indicator: city	City indicator.	N/A	7
socenv_municipality_0	integer	Area indicator: municipality	Municipality indicator.	N/A	7
socenv_neighbourhood_0	integer	Area indicator: neighbourhood	Neighbourhood indicator.	N/A	7
socenv_pc4_0	integer	Area indicator: 4-digit postal code	4-digit postal code (PC4) indicator.	N/A	7
socenv_pc6_0	integer	Area indicator: 6-digit postal code	6-digit postal code (PC6) indicator.	N/A	7
socenv_pc5_0	integer	Area indicator: 5-digit postal code	5-digit postal code (PC5) indicator.	N/A	7

Physcial environment

Variable name	Format	Label	Description	Categories	# Events
soc_res_type_0	integer	Residence type	Indicator of type of home in which participant lives.	1 = Lives in a flat/ apartment<br>2 = Lives in a single-family house (terraced house/ townhouse, semi-detached, detached)<br>3 = Lives in a nursing home, retirement home, home for the elderly<br>4 = Lives on a farm<br>5 = Other	7
env_nbh_nsf_0	integer	Feels safe at night	Indicator of whether the participant agrees that they feel safe at night in the area where they live (i.e. their neighbourhood).	0 = Does not feel safe at night<br>1 = Feels safe at night	7
env_nbh_sf_0	integer	Feels safe in neighbourhood	Indicator of whether the participant agrees that they feel safe in their neighbourhood.	0 = Does not feel safe<br>1 = Feels safe	7
env_nbh_clean_0	integer	Neighbourhood cleanliness (4-level)	Indicator of the participant's perception of the cleanliness (presence of rubbish) of the area where the respondent lives (i.e. their neighbourhood).	1 = Area not very clean<br>2 = Area not clean<br>3 = Area mostly clean<br>4 = Area clean	2
env_nbh_clean_dich_0	integer	Neighbourhood cleanliness (2-level)	Indicator of the participant's perception of the cleanliness (presence of rubbish) of the area where the respondent lives (i.e. their neighbourhood).	0 = Area not clean<br>1 = Area clean	3
env_nbh_noise_0	integer	Neighbourhood noisiness (4-level)	Indicator of the participant's perception of presence of excessive noise in their neighbourhood.	1 = Definitely excessive noise<br>2 = Some excessive noise<br>3 = A little excessive noise<br>4 = No excessive noise	2
env_nbh_noise_dich_0	integer	Neighbourhood noisiness (2-level)	Indicator of the participant's perception of presence of excessive noise in their neighbourhood, dichotomized.	0 = Noisy<br>1 = Not noisy	2
env_nbh_traf_noise_0	integer	Neighbourhood traffic noise	Indicator of the participant's perception of presence of noise from traffic, in their neighbourhood.	0 = Traffic noise<br>1 = No traffic noise	2
env_nbh_gv_0	integer	Neighbourhood graffiti and vandalism	Indicator of the participant's perception of graffiti and/or vandalism in the area where the respondent lives (i.e. their neighbourhood).	0 = No graffiti and no vandalism<br>1 = Graffiti and/or vandalism	3
env_nbh_theft_0	integer	Theft of transportation method	Indicator of the participant's perception of the occurrence of theft of transport vehicle in their neighbourhood.	0 = Theft of vehicle in neighbourhood<br>1 = No theft of vehicle in neighbourhood	3
physenv_ua_ne_grurb_0	decimal	Distance to green urban areas (Urban Atlas)	Absolute distance to the nearest polygon marked as green urban areas from Urban Atlas data.	N/A	7

Variable name	Format	Label	Description	Categories	# Events
physenv_ua_ne_forest_0	decimal	Distance to forest (Urban Atlas)	Absolute distance to the nearest polygon marked as forest from Urban Atlas data.	N/A	7
physenv_ua_ne_water_0	decimal	Distance to water (Urban Atlas)	Absolute distance to the nearest polygon marked as water from Urban Atlas data.	N/A	7
physenv_ua_ne_agri_0	decimal	Distance to agricultural land (Urban Atlas)	Absolute distance to the nearest polygon. No distinction is made between different types of agricultural land from Urban Atlas data.	N/A	7
physenv_ua_ne_ttgr_0	decimal	Distance to total green spaces (forests + green urban areas) (Urban Atlas)	Absolute distance to the nearest polygon. Total of all categories of green spaces (forests + green urban areas) from Urban Atlas data.	N/A	7
physenv_ua_ne_ttbs_0	decimal	Distance to total blue spaces (water + wetlands) (Urban Atlas)	Absolute distance to the nearest polygon. Total of all categories of blue spaces (water + wetlands) from Urban Atlas data.	N/A	7
physenv_ua_ne_ttbsgr_0	decimal	Distance to total blue and green spaces (water, wetlands, forests, green urban areas) (Urban Atlas)	Absolute distance to the nearest polygon. Total of both green and blue spaces (water, wetlands, forests, green urban areas) from Urban Atlas data.	N/A	7
physenv_ua_ne_grurb_as_0	decimal	Area size of green urban areas (Urban Atlas)	Area size of the nearest polygon marked as green urban areas from Urban Atlas data.	N/A	7
physenv_ua_ne_forest_as_0	decimal	Area size of forest (Urban Atlas)	Area size of the nearest polygon marked as forest from Urban Atlas data.	N/A	7
physenv_ua_ne_water_as_0	decimal	Area size of water (Urban Atlas)	Area size of the nearest polygon marked as water from Urban Atlas data.	N/A	7
physenv_ua_ne_agri_as_0	decimal	Area size agricultural land (Urban Atlas)	Area size of the nearest polygon. No distinction is made between different types of agricultural land from Urban Atlas data.	N/A	7
physenv_ua_ne_ttgr_as_0	decimal	Area size of total green spaces (forests + green urban areas) (Urban Atlas)	Area size of the nearest polygon. Total of all categories of green spaces (forests + green urban areas) from Urban Atlas data.	N/A	7
physenv_ua_ne_ttbs_as_0	decimal	Area size of total blue spaces (water + wetlands) (Urban Atlas)	Area size of the nearest polygon. Total of all categories of blue spaces (water + wetlands) from Urban Atlas data.	N/A	7
physenv_ua_ne_ttbsgr_as_0	decimal	Area size of total blue and green space (water, wetlands, forests, green urban areas) (Urban Atlas)	Area size of the nearest polygon. Total of both green and blue spaces (water, wetlands, forests, green urban areas) from Urban Atlas data.	N/A	7
physenv_cn_ne_grurb_0	decimal	Distance to green urban areas (CORINE)	Absolute distance to the nearest polygon marked as green urban areas from CORINE data.	N/A	7
physenv_cn_ne_forest_0	decimal	Distance to forest (CORINE)	Absolute distance to the nearest polygon marked as forest from CORINE data.	N/A	7
physenv_cn_ne_water_0	decimal	Distance to water (CORINE)	Absolute distance to the nearest polygon marked as water from CORINE data.	N/A	7
physenv_cn_ne_agri_0	decimal	Distance to agricultural land (CORINE)	Absolute distance to the nearest polygon. No distinction is made between different types of agricultural land from CORINE data.	N/A	7
physenv_cn_ne_ttgr_0	decimal	Distance to total green spaces (forests + green urban areas) (CORINE)	Absolute distance to the nearest polygon. Total of all categories of green spaces (forests + green urban areas) from CORINE data.	N/A	7
physenv_cn_ne_ttbs_0	decimal	Distance to total blue spaces (water + wetlands) (CORINE)	Absolute distance to the nearest polygon. Total of all categories of blue spaces (water + wetlands) from CORINE data.	N/A	7
physenv_cn_ne_ttbsgr_0	decimal	Distance to total blue and green spaces (water, wetlands, forests, green urban areas) (CORINE)	Absolute distance to the nearest polygon. Total of both green and blue spaces (water, wetlands, forests, green urban areas) from CORINE data.	N/A	7

Variable name	Format	Label	Description	Categories	# Events
physenv_cn_ne_grurb_as_0	decimal	Area size of green urban areas (CORINE)	Area size of the nearest polygon marked as green urban areas from CORINE data.	N/A	7
physenv_cn_ne_forest_as_0	decimal	Area size of forest (CORINE)	Area size of the nearest polygon marked as forest from CORINE data.	N/A	7
physenv_cn_ne_water_as_0	decimal	Area size of water (CORINE)	Area size of the nearest polygon marked as water from CORINE data.	N/A	7
physenv_cn_ne_agri_as_0	decimal	Area size of agricultural land (CORINE)	Area size of the nearest polygon. No distinction is made between different types of agricultural land from CORINE data.	N/A	7
physenv_cn_ne_ttgr_as_0	decimal	Area size of total green spaces (forests + green urban areas) (CORINE)	Area size of the nearest polygon. Total of all categories of green spaces (forests + green urban areas) from CORINE data.	N/A	7
physenv_cn_ne_ttbs_as_0	decimal	Area size of total blue spaces (water + wetlands) (CORINE)	Area size of the nearest polygon. Total of all categories of blue spaces (water + wetlands) from CORINE data.	N/A	7
physenv_cn_ne_ttbsgr_as_0	decimal	Area size of total blue and green spaces (water, wetlands, forests, green urban areas) (CORINE)	Area size of the nearest polygon. Total of both green and blue spaces (water, wetlands, forests, green urban areas) from CORINE data.	N/A	7
physenv_ua_be_facil_0	decimal	Distance to facilities (Urban Atlas)	Absolute distance to the nearest polygon marked as sports & leisure facilities from Urban Atlas data.	N/A	7
physenv_ua_be_facil_as_0	decimal	Area size of facilities (Urban Atlas)	Area size of the nearest polygon marked as sports & leisure facilities from Urban Atlas data.	N/A	7
physenv_cn_be_facil_0	decimal	Distance to facilities (CORINE)	Absolute distance to the nearest polygon marked as sports & leisure facilities from CORINE data.	N/A	7
physenv_cn_be_facil_as_0	decimal	Area size of facilities (CORINE)	Area size of the nearest polygon marked as sports & leisure facilities from CORINE data.	N/A	7
physenv_ua_bf_grurb100_0	decimal	Area size of green urban areas within 100m buffer (Urban Atlas)	Area size in m² of green urban areas within 100m buffer from Urban Atlas data.	N/A	7
physenv_ua_bf_forest100_0	decimal	Area size of forest within 100m buffer (Urban Atlas)	Area size in m² of forest within 100m buffer from Urban Atlas data.	N/A	7
physenv_ua_bf_water100_0	decimal	Area size of water within 100m buffer (Urban Atlas)	Area size in m² of water 100m buffer from Urban Atlas data.	N/A	7
physenv_ua_bf_agri100_0	decimal	Area size of agricultural land within 100m buffer (Urban Atlas)	Area size in m² of agricultural land within 100m buffer from Urban Atlas data.	N/A	7
physenv_ua_bf_ttgr100_0	decimal	Area size of total green spaces (forests + green urban areas) within 100m buffer (Urban Atlas)	Area size in m² of total green spaces (forests + green urban areas) within 100m buffer from Urban Atlas data.	N/A	7
physenv_ua_bf_ttbs100_0	decimal	Area size of total blue spaces (water + wetlands) within 100m buffer (Urban Atlas)	Area size in m² of total blue spaces (water + wetlands) within 100m buffer from Urban Atlas data.	N/A	7
physenv_ua_bf_ttbsgr100_0	decimal	Area size of total blue and green spaces (water, wetlands, forests, green urban areas) within 100m buffer (Urban Atlas)	Area size in m² of total blue and green spaces (water, wetlands, forests, green urban areas) within 100m buffer from Urban Atlas data.	N/A	7
physenv_cn_bf_grurb100_0	decimal	Area size of green urban areas within 100m buffer (CORINE)	Area size in m² of green urban areas within 100m buffer from CORINE data.	N/A	7
physenv_cn_bf_forest100_0	decimal	Area size of forest within 100m buffer (CORINE)	Area size in m² of forest within 100m buffer from CORINE data.	N/A	7
physenv_cn_bf_water100_0	decimal	Area size of water within 100m buffer 1990 (CORINE)	Area size in m² of water within 100m buffer from CORINE data.	N/A	7
physenv_cn_bf_agri100_0	decimal	Area size of agricultural land within 100m buffer (CORINE)	Area size in m² of agricultural land within 100m buffer from CORINE data.	N/A	7

Variable name	Format	Label	Description	Categories	# Events
physenv_cn_bf_ttgr100_0	decimal	Area size of total green spaces (forests + green urban areas) within 100m buffer (CORINE)	Area size in m² of total green spaces (forests + green urban areas) within 100m buffer from CORINE data.	N/A	7
physenv_cn_bf_ttbs100_0	decimal	Area size of total blue spaces (water + wetlands) within 100m buffer (CORINE)	Area size in m² of total blue spaces (water + wetlands) 100m buffer from CORINE data.	N/A	7
physenv_cn_bf_ttbsgr100_0	decimal	Area size of total blue and green spaces (water, wetlands, forests, green urban areas) within 100m buffer (CORINE)	Area size in m² of total blue and green spaces (water, wetlands, forests, green urban areas) within 100m buffer from CORINE data.	N/A	7
physenv_ua_bf_grurb400_0	decimal	Area size of green urban areas within 400m buffer (Urban Atlas)	Area size in m² of green urban areas within 400m buffer from Urban Atlas data.	N/A	7
physenv_ua_bf_forest400_0	decimal	Area size of forest within 400m buffer (Urban Atlas)	Area size in m² of forest within 400m buffer from Urban Atlas data.	N/A	7
physenv_ua_bf_water400_0	decimal	Area size of water within 400m buffer (Urban Atlas)	Area size in m² of water within 400m buffer from Urban Atlas data.	N/A	7
physenv_ua_bf_agri400_0	decimal	Area size of agricultural land within 400m buffer (Urban Atlas)	Area size in m² of agricultural land within 400m buffer from Urban Atlas data.	N/A	7
physenv_ua_bf_ttgr400_0	decimal	Area size of total green spaces (forests + green urban areas) within 400m buffer (Urban Atlas)	Area size in m² of total green spaces (forests + green urban areas) within 400m buffer from Urban Atlas data.	N/A	7
physenv_ua_bf_ttbs400_0	decimal	Area size of total blue spaces (water + wetlands) within 400m buffer (Urban Atlas)	Area size in m² of total blue spaces (water + wetlands) within 400m buffer from Urban Atlas data.	N/A	7
physenv_ua_bf_ttbsgr400_0	decimal	Area size of total blue and green spaces (water, wetlands, forests, green urban areas) within 400m buffer (Urban Atlas)	Area size in m² of total blue and green spaces (water, wetlands, forests, green urban areas) within 400m buffer from Urban Atlas data.	N/A	7
physenv_cn_bf_grurb400_0	decimal	Area size of green urban areas within 400m buffer (CORINE)	Area size in m² of green urban areas within 400m buffer from CORINE data.	N/A	7
physenv_cn_bf_forest400_0	decimal	Area size of forest within 400m buffer (CORINE)	Area size in m² of forest within 400m buffer from CORINE data.	N/A	7
physenv_cn_bf_water400_0	decimal	Area size of water within 400m buffer (CORINE)	Area size in m² of water within 400m buffer from CORINE data.	N/A	7
physenv_cn_bf_agri400_0	decimal	Area size of agricultural land within 400m buffer (CORINE)	Area size in m² of agricultural land within 400m buffer from CORINE data.	N/A	7
physenv_cn_bf_ttgr400_0	decimal	Area size of total green spaces (forests + green urban areas) within 400m buffer (CORINE)	Area size in m² of total green spaces (forests + green urban areas) within 400m buffer from CORINE data.	N/A	7
physenv_cn_bf_ttbs400_0	decimal	Area size of total blue spaces (water + wetlands) within 400m buffer (CORINE)	Area size in m² of total blue spaces (water + wetlands) within 400m buffer from CORINE data.	N/A	7
physenv_cn_bf_ttbsgr400_0	decimal	Area size of total blue and green spaces (water, wetlands, forests, green urban areas) within 400m buffer (CORINE)	Area size in m² of total blue and green spaces (water, wetlands, forests, green urban areas) within 400m buffer from CORINE data.	N/A	7
physenv_ua_bf_facil400_0	decimal	Area size of sports & leisure facilities within 400m buffer (Urban Atlas)	Area size in m² of sports & leisure facilities within 400m buffer from Urban Atlas data.	N/A	7
physenv_cn_bf_facil400_0	decimal	Area size of sports & leisure facilities within 400m buffer (CORINE)	Area size in m² of sports & leisure facilities within 400m buffer from CORINE data.	N/A	7
physenv_ua_bf_grurb800_0	decimal	Area size of green urban areas within 800m buffer (Urban Atlas)	Area size in m² of green urban areas within 800m buffer from Urban Atlas data.	N/A	7
physenv_ua_bf_forest800_0	decimal	Area size of forest within 800m buffer (Urban Atlas)	Area size in m² of forest within 800m buffer from Urban Atlas data.	N/A	7
physenv_ua_bf_water800_0	decimal	Area size of water within 800 m buffer (Urban Atlas)	Area size in m² of water within 800m buffer from Urban Atlas data.	N/A	7

Variable name	Format	Label	Description	Categories	# Events
physenv_ua_bf_agri800_0	decimal	Area size of m² of agricultural land within 800m buffer (Urban Atlas)	Area size in m² of agricultural land within 800m buffer from Urban Atlas data.	N/A	7
physenv_ua_bf_ttgr800_0	decimal	Area size of total green spaces (forests + green urban areas) within 800m buffer (Urban Atlas)	Area size in m² of total green spaces (forests + green urban areas)within 800m buffer from Urban Atlas data.	N/A	7
physenv_ua_bf_ttbs800_0	decimal	Area size of total blue spaces (water + wetlands) within 800m buffer (Urban Atlas)	Area size in m² of blue spaces within 800m buffer from Urban Atlas data.	N/A	7
physenv_ua_bf_ttbsgr800_0	decimal	Area size of total blue and green spaces (water, wetlands, forests, green urban areas) within 800m buffer (Urban Atlas)	Area size in m² of total blue and green spaces (water, wetlands, forests, green urban areas) within 800m buffer from Urban Atlas data.	N/A	7
physenv_cn_bf_grurb800_0	decimal	Area size of green urban areas within 800m buffer (CORINE)	Area size in m² of green urban areas within 800m buffer from CORINE data.	N/A	7
physenv_cn_bf_forest800_0	decimal	Area size of forest within 800m buffer (CORINE)	Area size in m² of forest within 800m buffer from CORINE data.	N/A	7
physenv_cn_bf_water800_0	decimal	Area size of water within 800m buffer (CORINE)	Area size in m² of water within 800m buffer from CORINE data.	N/A	7
physenv_cn_bf_agri800_0	decimal	Area size of agricultural land within 800m buffer (CORINE)	Area size in m² of agricultural land within 800m buffer from CORINE data.	N/A	7
physenv_cn_bf_ttgr800_0	decimal	Area size of total green spaces (forests + green urban areas) within 800m buffer (CORINE)	Area size in m² of total green spaces (forests + green urban areas) within 800m buffer from CORINE data.	N/A	7
physenv_cn_bf_ttbs800_0	decimal	Area size of total blue spaces (water + wetlands) within 800m buffer (CORINE)	Area size in m² of total blue spaces (water + wetlands) within 800m buffer from CORINE data.	N/A	7
physenv_cn_bf_ttbsgr800_0	decimal	Area size of total blue and green spaces (water, wetlands, forests, green urban areas) within 800m buffer (CORINE)	Area size in m² of total blue and green spaces (water, wetlands, forests, green urban areas) within 800m buffer from CORINE data.	N/A	7
physenv_ua_bf_facil800_0	decimal	Area size of sports & leisure facilities within 800m buffer (Urban Atlas)	Area size in m² of sports & leisure facilities within 800m buffer from Urban Atlas data.	N/A	7
physenv_cn_bf_facil800_0	decimal	Area size of sports & leisure facilities within 800m buffer (CORINE)	Area size in m² of sports & leisure facilities within 800m buffer from CORINE data.	N/A	7
physenv_ua_bf_grurb1000_0	decimal	Area size of green urban areas within 1000m buffer (Urban Atlas)	Area size in m² of green urban areas within 1000m buffer from Urban Atlas data.	N/A	7
physenv_ua_bf_forest1000_0	decimal	Area size of forest within 1000m buffer (Urban Atlas)	Area size in m² of forest within 1000m buffer from Urban Atlas data.	N/A	7
physenv_ua_bf_water1000_0	decimal	Area size of water within 1000m buffer (Urban Atlas)	Area size in m² of water within 1000m buffer from Urban Atlas data.	N/A	7
physenv_ua_bf_agri1000_0	decimal	Area size of agricultural land within 1000m buffer (Urban Atlas)	Area size in m² of agricultural land within 1000m buffer from Urban Atlas data.	N/A	7
physenv_ua_bf_ttgr1000_0	decimal	Area size of total green spaces (forests + green urban areas) within 1000m buffer (Urban Atlas)	Area size in m² of total green spaces (forests + green urban areas) within 1000m buffer from Urban Atlas data.	N/A	7
physenv_ua_bf_ttbs1000_0	decimal	Area size of total blue spaces (water + wetlands) within 1000m buffer (Urban Atlas)	Area size in m² of total blue spaces (water + wetlands) within 1000m buffer from Urban Atlas data.	N/A	7
physenv_ua_bf_ttbsgr1000_0	decimal	Area size of total blue and green spaces (water, wetlands, forests, green urban areas) within 1000m buffer (Urban Atlas)	Area size in m² of total blue and green spaces (water, wetlands, forests, green urban areas) within 1000m buffer from Urban Atlas data.	N/A	7
physenv_cn_bf_grurb1000_0	decimal	Area size of green urban areas within 1000m buffer (CORINE)	Area size in m² of green urban areas within 1000m buffer from CORINE data.	N/A	7
physenv_cn_bf_forest1000_0	decimal	Area size of forest within 1000m buffer (CORINE)	Area size in m² of forest within 1000m buffer from CORINE data.	N/A	7

Variable name	Format	Label	Description	Categories	# Events
physenv_cn_bf_water1000_0	decimal	Area size of water within 1000m buffer (CORINE)	Area size in m² of water within 1000m buffer from CORINE data.	N/A	7
physenv_cn_bf_agri1000_0	decimal	Area size of agricultural land within 1000m buffer (CORINE)	Area size in m² of agricultural land within 1000m buffer from CORINE data.	N/A	7
physenv_cn_bf_ttgr1000_0	decimal	Area size of total green spaces (forests + green urban areas) within 1000m buffer (CORINE)	Area size in m² of total green spaces (forests + green urban areas) within 1000m buffer from CORINE data.	N/A	7
physenv_cn_bf_ttbs1000_0	decimal	Area size of total blue spaces (water + wetlands) within 1000m buffer (CORINE)	Area size in m² of total blue spaces (water + wetlands) within 1000m buffer from CORINE data.	N/A	7
physenv_cn_bf_ttbsgr1000_0	decimal	Area size of total blue and green spaces (water, wetlands, forests, green urban areas) within 1000m buffer (CORINE)	Area size in m² of total blue and green spaces (water, wetlands, forests, green urban areas) within 1000m buffer from CORINE data.	N/A	7
physenv_ua_bf_facil1000_0	decimal	Area size of sports & leisure facilities within 1000m buffer (Urban Atlas)	Area size in m² of sports & leisure facilities within 1000m buffer from Urban Atlas data.	N/A	7
physenv_cn_bf_facil1000_0	decimal	Area size of sports & leisure facilities within 1000m buffer (CORINE)	Area size in m² of sports & leisure facilities within 1000m buffer from CORINE data.	N/A	7
physenv_ua_bf_grurb1600_0	decimal	Area size of green urban areas within 1600m buffer (Urban Atlas)	Area size in m² of green urban areas within 1600m buffer from Urban Atlas data.	N/A	7
physenv_ua_bf_forest1600_0	decimal	Area size of forest within 1600m buffer (Urban Atlas)	Area size in m² of forest within 1600m buffer from Urban Atlas data.	N/A	7
physenv_ua_bf_water1600_0	decimal	Area size of water within 1600m buffer (Urban Atlas)	Area size in m² of water within 1600m buffer from Urban Atlas data.	N/A	7
physenv_ua_bf_agri1600_0	decimal	Area size of agricultural land within 1600m buffer (Urban Atlas)	Area size in m² of agricultural land within 1600m buffer from Urban Atlas data.	N/A	7
physenv_ua_bf_ttgr1600_0	decimal	Area size of total green spaces (forests + green urban areas) within 1600m buffer (Urban Atlas)	Area size in m² of total green spaces (forests + green urban areas) within 1600m buffer from Urban Atlas data.	N/A	7
physenv_ua_bf_ttbs1600_0	decimal	Area size of total blue spaces (water + wetlands) within 1600m buffer (Urban Atlas)	Area size in m² of total blue spaces (water + wetlands) within 1600m buffer from Urban Atlas data.	N/A	7
physenv_ua_bf_ttbsgr1600_0	decimal	Area size of total blue and green spaces (water, wetlands, forests, green urban areas) within 1600m buffer (Urban Atlas)	Area size in m² of total blue and green spaces (water, wetlands, forests, green urban areas) within 1600m buffer from Urban Atlas data.	N/A	7
physenv_cn_bf_grurb1600_0	decimal	Area size of green urban areas within 1600m buffer (CORINE)	Area size in m² of green urban areas within 1600m buffer from CORINE data.	N/A	7
physenv_cn_bf_forest1600_0	decimal	Area size of forest within 1600m buffer (CORINE)	Area size in m² of forest within 1600m buffer from CORINE data.	N/A	7
physenv_cn_bf_water1600_0	decimal	Area size of water within 1600m buffer (CORINE)	Area size in m² of water within 1600m buffer from CORINE data.	N/A	7
physenv_cn_bf_agri1600_0	decimal	Area size of agricultural land within 1600m buffer (CORINE)	Area size in m² of agricultural land within 1600m buffer from CORINE data.	N/A	7
physenv_cn_bf_ttgr1600_0	decimal	Area size of total green spaces (forests + green urban areas) within 1600m buffer (CORINE)	Area size in m² of total green spaces (forests + green urban areas) within 1600m buffer from CORINE data.	N/A	7
physenv_cn_bf_ttbs1600_0	decimal	Area size of total blue spaces (water + wetlands) within 1600m buffer (CORINE)	Area size in m² of total blue spaces within 1600m buffer from CORINE data.	N/A	7
physenv_cn_bf_ttbsgr1600_0	decimal	Area size of total blue and green spaces (water, wetlands, forests, green urban areas) within 1600m buffer (CORINE)	Area size in m² of total blue and green spaces (water, wetlands, forests, green urban areas) within 1600m buffer from CORINE data.	N/A	7
physenv_ua_bf_facil1600_0	decimal	Area size of sports & leisure facilities within 1600m buffer (Urban Atlas)	Area size in m² of sports & leisure facilities within 1600m buffer from Urban Atlas data.	N/A	7

Variable name	Format	Label	Description	Categories	# Events
physenv_cn_bf_facil1600_0	decimal	Area size of sports & leisure facilities within 1600m buffer (CORINE)	Area size in m² of sports & leisure facilities within 1600m buffer from CORINE data.	N/A	7
physenv_ua_bf_facil3000_0	decimal	Area size of sports & leisure facilities within 3000m buffer (Urban Atlas)	Area size in m² of sports & leisure facilities within 3000m buffer from Urban Atlas data.	N/A	7
physenv_ua_bf_lu100_0	decimal	Land use mix within 100m buffer (Urban Atlas)	Land use mix in m² within a 100m buffer. It is calculated using an entropy formula from Urban Atlas data.	N/A	7
physenv_ua_bf_lu100_bu_0	decimal	Area size of built-up area within 100m buffer (Urban Atlas)	Area size in m² of built-up areas within 100m buffer from Urban Atlas data.	N/A	7
physenv_ua_bf_lu100_ic_0	decimal	Area size of industrial and commercial area within 100m buffer (Urban Atlas)	Area size in m² of industrial and commercial areas within 100m buffer from Urban Atlas data.	N/A	7
physenv_ua_bf_lu100_in_0	decimal	Area size of infrastructure area within 100m buffer (Urban Atlas)	Area size in m² of infrastructure areas within 100m buffer from Urban Atlas data.	N/A	7
physenv_ua_bf_lu100_po_0	decimal	Area size of port area within 100m buffer (Urban Atlas)	Area size in m² of ports (airports and sea ports) within 100m buffer from Urban Atlas data.	N/A	7
physenv_ua_bf_lu100_grurb_0	decimal	Area size of green urban areas (land use) within 100m buffer (Urban Atlas)	Area size in m² of green urban areas (land use) within 100m buffer from Urban Atlas data.	N/A	7
physenv_ua_bf_lu100_facil_0	decimal	Area size of sports & leisure facilities area within 100m buffer (Urban Atlas)	Area size in m² of sports & leisure facilities within 100m buffer from Urban Atlas data.	N/A	7
physenv_ua_bf_lu100_agri_0	decimal	Area size of agricultural land (land use) within 100m buffer (Urban Atlas)	Area size in m² of agricultural land (land use) within 100m buffer from Urban Atlas data.	N/A	7
physenv_ua_bf_lu100_on_0	decimal	Area size of other natural areas within 100m buffer (Urban Atlas)	Area size in m² of other natural areas within 100m buffer from Urban Atlas data.	N/A	7
physenv_ua_bf_lu100_water_0	decimal	Area size of water within 100m buffer (Urban Atlas)	Area size in m² of water within 100m buffer from Urban Atlas data.	N/A	7
physenv_ua_bf_lu100_ot_0	decimal	Area size of other areas within 100m buffer (Urban Atlas)	Area size in m² of other areas within 100m buffer from Urban Atlas data.	N/A	7
physenv_cn_bf_lu100_0	decimal	Land use mix within 100m buffer (CORINE)	Land use mix in m² within a 100m buffer. It is calculated using an entropy formula from CORINE data.	N/A	7
physenv_cn_bf_lu100_bu_0	decimal	Area size of built-up area within 100m buffer (CORINE)	Area size in m² of built-up areas within 100 buffer from CORINE data.	N/A	7
physenv_cn_bf_lu100_ic_0	decimal	Area size of industrial and commercial area within 100m buffer (CORINE)	Area size in m² of industrial and commercial areas within 100m buffer from CORINE data.	N/A	7
physenv_cn_bf_lu100_in_0	decimal	Area size of infrastructure area within 100m buffer (CORINE)	Area size in m² of infrastructure areas within 100m buffer from CORINE data.	N/A	7
physenv_cn_bf_lu100_po_0	decimal	Area size of port area within 100m buffer (CORINE)	Area size in m² of ports (airports and sea ports) within 100m buffer from CORINE data.	N/A	7
physenv_cn_bf_lu100_grurb_0	decimal	Area size of green urban areas (land use) within 100m buffer (CORINE)	Area size in m² of green urban areas (land use) within 100m buffer from CORINE data.	N/A	7
physenv_cn_bf_lu100_facil_0	decimal	Area size of sports & leisure facilities area within 100m buffer (CORINE)	Area size in m² of sports & leisure facilities within 100 buffer from CORINE data.	N/A	7
physenv_cn_bf_lu100_agri_0	decimal	Area size of agricultural land (land use) within 100m buffer (CORINE)	Area size in m² of agricultural land (land use) within 100m buffer from CORINE data.	N/A	7
physenv_cn_bf_lu100_on_0	decimal	Area size of other natural areas within 100m buffer (CORINE)	Area size in m² of other natural areas within 100m buffer from CORINE data.	N/A	7
physenv_cn_bf_lu100_water_0	decimal	Area size of water within 100m buffer (CORINE)	Area size in m² of water within 100m buffer from CORINE data.	N/A	7
physenv_cn_bf_lu100_ot_0	decimal	Area size of other areas within 100m buffer (CORINE)	Area size in m² of other areas within 100m buffer from CORINE data.	N/A	7
physenv_ua_bf_lu400_0	decimal	Land use mix within 400m buffer (Urban Atlas)	Land use mix in m² within a 400mm buffer. It is calculated using an entropy formula from Urban Atlas data.	N/A	7

Variable name	Format	Label	Description	Categories	# Events
physenv_ua_bf_lu400_bu_0	decimal	Area size of built-up area within 400m buffer (Urban Atlas)	Area size in m² of built-up areas within 400m buffer from Urban Atlas data.	N/A	7
physenv_ua_bf_lu400_ic_0	decimal	Area size of industrial and commercial area within 400m buffer (Urban Atlas)	Area size in m² of industrial and commercial areas within 400m buffer from Urban Atlas data.	N/A	7
physenv_ua_bf_lu400_in_0	decimal	Area size of infrastructure area within 400m buffer (Urban Atlas)	Area size in m² of infrastructure areas within 400m buffer from Urban Atlas data.	N/A	7
physenv_ua_bf_lu400_po_0	decimal	Area size of port area within 400m buffer (Urban Atlas)	Area size in m² of ports (airports and sea ports) within 400m buffer from Urban Atlas data.	N/A	7
physenv_ua_bf_lu400_grurb_0	decimal	Area size of green urban areas (land use) within 400m buffer (Urban Atlas)	Area size in m² of green urban areas (land use) within 400m buffer from Urban Atlas data.	N/A	7
physenv_ua_bf_lu400_facil_0	decimal	Area size of sports & leisure facilities area within 400m buffer (Urban Atlas)	Area size in m² of sports & leisure facilities within 400m buffer from Urban Atlas data.	N/A	7
physenv_ua_bf_lu400_agri_0	decimal	Area size of agricultural land (land use) within 400m buffer (Urban Atlas)	Area size in m² of agricultural land (land use) within 400m buffer from Urban Atlas data.	N/A	7
physenv_ua_bf_lu400_on_0	decimal	Area size of other natural areas within 400m buffer (Urban Atlas)	Area size in m² of other natural areas within 400m buffer from Urban Atlas data.	N/A	7
physenv_ua_bf_lu400_water_0	decimal	Area size of water within 400m buffer (Urban Atlas)	Area size in m² of water within 400m buffer from Urban Atlas data.	N/A	7
physenv_ua_bf_lu400_ot_0	decimal	Area size of other areas within 400m buffer (Urban Atlas)	Area size in m² of other areas within 400m buffer from Urban Atlas data.	N/A	7
physenv_cn_bf_lu400_0	decimal	Land use mix within 400m buffer (CORINE)	Land use mix in m² within a 400m buffer. It is calculated using an entropy formula from CORINE data.	N/A	7
physenv_cn_bf_lu400_bu_0	decimal	Area size of built-up area within 400m buffer (CORINE)	Area size in m² of built-up areas within 400m buffer from CORINE data.	N/A	7
physenv_cn_bf_lu400_ic_0	decimal	Area size of industrial and commercial area within 400m buffer (CORINE)	Area size in m² of industrial and commercial areas within 400m buffer from CORINE data.	N/A	7
physenv_cn_bf_lu400_in_0	decimal	Area size of infrastructure area within 400m buffer (CORINE)	Area size in m² of infrastructure areas within 400m buffer from CORINE data.	N/A	7
physenv_cn_bf_lu400_po_0	decimal	Area size of port area within 400m buffer (CORINE)	Area size in m² of ports (airports and sea ports) within 400m buffer from CORINE data.	N/A	7
physenv_cn_bf_lu400_grurb_0	decimal	Area size of green urban areas (land use) within 400m buffer (CORINE)	Area size in m² of green urban areas (land use) within 400m buffer from CORINE data.	N/A	7
physenv_cn_bf_lu400_facil_0	decimal	Area size of sports & leisure facilities area within 400m buffer (CORINE)	Area size in m² of sports & leisure facilities within 400m buffer from CORINE data.	N/A	7
physenv_cn_bf_lu400_agri_0	decimal	Area size of agricultural land (land use) within 400m buffer (CORINE)	Area size in m² of agricultural land (land use) within 400m buffer from CORINE data.	N/A	7
physenv_cn_bf_lu400_on_0	decimal	Area size of other natural areas within 400m buffer (CORINE)	Area size in m² of other natural areas within 400m buffer from CORINE data.	N/A	7
physenv_cn_bf_lu400_water_0	decimal	Area size of water within 400m buffer (CORINE)	Area size in m² of water within 400m buffer from CORINE data.	N/A	7
physenv_cn_bf_lu400_ot_0	decimal	Area size of other areas within 400m buffer (CORINE)	Area size in m² of other areas within 400m buffer from CORINE data.	N/A	7
physenv_ua_bf_lu800_0	decimal	Land use mix within 800m buffer (Urban Atlas)	Land use mix in m² within a 800m buffer. It is calculated using an entropy formula from Urban Atlas data.	N/A	7
physenv_ua_bf_lu800_bu_0	decimal	Area size of built-up area within 800m buffer (Urban Atlas)	Area size in m² of built-up areas within 800m buffer from Urban Atlas data.	N/A	7
physenv_ua_bf_lu800_ic_0	decimal	Area size of industrial and commercial area within 800m buffer (Urban Atlas)	Area size in m² of industrial and commercial areas within 800m buffer from Urban Atlas data.	N/A	7
physenv_ua_bf_lu800_in_0	decimal	Area size of infrastructure area within 800m buffer (Urban Atlas)	Area size in m² of infrastructure areas within 800m buffer from Urban Atlas data.	N/A	7
physenv_ua_bf_lu800_po_0	decimal	Area size of port area within 800m buffer (Urban Atlas)	Area size in m² of ports (airports and sea ports) within 800m buffer from Urban Atlas data.	N/A	7

Variable name	Format	Label	Description	Categories	# Events
physenv_ua_bf_lu800_grurb_0	decimal	Area size of green urban areas (land use) within 800m buffer (Urban Atlas)	Area size in m² of green urban areas (land use) within 800m buffer from Urban Atlas data.	N/A	7
physenv_ua_bf_lu800_facil_0	decimal	Area size of sports & leisure facilities area within 800m buffer (Urban Atlas)	Area size in m² of sports & leisure facilities within 800m buffer from Urban Atlas data.	N/A	7
physenv_ua_bf_lu800_agri_0	decimal	Area size of agricultural land (land use) within 800m buffer (Urban Atlas)	Area size in m² of agricultural land (land use) within 800m buffer from Urban Atlas data.	N/A	7
physenv_ua_bf_lu800_on_0	decimal	Area size of other natural areas within 800m buffer (Urban Atlas)	Area size in m² of other natural areas within 800m buffer from Urban Atlas data.	N/A	7
physenv_ua_bf_lu800_water_0	decimal	Area size of water within 800m buffer (Urban Atlas)	Area size in m² of water within 800m buffer from Urban Atlas data.	N/A	7
physenv_ua_bf_lu800_ot_0	decimal	Area size of other areas within 800m buffer (Urban Atlas)	Area size in m² of other areas within 800m buffer from Urban Atlas data.	N/A	7
physenv_cn_bf_lu800_0	decimal	Land use mix within 800m buffer (CORINE)	Land use mix in m² within a 800m buffer. It is calculated using an entropy formula from CORINE data.	N/A	7
physenv_cn_bf_lu800_bu_0	decimal	Area size of built-up area within 800m buffer (CORINE)	Area size in m² of built-up areas within 800m buffer from CORINE data.	N/A	7
physenv_cn_bf_lu800_ic_0	decimal	Area size of industrial and commercial area within 800m buffer (CORINE)	Area size in m² of industrial and commercial areas within 800m buffer from CORINE data.	N/A	7
physenv_cn_bf_lu800_in_0	decimal	Area size of infrastructure area within 800m buffer (CORINE)	Area size in m² of infrastructure areas within 800m buffer from CORINE data.	N/A	7
physenv_cn_bf_lu800_po_0	decimal	Area size of port area within 800m buffer (CORINE)	Area size in m² of ports (airports and sea ports) within 800m buffer from CORINE data.	N/A	7
physenv_cn_bf_lu800_grurb_0	decimal	Area size of green urban areas (land use) within 800m buffer (CORINE)	Area size in m² of green urban areas (land use) within 800m buffer from CORINE data.	N/A	7
physenv_cn_bf_lu800_facil_0	decimal	Area size of sports & leisure facilities area within 800m buffer (CORINE)	Area size in m² of sports & leisure facilities within 800m buffer from CORINE data.	N/A	7
physenv_cn_bf_lu800_agri_0	decimal	Area size of agricultural land (land use) within 800m buffer (CORINE)	Area size in m² of agricultural land (land use) within 800m buffer from CORINE data.	N/A	7
physenv_cn_bf_lu800_on_0	decimal	Area size of other natural areas within 800m buffer (CORINE)	Area size in m² of other natural areas within 800m buffer from CORINE data.	N/A	7
physenv_cn_bf_lu800_water_0	decimal	Area size of water within 800m buffer (CORINE)	Area size in m² of water within 800m buffer from CORINE data.	N/A	7
physenv_cn_bf_lu800_ot_0	decimal	Area size of other areas within 800m buffer (CORINE)	Area size in m² of other areas within 800m buffer from CORINE data.	N/A	7
physenv_ua_bf_lu1000_0	decimal	Land use mix within 1000m buffer (Urban Atlas)	Land use mix in m² within a 1000m buffer. It is calculated using an entropy formula from Urban Atlas data.	N/A	7
physenv_ua_bf_lu1000_bu_0	decimal	Area size of built-up area within 1000m buffer (Urban Atlas)	Area size in m² of built-up areas within 1000m buffer from Urban Atlas data.	N/A	7
physenv_ua_bf_lu1000_ic_0	decimal	Area size of industrial and commercial area within 1000m buffer (Urban Atlas)	Area size in m² of industrial and commercial areas within 1000m buffer from Urban Atlas data.	N/A	7
physenv_ua_bf_lu1000_in_0	decimal	Area size of infrastructure area within 1000m buffer (Urban Atlas)	Area size in m² of infrastructure areas within 1000m buffer from Urban Atlas data.	N/A	7
physenv_ua_bf_lu1000_po_0	decimal	Area size of port area within 1000m buffer (Urban Atlas)	Area size in m² of ports (airports and sea ports) within 1000m buffer from Urban Atlas data.	N/A	7
physenv_ua_bf_lu1000_grurb_0	decimal	Area size of green urban areas (land use) within 1000m buffer (Urban Atlas)	Area size in m² of green urban areas (land use) within 1000m buffer from Urban Atlas data.	N/A	7
physenv_ua_bf_lu1000_facil_0	decimal	Area size of sports & leisure facilities area within 1000m buffer (Urban Atlas)	Area size in m² of sports & leisure facilities within 1000m buffer from Urban Atlas data.	N/A	7
physenv_ua_bf_lu1000_agri_0	decimal	Area size of agricultural land (land use) within 1000m buffer (Urban Atlas)	Area size in m² of agricultural land (land use) within 1000m buffer from Urban Atlas data.	N/A	7

Variable name	Format	Label	Description	Categories	# Events
physenv_ua_bf_lu1000_on_0	decimal	Area size of other natural areas within 1000m buffer (Urban Atlas)	Area size in m² of other natural areas within 1000m buffer from Urban Atlas data.	N/A	7
physenv_ua_bf_lu1000_water_0	decimal	Area size of water within 1000m buffer (Urban Atlas)	Area size in m² of water within 1000m buffer from Urban Atlas data.	N/A	7
physenv_ua_bf_lu1000_ot_0	decimal	Area size of other areas within 1000m buffer (Urban Atlas)	Area size in m² of other areas within 1000m buffer from Urban Atlas data.	N/A	7
physenv_cn_bf_lu1000_0	decimal	Land use mix within 1000m buffer (CORINE)	Land use mix in m² within a 1000m buffer. It is calculated using an entropy formula from CORINE data.	N/A	7
physenv_cn_bf_lu1000_bu_0	decimal	Area size of built-up area within 1000m buffer (CORINE)	Area size in m² of built-up areas within 1000m buffer from CORINE data.	N/A	7
physenv_cn_bf_lu1000_ic_0	decimal	Area size of industrial and commercial area within 1000m buffer (CORINE)	Area size in m² of industrial and commercial areas within 1000m buffer from CORINE data.	N/A	7
physenv_cn_bf_lu1000_in_0	decimal	Area size of infrastructure area within 1000m buffer (CORINE)	Area size in m² of infrastructure areas within 1000m buffer from CORINE data.	N/A	7
physenv_cn_bf_lu1000_po_0	decimal	Area size of port area within 1000m buffer (CORINE)	Area size in m² of ports (airports and sea ports) within 1000m buffer from CORINE data.	N/A	7
physenv_cn_bf_lu1000_grurb_0	decimal	Area size of green urban areas (land use) within 1000m buffer (CORINE)	Area size in m² of green urban areas (land use) within 1000m buffer from CORINE data.	N/A	7
physenv_cn_bf_lu1000_facil_0	decimal	Area size of sports & leisure facilities areas within 1000m buffer (CORINE)	Area size in m² of sports & leisure facilities within 1000m buffer from CORINE data.	N/A	7
physenv_cn_bf_lu1000_agri_0	decimal	Area size of agricultural land (land use) within 1000m buffer (CORINE)	Area size in m² of agricultural land (land use) within 1000m buffer from CORINE data.	N/A	7
physenv_cn_bf_lu1000_on_0	decimal	Area size of other natural areas within 1000m buffer (CORINE)	Area size in m² of other natural areas within 1000m buffer from CORINE data.	N/A	7
physenv_cn_bf_lu1000_water_0	decimal	Area size of water within 1000m buffer (CORINE)	Area size in m² of water within 1000m buffering 1990 from CORINE data.	N/A	7
physenv_cn_bf_lu1000_ot_0	decimal	Area size of other areas within 1000m buffer (CORINE)	Area size in m² of other areas within 1000m buffer from CORINE data.	N/A	7
physenv_ua_bf_lu1600_0	decimal	Land use mix within 1600m buffer (Urban Atlas)	Land use mix in m² within a 1600m buffer. It is calculated using an entropy formula from Urban Atlas data.	N/A	7
physenv_ua_bf_lu1600_bu_0	decimal	Area size of built-up areas within 1600m buffer (Urban Atlas)	Area size in m² of built-up areas within 1600m buffer from Urban Atlas data.	N/A	7
physenv_ua_bf_lu1600_ic_0	decimal	Area size of industrial and commercial areas within 1600m buffer (Urban Atlas)	Area size in m² of industrial and commercial areas within 1600m buffer from Urban Atlas data.	N/A	7
physenv_ua_bf_lu1600_in_0	decimal	Area size of infrastructure areas within 1600m buffer (Urban Atlas)	Area size in m² of infrastructure areas within 1600m buffer from Urban Atlas data.	N/A	7
physenv_ua_bf_lu1600_po_0	decimal	Area size of port area within 1600m buffer (Urban Atlas)	Area size in m² of ports (airports and sea ports) within 1600m buffer from Urban Atlas data.	N/A	7
physenv_ua_bf_lu1600_grurb_0	decimal	Area size of green urban areas (land use) within 1600m buffer (Urban Atlas)	Area size in m² of green urban areas (land use) within 1600m buffer from Urban Atlas data.	N/A	7
physenv_ua_bf_lu1600_facil_0	decimal	Area size of sports & leisure facilities areas within 1600m buffer (Urban Atlas)	Area size in m² of sports & leisure facilities within 1600m buffer from Urban Atlas data.	N/A	7
physenv_ua_bf_lu1600_agri_0	decimal	Area size of agricultural land (land use) within 1600m buffer (Urban Atlas)	Area size in m² of agricultural land (land use) within 1600m buffer from Urban Atlas data.	N/A	7
physenv_ua_bf_lu1600_on_0	decimal	Area size of other natural areas within 1600m buffer (Urban Atlas)	Area size in m² of other natural areas within 1600m buffer from Urban Atlas data.	N/A	7
physenv_ua_bf_lu1600_water_0	decimal	Area size of water within 1600m buffer (Urban Atlas)	Area size in m² of water within 1600m buffer from Urban Atlas data.	N/A	7
physenv_ua_bf_lu1600_ot_0	decimal	Area size of other areas within 1600m buffer (Urban Atlas)	Area size in m² of other areas within 1600m buffer from Urban Atlas data.	N/A	7

Variable name	Format	Label	Description	Categories	# Events
physenv_cn_bf_lu1600_0	decimal	Land use mix within 1600m buffer (CORINE)	Land use mix in m² within a 1600m buffer. It is calculated using an entropy formula from CORINE data.	N/A	7
physenv_cn_bf_lu1600_bu_0	decimal	Area size of built-up areas within 1600m buffer (CORINE)	Area size in m² of built-up areas within 1600m buffer from CORINE data.	N/A	7
physenv_cn_bf_lu1600_ic_0	decimal	Area size of industrial and commercial areas within 1600m buffer (CORINE)	Area size in m² of industrial and commercial areas within 1600m buffer from CORINE data.	N/A	7
physenv_cn_bf_lu1600_in_0	decimal	Area size of infrastructure areas within 1600m buffer (CORINE)	Area size in m² of infrastructure areas within 1600m buffer from CORINE data.	N/A	7
physenv_cn_bf_lu1600_po_0	decimal	Area size of port area within 1600m buffer (CORINE)	Area size in m² of ports (airports and sea ports) within 1600m buffer from CORINE data.	N/A	7
physenv_cn_bf_lu1600_grurb_0	decimal	Area size of green urban areas (land use) within 1600m buffer (CORINE)	Area size in m² of green urban areas (land use) within 1600m buffer from CORINE data.	N/A	7
physenv_cn_bf_lu1600_facil_0	decimal	Area size of sports & leisure facilities areas within 1600m buffer (CORINE)	Area size in m² of sports & leisure facilities within 1600m buffer from CORINE data.	N/A	7
physenv_cn_bf_lu1600_agri_0	decimal	Area size of agricultural land (land use) within 1600m buffer (CORINE)	Area size in m² of agricultural land (land use) within 1600m buffer from CORINE data.	N/A	7
physenv_cn_bf_lu1600_on_0	decimal	Area size of other natural areas within 1600m buffer (CORINE)	Area size in m² of other natural areas within 1600m buffer from CORINE data.	N/A	7
physenv_cn_bf_lu1600_water_0	decimal	Area size of water within 1600m buffer (CORINE)	Area size in m² of water within 1600m buffer from CORINE data.	N/A	7
physenv_cn_bf_lu1600_ot_0	decimal	Area size of other areas within 1600m buffer (CORINE)	Area size in m² of other areas within 1600m buffer from CORINE data.	N/A	7
physenv_ua_urbcy_0	integer	Urbanicity grade (Urban Atlas)	Urbanicity grade based on the classes from the urban atlas. This is a categorical variable with 5 classes ranging from continuous urban to dense, medium, low, and very low density from Urban Atlas data.	1 = Continuous urban fabric (S.L. > 80%)<br>2 = Discontinuous dense urban fabric (S.L.: 50% - 80%)<br>3 = Discontinuous medium density urban fabric (S.L.: 30% - 50%)<br>4 = Discontinuous low density urban fabric (S.L.: 10% - 30%)<br>5 = Discontinuous very low density urban fabric (S.L.: < 10%)	7
physenv_cn_urbcy_0	integer	Urbanicity grade (CORINE)	Urbanicity grade based on the classes from the CORINE. This is a categorical variable with 2 classes being from continuous urban to dense, medium, low, and very low density from CORINE data.	0 = Continuous urban fabric (S.L. > 80%)<br>1 = Discontinuous urban fabric (S.L.: 30% - 80%)	7
**Administrative information**					
baseline_yr	text	Baseline Year	Baseline Year	N/A	1
followup1_yr	text	Follow-up 1 year	Follow-up 1 year	N/A	1
followup2_yr	text	Follow-up 2 year	Follow-up 2 year	N/A	1
followup3_yr	text	Follow-up 3 year	Follow-up 3 year	N/A	1
followup4_yr	text	Follow-up 4 year	Follow-up 4 year	N/A	1
followup5_yr	text	Follow-up 5 year	Follow-up 5 year	N/A	1
followup6_yr	text	Follow-up 6 year	Follow-up 6 year	N/A	1
t1	text	Years between follow-up 1 and baseline	Years between follow-up 1 and baseline	N/A	1
t2	text	Years between follow-up 2 and follow-up 1	Years between follow-up 2 and follow-up 1	N/A	1
t3	text	Years between follow-up 3 and follow-up 2	Years between follow-up 3 and follow-up 2	N/A	1

Variable name	Format	Label	Description	Categories	# Events
t4	text	Years between follow-up 4 and follow-up 3	Years between follow-up 4 and follow-up 3	N/A	1
t5	text	Years between follow-up 5 and follow-up 4	Years between follow-up 5 and follow-up 4	N/A	1
t6	text	Years between follow-up 6 and follow-up 5	Years between follow-up 6 and follow-up 5	N/A	1
